# Proteome-wide analysis reveals potential therapeutic targets for Colorectal cancer: a two-sample mendelian randomization study

**DOI:** 10.1186/s12885-023-11669-6

**Published:** 2023-12-04

**Authors:** Yi-Xin Cai, Yi-Qing Wu, Jie Liu, Huanle Pan, Wenhai Deng, Weijian Sun, Congying Xie, Xiu-Feng Huang

**Affiliations:** 1 Zhejiang Provincial Clinical Research Center for Pediatric Disease, The Second Affiliated Hospital and Yuying Children’s Hospital of Wenzhou Medical University, Wenzhou, Zhejiang China; 2 Department of Pediatrics, the Second School of Medicine, The Second Affiliated Hospital and Yuying Children’s Hospital of Wenzhou Medical University, Wenzhou, Zhejiang China; 3 Information Technology Center, Wenzhou Medical University, Wenzhou, Zhejiang China; 4 Department of Radiation and Medical Oncology, The First Affiliated Hospital of Wenzhou Medical University, Wenzhou, Zhejiang China; 5 Key Laboratory of Laboratory Medicine, School of Laboratory Medicine and Life Sciences, Ministry of Education, Wenzhou Medical University, Wenzhou, Zhejiang China; 6 Department of Gastrointestinal Surgery, The Second Affiliated Hospital and Yuying Children’s Hospital of Wenzhou Medical University, Wenzhou, Zhejiang China; 7 Department of Radiation and Medical Oncology, The Second Affiliated Hospital and Yuying Children’s Hospital of Wenzhou Medical University, Wenzhou, Zhejiang China

**Keywords:** Plasma proteins, Colorectal cancer, Mendelian randomization, Risk factors, Causal effects, Phenome-wide association study

## Abstract

**Background:**

Colorectal cancer (CRC) is a leading cause of cancer-related mortality, highlighting an unmet clinical need for more effective therapies. This study aims to evaluate the causal relationship between 4,489 plasma proteins and CRC to identify potential therapeutic targets for CRC.

**Methods:**

We conducted two-sample Mendelian randomization (MR) analysis to examine the causal effects of plasma proteins on CRC. Mediation analysis was performed to assess the indirect effects of plasma proteins on CRC through associated risk factors. In addition, we conducted a phenome-wide association study using the UK Biobank dataset to examine associations between these plasma proteins and other phenotypes.

**Results:**

Out of 4,489 plasma proteins, MR analysis revealed causal associations with CRC for 23 proteins, including VIMP, MICB, TNFRSF11B, C5orf38 and SLC5A8. Our findings also confirm the associations between reported risk factors and CRC. Mediation analysis identified mediating effects of proteins on CRC outcomes through risk factors. Furthermore, MR analysis identified 154 plasma proteins are causally linked to at least one CRC risk factor.

**Conclusions:**

Our study evaluated the causal relationships between plasma proteins and CRC, providing a more complete understanding of potential therapeutic targets for CRC.

**Supplementary Information:**

The online version contains supplementary material available at 10.1186/s12885-023-11669-6.

## Introduction

Colorectal cancer (CRC) is a significant global health concern. In 2020, over 1.9 million people were diagnosed with CRC, and 935,000 deaths were caused by the disease, making it the second most frequent cause of cancer-related deaths and the third largest contributor to newly diagnosed cases [1, 2]. Several factors may augment colorectal cancer risk, including body mass index (BMI). Research has shown that individuals with high BMI are more prone to developing CRC, and the possibility of its onset increases with an increase in BMI [3, 4]. Plasma proteins play fundamental roles in a vast array of biological processes and are frequently dysregulated in diseases, therefore being therapeutically targeted to treat various medical conditions [5–8]. In particular, plasma proteins could potentially function as biomarkers to enable early detection of CRC [9]. Thus, identification of specific plasma proteins associated with CRC can lead to the development of more sensitive and specific biomarkers, enabling earlier detection and treatment at a pre-symptomatic stage. Based on the distinctive molecular characteristics, there can be customized treatment strategies for different patients. Therefore, it is of paramount importance to identify further risk factors, biomarkers, and treatments for CRC.

Genome-wide association studies (GWAS) have detected genetic variants linked to plasma protein levels, referred to as protein quantitative trait loci (pQTLs) [10, 11]. These pQTLs provide an opportunity to employ Mendelian randomization (MR) to assess the causal impact of potential drug targets on the human disease phenome [12–14]. MR is an increasingly popular methodological approach used in epidemiology to infer causal relationships between an exposure and an outcome by utilizing genetic variants as instrumental variables [15, 16]. The use of MR studies in epidemiology allows for stronger causal inference than more conventional forms of analysis. Since genetic variants are determined at birth, reverse causation bias is attenuated, increasing the likelihood that associations established in an MR study are causally interpretable. In brief, MR utilizes genetic variants as instrumental variables to investigate whether a risk factor has a causal impact on a health outcome [17].

In the current study, we performed two-sample MR analysis to examine the causal effects of plasma proteins on CRC by utilizing genetic instrumental variables (IV) (derived from 4489 circulating plasma proteins) and genetic associations of CRC. We assessed the causal relationships between plasma proteins and CRC risk factors, then performed mediation analysis to assess the indirect effects of plasma proteins on CRC through these risk factors. Finally, we performed a phenome-wide association study analysis using UK Biobank data to evaluate the safety of targeting these proteins for CRC treatment.

## Methods

### Study design

**Fig. 1:**
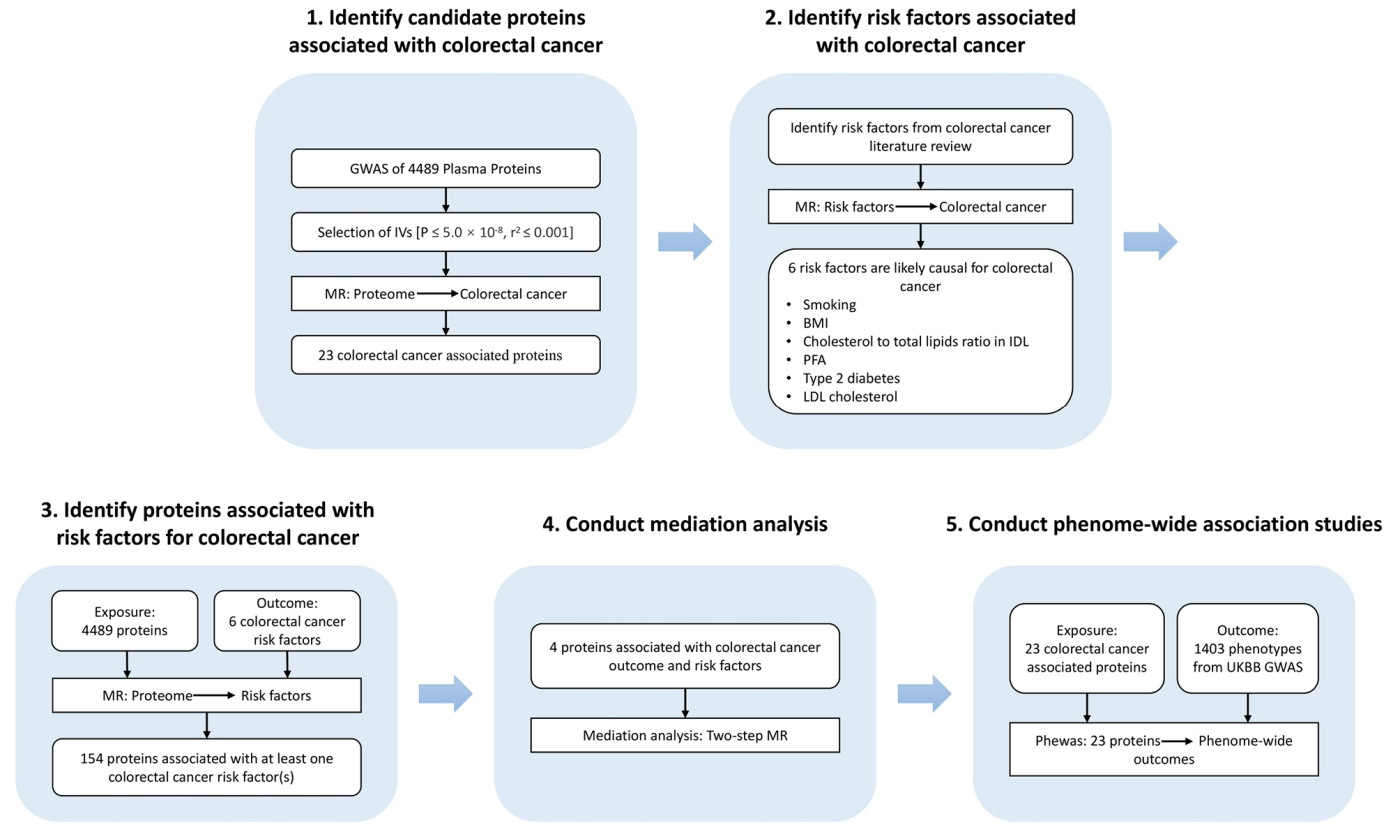
Study overview

This study employed a two-sample MR design, enabling estimation of the causal influence of exposure on outcome using GWAS summary statistics (Fig. 1). MR design is grounded on three core assumptions [18]. Firstly, the robust genetic instrument must display a significant correlation with exposure. Secondly, the genetic instruments can solely indicate an association with the outcome through exposure, also known as the exclusion restriction assumption. Thirdly, it is essential that the genetic instruments do not display any associations with confounding factors of the exposure-outcome relationship.

### GWAS summary statistics for CRC and risk factors

We conducted an initial search of the MR-Base GWAS directory (https://gwas.mrcieu.ac.uk/) using the keywords “colorectal cancer” to obtain GWAS data relevant to the study. We successfully identified a dataset (ieu-b-4965) that was associated with CRC phenotypes. To control for the possible confounding effects of population stratification, we utilized estimated associations between protein IVs, CRC, and risk factors from only individuals with European ancestry.

The secondary outcomes considered in this study were CRC risk factors, which were carefully selected based on a comprehensive literature review [3, 19–22]. A comprehensive search was conducted using the Pubmed database, using the keywords “colorectal cancer” and “risk factors”. Subsequently, we conducted a thorough search for publicly available GWAS summary statistics related to these risk factors. Risk factors for which no data sets were publicly available were eliminated, and six relevant risk factors were ultimately identified. The MR analysis utilized six risk factors: body mass index (BMI), polyunsaturated fatty acids (PFA), type 2 diabetes, cholesterol-to-total-lipids ratio in IDL, LDL cholesterol and smoking. The pQTLs used as IVs for the secondary outcomes were the same as those used for the primary outcomes. Where available, single nucleotide polymorphism (SNP) -outcome effects for all risk factors mentioned above were retrieved from published GWASs.

### GWAS summary statistics for proteins

We obtained GWAS summary statistics for available proteins from the MR-Base NHGRI-EBI GWAS Catalog (https://gwas.mrcieu.ac.uk/) and restricted our analysis to datasets that could be directly downloaded through the TwoSampleMR R package (https://github.com/MRCIEU/TwoSampleMR), which is specifically designed for MR analysis (Table 1). In order to fulfill the first assumption, we mandated that included datasets contain genetic variants that had been validated as statistically significant on a genome-wide level. Ultimately, we retained 4419 proteins (of 4489) for MR analyses by selecting only those that had at least one SNP with an association P-value that met the genome-wide significant threshold of P ≤ 5.0 × 10^− 8^.

**Table 1:** Description of GWAS summary statistics

Trait	Sample size	Number of SNPs	Population	Resource
Colorectal cancer	377,673	11,738,639	European	https://gwas.mrcieu.ac.uk/datasets/ieu-b-4965/
Polyunsaturated fatty acids	114,999	12,321,875	European	https://gwas.mrcieu.ac.uk/datasets/met-d-PUFA/
Cholesterol to total lipids ratio in IDL	115,078	12,321,875	European	https://gwas.mrcieu.ac.uk/datasets/met-d-IDL_C_pct/
Type 2 diabetes	209,439	16,380,426	European	https://gwas.mrcieu.ac.uk/datasets/finn-b-T2D_INCLAVO/
BMI	806,834	NA	European	https://zenodo.org/record/1251813#.ZEJ-Y3ZBzGI
Smoking	NA	NA	European	https://conservancy.umn.edu/handle/11299/241912
LDL cholesterol	70,814	7,892,997	European	https://gwas.mrcieu.ac.uk/datasets/ieu-b-4846/

NA, not applicable

### MR analysis

The R package TwoSampleMR v0.5.6 (https://github.com/MRCIEU/TwoSampleMR) was utilized to conduct MR analyses [18]. In order to ensure the robustness of the exposure association (Assumption 1), only SNPs displaying genome-wide significance (P < 5 × 10^–8^) were considered in MR analysis. In addition, variants correlating strongly with the most significant SNPs were eliminated, ensuring that only linkage disequilibrium (LD)-independent genetic variants were retained for analysis (using clumping r^2^ cut-off = 0.001). Subsequently, included SNPs were standardized, meaning that the effects of a SNP on both the exposure and the outcome must be linked to the same allele. The Inverse-variance weighted (IVW) method utilizes a meta-analysis technique to combine Wald estimates for each SNP, treating them as valid natural experiments. The IVW method was used as the primary analysis in this study to estimate causal relationships between exposures and outcomes. A P value of less than 0.05 was deemed significant.

### Mediation analysis

We conducted mediation analysis on proteins causally associated with both CRC and risk factors to determine their effects on CRC outcomes through the involvement of risk factors. Exposure to the outcome represents the total effect as the combination of direct effects and indirect effects through one or more mediators. In this study, the primary MR, also known as standard univariable MR analysis, captured the total effect. We identified direct and indirect effects by utilizing two-step MR results and estimating the beta of the indirect effect using the Product method. In addition, we used the Delta method to estimate the standard error (SE) and confidence intervals (CIs) of the indirect effect [20].

### Phenome-wide association studies

We further investigated side effects related to the 23 proteins associated with CRC by conducting Phenome-wide association studies (PheWAS) for various diseases. We used summary statistics to analyze the effects of SNPs and outcomes, using a sample size of up to 408,961 individuals from the UK Biobank cohort [19]. The researchers conducted GWAS using SAIGE v.0.29, a generalized mixed model method, to adjust for imbalanced distributions of cases and controls. Phenotypic outcomes of diseases or conditions were defined based on “PheCodes”, a system that organizes codes from the International Classification of Diseases and Related Health Problems (ICD-9/-10) that correspond to specific phenotypic outcomes [21].

## Results

### Identification of candidate proteins associated with Colorectal cancer

**Fig. 2:**
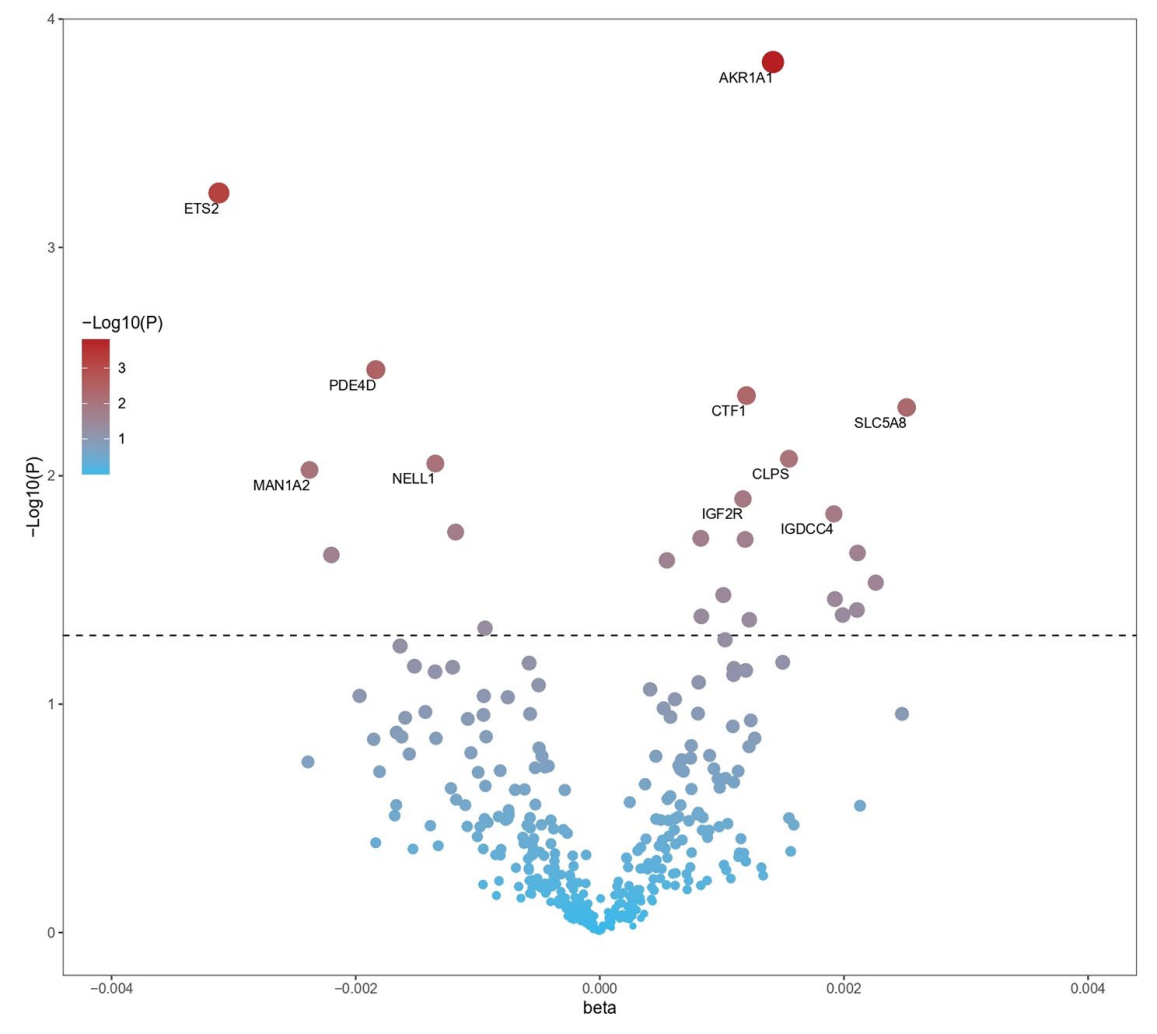
Volcano plot illustrating the effects of candidate plasma proteins associated with colorectal cancer derived from MR analyses using the inverse variance weighted method

**Fig. 3:**
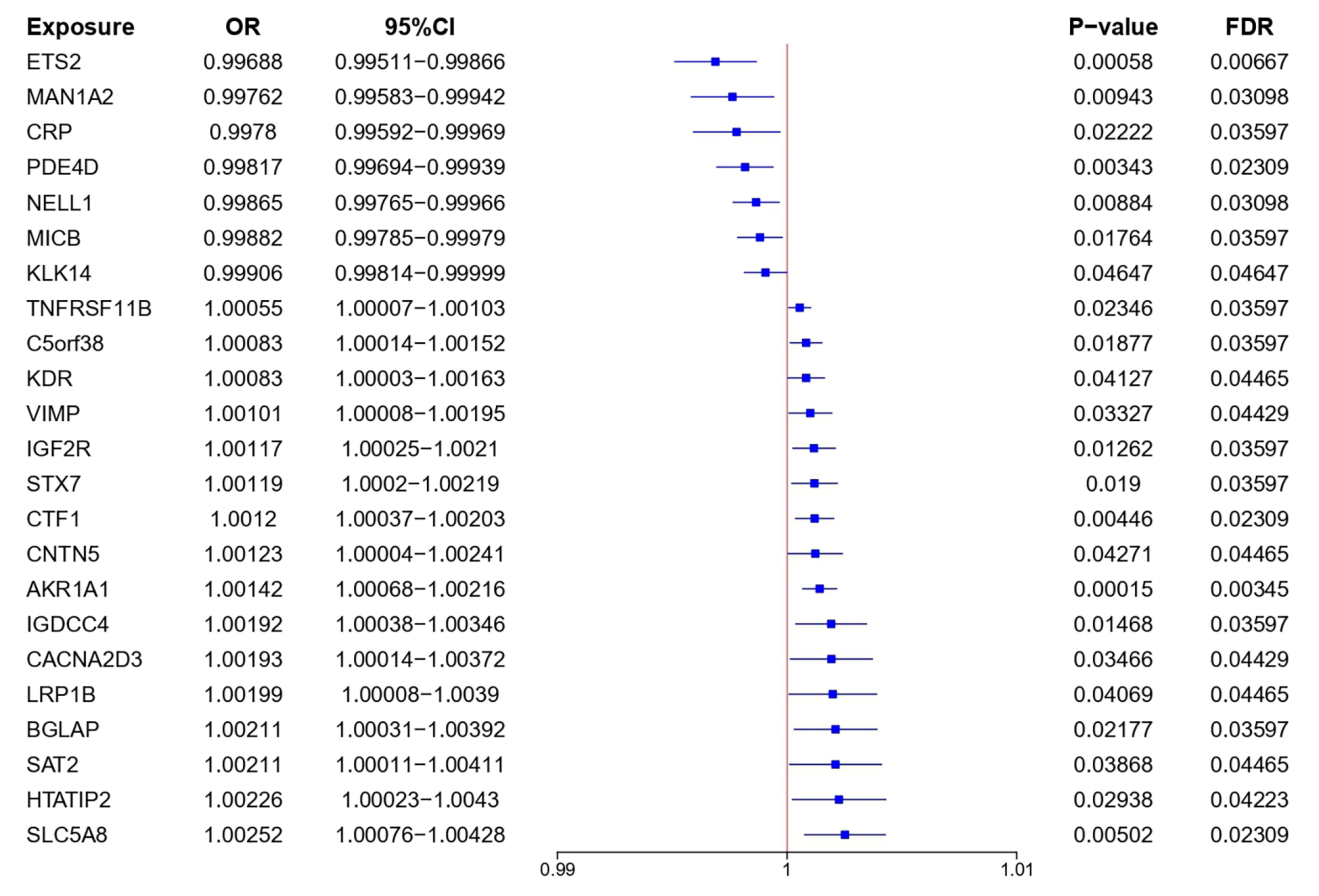
Effect of 23 plasma proteins on CRC. MR analysis of the causal effects of plasma proteins on CRC outcome. CI, confidence interval; OR, odds ratio

In this study, we tested 4489 plasma proteins for causal relationships with CRC outcomes (Figs. 1, 2 and 3 and Table S1). Our MR analysis identified 23 plasma proteins associated with an increased risk of developing CRC: AKR1A1 (aldo-keto reductase family 1 member A1), CTF1 (cardiotrophin 1), SLC5A8 (solute carrier family 5 member 8), IGF2R (insulin like growth factor 2 receptor), IGDCC4 (immunoglobulin superfamily DCC subclass member 4), C5orf38 (IRX2 divergent transcript), STX7 (syntaxin 7), TNFRSF11B (TNF receptor superfamily member 11b), BGLAP (bone gamma-carboxyglutamate protein), HTATIP2 (HIV-1 Tat interactive protein 2), VIMP (selenoprotein S), CACNA2D3 (calcium voltage-gated channel auxiliary subunit alpha2delta 3), SAT2 (spermidine/spermine N1-acetyltransferase family me mber 2), LRP1B (LDL receptor related protein 1B), KDR (kinase insert domain receptor), CNTN5 (contactin 5), ETS2 (ETS proto-oncogene 2, transcription factor), PDE4D (phosphodiesterase 4D), NELL1 (neural EGFL like 1), MAN1A2 (mannosidase alpha class 1 A member 2), MICB (MHC class I polypeptide-related sequence B), CRP (C-reactive protein) and KLK14 (kallikrein related peptidase 14). Increased risk of CRC was associated with higher genetically predicted levels of both AKR1A1 (OR [95% CI] = 1.00142 [1.00068, 1.00216]; P = 0.00015; FDR = 0.00345) and CTF1 (OR [95% CI] = 1.0012 [1.00037, 1.00203]; P = 0.00446; FDR = 0.02309). Higher genetically predicted levels of both ETS2 (OR [95% CI] = 0.99688 [0.99511, 0.99866]; P = 0.00058; FDR = 0.00667) and PDE4D (OR [95% CI] = 0.99817 [0.99694, 0.99939]; P = 0.00343; FDR = 0.02309) were linked to lower CRC risk.

### Identification of risk factors associated with Colorectal cancer

Possible causal mechanisms connecting plasma proteins and CRC were investigated by performing two-step mediation MR analyses of conventional risk factors. Firstly, we examined the causal relationships between plasma proteins and CRC outcome. Secondly, we analyzed the causal relationships between traditional CRC risk factors and CRC outcome. Finally, we conducted mediation analysis to understand the effect of plasma proteins on CRC risk through traditional risk factors.

**Fig. 4:**
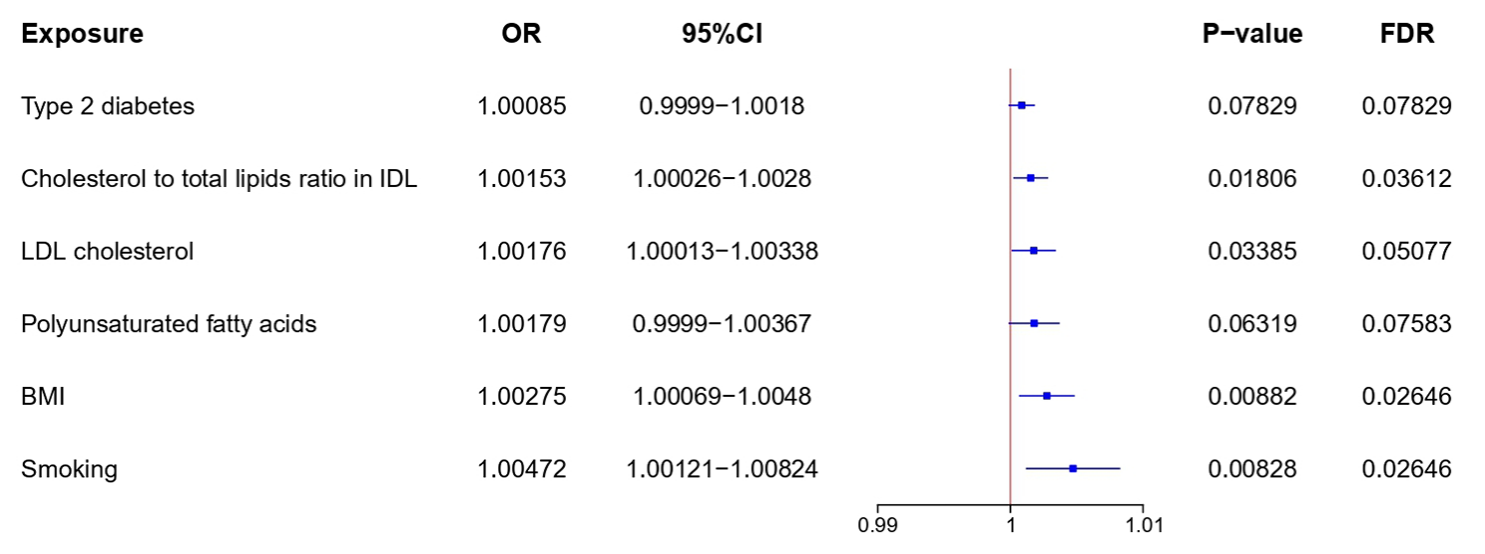
Causal relationships of risk factors to CRC. MR analysis of the causal effects of risk factors on CRC outcome. CI, confidence interval; OR, odds ratio; BMI, body mass index; Smoking, smoking initiation

We extracted instrumental variables for each of the six investigated CRC risk factors, including cholesterol-to-total-lipids ratio in intermediate-density lipoprotein (IDL), smoking, polyunsaturated fatty acids (PFA), body mass index (BMI), Low-Density Lipoprotein Cholesterol (LDL cholesterol), and type 2 diabetes, from publicly available GWAS summary statistics limited to European populations (Fig. 4 and Table S2). Cholesterol-to-total-lipids ratio in IDL (OR [95% CI] = 1.00153 [1.00026, 1.00280]; P = 0.01806; FDR = 0.03612), PFA (OR [95% CI] = 1.00179 [0.99990, 1.00367]; P = 0.06319; FDR = 0.07583), BMI (OR [95% CI] = 1.00275 [1.00069, 1.00480]; P = 0.00882; FDR = 0.02646) and type 2 diabetes (OR [95% CI] = 1.00085 [0.99990, 1.00180]; P = 0.07829; FDR = 0.07829) increased CRC risk.

### Identification of proteins associated with risk factors for Colorectal cancer

We conducted MR analysis to assess the causal relationships between all 4489 plasma proteins and five CRC risk factors. Ultimately, 154 proteins were associated with at least one CRC risk factor: 53 with BMI, 46 with PFA, 27 with smoking, 37 with cholesterol-to-total-lipids ratio in IDL, 28 with LDL cholesterol and 14 with type 2 diabetes (Table S3). Twenty-three proteins associated with CRC were evaluated, and four of them were found to be associated with one or more CRC risk factors. We discovered a positive association between genetically determined higher SLC5A8 levels and increased smoking risk (OR [95% CI]: 1.01420 [1.00337, 1.02515]; P = 0.01003). In addition, higher levels of VIMP and C5orf38, as determined by genetics, were found to be associated with greater PFA risk (VIMP: OR [95% CI] = 1.18137 [1.13235, 1.23250], P = 1.27 × 10^− 14^; C5orf38: OR [95% CI] = 1.13035 [1.09033, 1.17184], P = 2.7 × 10^− 11^). We identified a significant association between genetically higher levels of C5orf38 and VIMP proteins and increased cholesterol-to-total-lipids ratio in IDL (OR [95% CI]: 1.38883 [1.18830, 1.62319]; P = 3.65 × 10^− 5^ and OR [95% CI]: 1.58845 [1.34407, 1.87728]; P = 5.66 × 10^− 8^). The direction of the effects observed for the associations between proteins and risk factors were consistent with those seen for the associations between proteins and CRC, suggesting that these risk factors may mediate protein-CRC associations.

### Mediation analysis of protein effects on CRC through risk factors

**Fig. 5:**
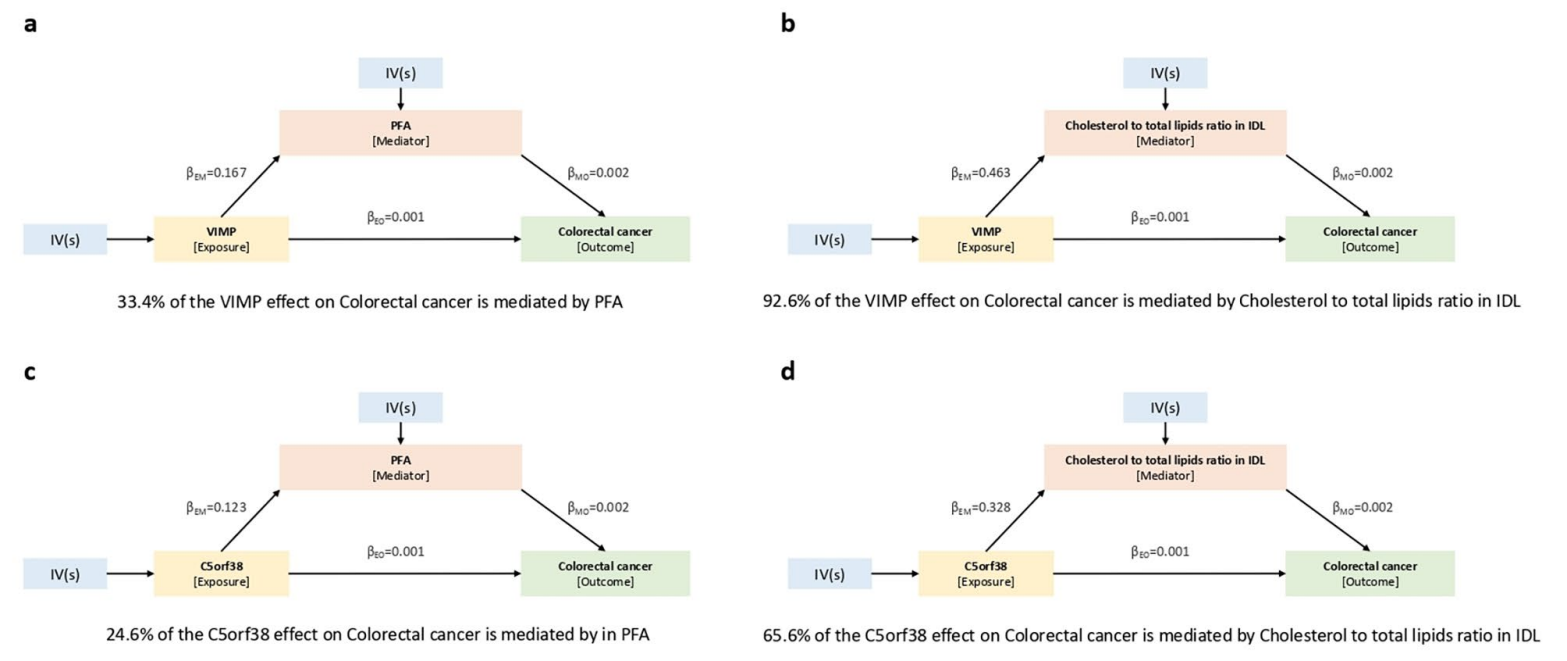
Indirect effects of plasma proteins on CRC via risk factors. Mediation analysis to assess the indirect effects of plasma proteins on CRC through risk factors. a Indirect effect of VIMP on CRC through PFA. b Indirect effect of VIMP on CRC through cholesterol-to-total-lipids ratio in IDL. c Indirect effect of C5orf38 on CRC through PFA. d Indirect effect of C5orf38 on CRC through cholesterol-to-total-lipids ratio in IDL. β_EM_, effects of exposure on mediator; β_MO_, effects of mediator on outcome; β_EO_, effects of exposure on outcome; PFA, polyunsaturated fatty acids

We conducted mediation analysis that used effect estimates obtained from two-step MR analysis, in combination with the total effect estimated in primary MR analysis, to determine the effects of proteins on CRC outcomes as exerted through risk factors. We limited our analysis to four proteins, namely VIMP, SLC5A8, MICB and C5orf38 (Fig. 5, Figure S1 and Figure S2). MR analysis results indicated that these proteins are associated with both CRC and risk factors for CRC. We estimated the indirect effects of proteins on both CRC and risk factors using the product method. Standard error (SE) and confidence intervals (CI) were estimated using the delta method. According to the results of mediation analysis, VIMP had a 33.4% mediation effect on CRC outcomes through PFA and a significant 92.6% effect through cholesterol-to-total-lipids ratio in IDL. Similarly, C5orf38 had a significant mediation effect of 65.6% on CRC outcomes through cholesterol-to-total-lipids ratio in IDL, as well as a 24.6% effect through PFA. In contrast, SLC5A8 had a modest 2.3% mediation effect on CRC outcomes through smoking, and MICB had a 7.4% mediation effect on CRC outcomes through cholesterol-to-total-lipids ratio in IDL.

### Analysis of phenome-wide association studies of proteins associated with CRC

**Fig. 6:**
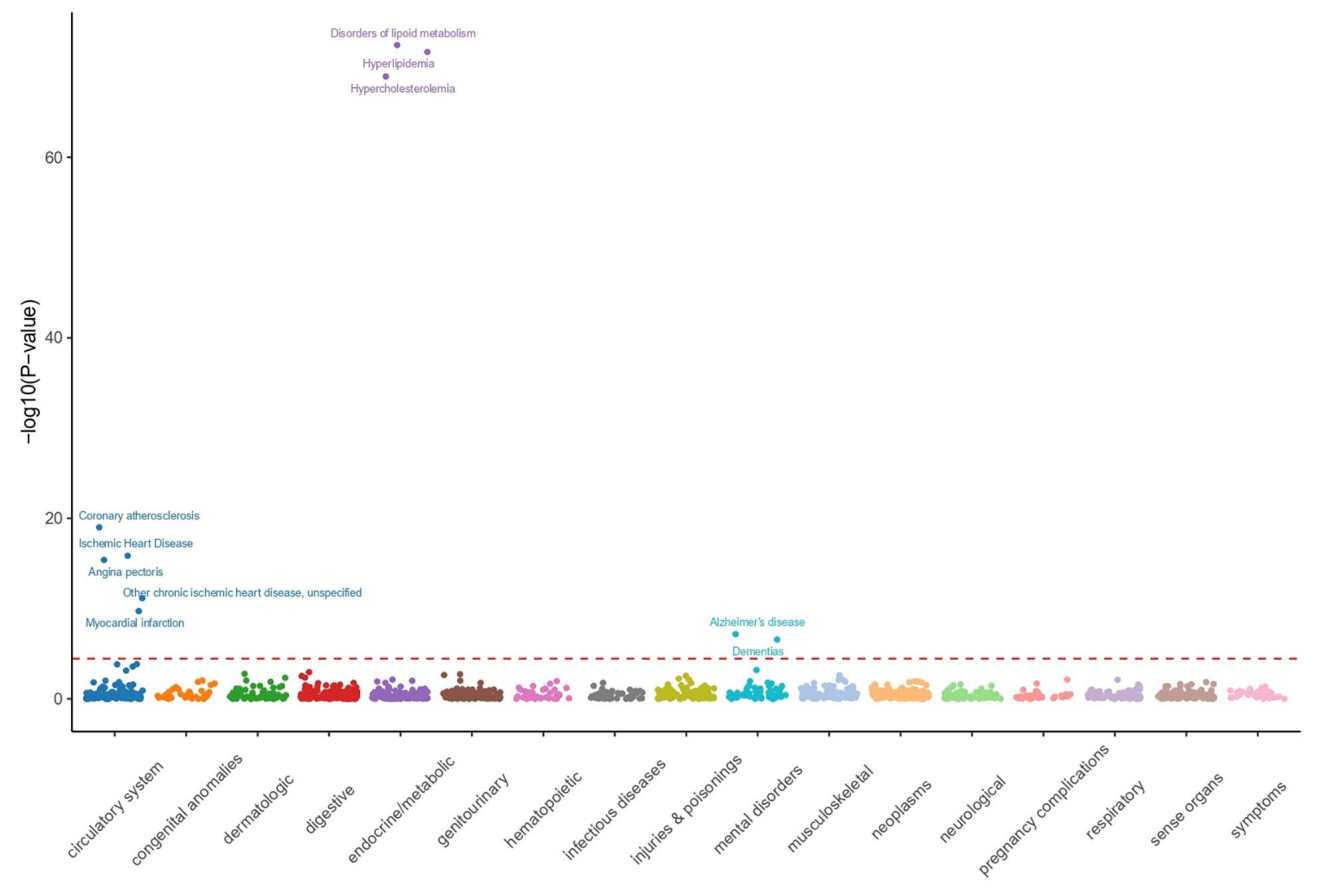
PheWAS analysis of associations between CRC-associated proteins and other disease outcomes

We conducted PheWAS analysis to investigate whether 23 proteins associated with CRC are linked to 1403 diseases and traits in the UK Biobank. Higher genetic levels of plasma VIMP were linked to increased CRC risk, while decreased risks were found for other metabolic diseases, such as disorders of lipid metabolism, hyperlipidemia, hypercholesterolemia, and diseases affecting the circulatory system, including coronary atherosclerosis (Fig. 6). There was a positive association between genetically higher levels of plasma C5orf38 and an increased risk of CRC, as well as of mental disorders such as Alzheimer’s disease, delirium, dementia and amnestic disorders, and other cognitive disorders. In addition, C5orf38 was also found to be associated with the metabolic trait coronary atherosclerosis. Plasma PDE4D exhibited a positive correlation with increased risks of CRC and digestive disorders, specifically Celiac disease and non-celiac intestinal malabsorption. Higher plasma KDR levels were linked to increased risks of CRC, circulatory system diseases, and metabolic disorders, including disorders of lipid metabolism, hypercholesterolemia and hyperlipidemia. Circulatory system diseases linked with higher KDR levels included phlebitis and thrombophlebitis of the lower extremities, phlebitis and thrombophlebitis, pulmonary heart disease, and other disorders of the circulatory system. Plasma MICB levels were found to be genetically linked with higher CRC risk, as well as higher risk of gastrointestinal malabsorption disorders, including celiac disease and non-celiac intestinal malabsorption. In addition, elevated levels of MICB were associated with increased risks of various metabolic disorders, including thyrotoxicosis, type 1 diabetes, and various forms of hypothyroidism, including NOS and Graves’ disease. However, we also observed that lower levels of plasma MICB are associated with lower hematuria risk. Elevated levels of plasma CTF1 were genetically associated with an increased risk of CRC, as well as a stronger predisposition to various mental disorders, including such as Alzheimer’s disease, dementia, delirium, dementia and amnestic disorders, and other cognitive disorders. Furthermore, heightened levels of CTF1 were linked to an increased risk of developing metabolic issues such as hyperlipidemia and disorders of lipid metabolism.

## Discussion

The randomized controlled trials (RCTs) are considered the gold standard for causal inference in epidemiological research due to its unique advantages in minimizing confounding bias [23]. However, strict inclusion and exclusion criteria, medical ethical constraints, and high costs often make it challenging to conduct many RCTs in clinical research. Mendelian randomization is a method used to evaluate potential causal relationships between modifiable exposures (risk factors) and outcomes by utilizing genetic variations linked to the exposure. Its purpose is to mitigate biases arising from confounding factors, including reverse causality, in epidemiological research. More importantly, the two-sample MR is an extension to the one-sample MR design, where estimates for the association of genetic variants with exposure and with outcome are derived from two independent cohorts [24]. Therefore, this study used the two-sample MR design to identify potential therapeutic targets for CRC.

In this study, we conducted two-sample MR analysis to assess the causal relationships between 4489 plasma proteins and CRC. We identified 23 proteins associated with CRC, seven of which showed a protective effect and sixteen of which were associated with increased risk. In addition, we evaluated the causal relationships between plasma proteins and CRC risk factors through two-sample MR analysis, identifying 154 proteins associated with at least one risk factor. Mediation analysis was then performed to assess the indirect effects of plasma proteins on CRC through six risk factors. Four proteins (VIMP, C5orf38, SLC5A8 and MICB) were associated with both CRC outcome and risk factors. Finally, we conducted a phenome-wide association study analysis using data from the UK Biobank to explore the relationships between the 23 CRC-associated proteins and other diseases.

Selenoprotein S (VIMP) is a transmembrane protein localized in the endoplasmic reticulum (ER) and involved in the process of degrading misfolded proteins in the ER, as well as potentially in inflammation control [22]. A study conducted on a Japanese population found that the − 105G > A polymorphism in the SEPS1 promoter was associated with a higher risk of gastric cancer [25]. Meta-analysis revealed a positive correlation between increased VIMP levels and higher risks of CRC and gastric cancer, suggesting that VIMP might be a potential risk factor for both types of cancer [26]. Consistent with this, we found that an increased risk of CRC was associated with higher genetically predicted levels of VIMP (OR [95% CI] = 1.00101 [1.00008, 1.00195]; P = 0.03327; FDR = 0.04429). We also found that genetically higher VIMP levels are associated with a higher risk of cholesterol-to-total-lipids ratio in IDL (OR [95% CI]: 1.58845 [1.34407, 1.87728]; P = 5.66 × 10^− 8^) and PFA (OR [95% CI]: 1.18137 [1.13235, 1.23250], P = 1.27 × 10^− 14^). The study suggests that VIMP may indirectly affect CRC risk via PFA and cholesterol-to-total-lipids ratio in IDL. Future studies on the involvement of VIMP in CRC should investigate the underlying mechanisms by which VIMP affects PFA and cholesterol-to-total-lipids ratio in IDL, enabling the identification of potential therapeutic targets for CRC treatment.

TNF receptor superfamily member 11b (OPG; TNFRSF11B) is a member of the TNF receptor superfamily that acts as a decoy receptor by binding to TRAIL and neutralizing its pro-apoptotic effect [27, 28]. It has been demonstrated to play crucial roles in the onset and progression of various human malignancie [29–33]. Notably, elevated TNFRSF11B expression levels are significantly associated with aggressive invasive behavior in CRC, characterized by increased invasion depth, distant metastasis, and inferior patient prognosis [27, 34, 35]. TNFRSF11B is regulated via the Wnt/β-catenin pathway, contributes to resistance against apoptosis induced by TRAIL, and is found at elevated levels in the serum of patients with advanced colorectal cancers [30]. In this study, we found that increased CRC risk was associated with higher genetically predicted levels of TNFRSF11B (OR [95% CI] = 1.00055 [1.00007, 1.00103]; P = 0.02346; FDR = 0.03597). In conclusion, TNFRSF11B expression is associated with increased CRC risk and may provide crucial growth advantages to cancerous cells, enabling cell invasion and metastasis. Thus, TNFRSF11B inhibition may serve as a promising therapeutic strategy to combat CRC.

Various sensitivity analyses were conducted to ensure that the MR assumptions were not violated and to confirm the reliability of the instrumental variables employed in MR analysis. In the selection of instruments for each plasma protein level, LD clumping was utilized at R^2^ ≤ 0.001 for plasma proteins with P ≤ 5 × 10^− 8^. The IVs used in the MR analysis were examined for heterogeneity, horizontal pleiotropy, and robustness. Heterogeneity was found to be absent (P > 0.05), as was horizontal pleiotropy, and the robustness of the IVs was confirmed through leave-one-out sensitivity testing. In addition, the degree to which VIMP levels affect the cholesterol-to-total-lipids ratio in IDL may be associated with a greater indirect effect of VIMP on CRC through cholesterol-to-total-lipids ratio in IDL in mediation analysis. The validation of this result may necessitate additional data and further experimental confirmation.

We thoroughly investigated the DrugBank database to evaluate the druggability of candidate protein targets [36–40]. Interestingly, we found that several proteins identified in MR analysis are druggable, such as HTATIP2 (Polyethylene glycol 400), CACNA2D3 (Amlodipine and Nilvadipine), SAT2 (Spermine). Notably, there are several KDR-targeting drugs, such as Sorafenib and Sunitinib. Sorafenib is a kinase inhibitor used to treat unresectable liver carcinoma, advanced renal carcinoma, and differentiated thyroid carcinoma. Sunitinib is a receptor tyrosine kinase inhibitor and chemotherapeutic agent used for the treatment of renal cell carcinoma (RCC) and imatinib-resistant gastrointestinal stromal tumor (GIST).

The study has certain limitations. The research sample was limited to individuals of European ancestry, and the external applicability of our findings to other ethnic groups may be constrained by the sample’s composition. To generalize our results to broader populations, additional research with larger and more diverse samples will be necessary.

The findings of our study identify potential targets for future therapeutic interventions in CRC, emphasizing the importance of proteomics in identifying drug targets. Subsequent research is imperative to assess the viability of the 23 identified proteins as potential targets for therapeutic intervention in CRC treatment. Additionally, with the increasing comprehensiveness of proteomics platforms and the expansion of studies on more diverse non-European populations, it is probable that more drug targets for CRC therapy will emerge.

## Conclusions

This study investigated the causal effects of plasma proteins on CRC and its risk factors. Additionally, phenome-wide association study analysis was conducted on data from the UK Biobank to evaluate the associations of 23 plasma proteins with other diseases.

### Electronic supplementary material

Below is the link to the electronic supplementary material.

Supplementary Material 1

## Data Availability

All data used in this study are publicly available data at the summary level, with citations to the relevant studies. GWAS summary statistics for available proteins were obtained from the MR-Base NHGRI-EBI GWAS Catalog (https://gwas.mrcieu.ac.uk/). The summary-level data download links were displayed in Table 1.

## Abbreviations

MR: Mendelian randomization
GWAS: Genome-wide association studies
CRC: Colorectal cancer
SNP: Single nucleotide polymorphism
pQTLs: Protein quantitative trait loci
PheWAS: Phenome-wide association studies
PFA: Polyunsaturated fatty acids
IVW: Inverse variance weighted
OR: Odds ratio
CI: Confidence interval
FDR: False discovery rate.
IVs: Instrument variants
BMI: Body mass index

## References

[1] Sung H, Ferlay J, Siegel RL, Laversanne M, Soerjomataram I, Jemal A, Bray F (2021). Global Cancer statistics 2020: GLOBOCAN estimates of incidence and Mortality Worldwide for 36 cancers in 185 countries. CA Cancer J Clin.

[2] Ferlay J, Soerjomataram I, Dikshit R, Eser S, Mathers C, Rebelo M, Parkin DM, Forman D, Bray F (2015). Cancer incidence and mortality worldwide: sources, methods and major patterns in GLOBOCAN 2012. Int J Cancer.

[3] Suzuki S, Goto A, Nakatochi M, Narita A, Yamaji T, Sawada N, Katagiri R, Iwagami M, Hanyuda A, Hachiya T (2021). Body mass index and Colorectal cancer risk: a mendelian randomization study. Cancer Sci.

[4] Thrift AP, Gong J, Peters U, Chang-Claude J, Rudolph A, Slattery ML, Chan AT, Locke AE, Kahali B, Justice AE (2015). Mendelian randomization study of body Mass Index and Colorectal Cancer Risk. Cancer Epidemiol Biomarkers Prev.

[5] Wang X, Dai JY, Albanes D, Arndt V, Berndt SI, Bezieau S, Brenner H, Buchanan DD, Butterbach K, Caan B (2019). Mendelian randomization analysis of C-reactive protein on Colorectal cancer risk. Int J Epidemiol.

[6] Santos R, Ursu O, Gaulton A, Bento AP, Donadi RS, Bologa CG, Karlsson A, Al-Lazikani B, Hersey A, Oprea TI (2017). A comprehensive map of molecular drug targets. Nat Rev Drug Discov.

[7] Hauser AS, Chavali S, Masuho I, Jahn LJ, Martemyanov KA, Gloriam DE, Babu MM (2018). Pharmacogenomics of GPCR Drug targets. Cell.

[8] Dimou N, Mori N, Harlid S, Harbs J, Martin RM, Smith-Byrne K, Papadimitriou N, Bishop DT, Casey G, Colorado-Yohar SM (2021). Circulating levels of testosterone, sex hormone binding globulin and Colorectal Cancer risk: observational and mendelian randomization analyses. Cancer Epidemiol Biomarkers Prev.

[9] Coghlin C, Murray GI (2013). Progress in the identification of plasma biomarkers of Colorectal cancer. Proteomics.

[10] Sun BB, Maranville JC, Peters JE, Stacey D, Staley JR, Blackshaw J, Burgess S, Jiang T, Paige E, Surendran P (2018). Genomic atlas of the human plasma proteome. Nature.

[11] Ferkingstad E, Sulem P, Atlason BA, Sveinbjornsson G, Magnusson MI, Styrmisdottir EL, Gunnarsdottir K, Helgason A, Oddsson A, Halldorsson BV (2021). Large-scale integration of the plasma proteome with genetics and Disease. Nat Genet.

[12] Folkersen L, Gustafsson S, Wang Q, Hansen DH, Hedman AK, Schork A, Page K, Zhernakova DV, Wu Y, Peters J (2020). Genomic and drug target evaluation of 90 cardiovascular proteins in 30,931 individuals. Nat Metab.

[13] Moncla LM, Mathieu S, Sylla MS, Bosse Y, Theriault S, Arsenault BJ, Mathieu P (2022). Mendelian randomization of circulating proteome identifies actionable targets in Heart Failure. BMC Genomics.

[14] Luo S, Clarke SLN, Ramanan AV, Thompson SD, Langefeld CD, Marion MC, Grom AA, Schooling CM, Gaunt TR, Yeung SLA (2021). Platelet glycoprotein ib alpha-chain as a putative therapeutic target for juvenile idiopathic arthritis: a mendelian randomization study. Arthritis Rheumatol.

[15] Zhao SS, Bovijn J, Hughes DM, Sha T, Zeng C, Lyu H (2022). Genetically predicted vitamin K levels and risk of osteoarthritis: mendelian randomization study. Semin Arthritis Rheum.

[16] Sleiman PM, Grant SF (2010). Mendelian randomization in the era of genomewide association studies. Clin Chem.

[17] Smith GD, Ebrahim S (2003). Mendelian randomization’: can genetic epidemiology contribute to understanding environmental determinants of Disease?. Int J Epidemiol.

[18] Hemani G, Zheng J, Elsworth B, Wade KH, Haberland V, Baird D, Laurin C, Burgess S, Bowden J, Langdon R et al. The MR-Base platform supports systematic causal inference across the human phenome. *eLife* 2018, 7. 10.7554/eLife.34408 PMC5976434 29846171

[19] Zhou W, Nielsen JB, Fritsche LG, Dey R, Gabrielsen ME, Wolford BN, LeFaive J, VandeHaar P, Gagliano SA, Gifford A (2018). Efficiently controlling for case-control imbalance and sample relatedness in large-scale genetic association studies. Nat Genet.

[20] Carter AR, Sanderson E, Hammerton G, Richmond RC, Davey Smith G, Heron J, Taylor AE, Davies NM, Howe LD (2021). Mendelian randomisation for mediation analysis: current methods and challenges for implementation. Eur J Epidemiol.

[21] Zheng J, Haberland V, Baird D, Walker V, Haycock PC, Hurle MR, Gutteridge A, Erola P, Liu Y, Luo S (2020). Phenome-wide mendelian randomization mapping the influence of the plasma proteome on complex Diseases. Nat Genet.

[22] Bubenik JL, Miniard AC, Driscoll DM (2013). Alternative transcripts and 3’UTR elements govern the incorporation of selenocysteine into selenoprotein S. PLoS ONE.

[23] Hariton E, Locascio JJ (2018). Randomised controlled trials - the gold standard for effectiveness research: study design: randomised controlled trials. BJOG.

[24] Lawlor DA (2016). Commentary: two-sample mendelian randomization: opportunities and challenges. Int J Epidemiol.

[25] Shibata T, Arisawa T, Tahara T, Ohkubo M, Yoshioka D, Maruyama N, Fujita H, Kamiya Y, Nakamura M, Nagasaka M (2009). Selenoprotein S (SEPS1) gene – 105G > A promoter polymorphism influences the susceptibility to gastric cancer in the Japanese population. BMC Gastroenterol.

[26] Li J, Zhu Y, Zhou Y, Jiang H, Chen Z, Lu B, Shen X (2020). The SELS rs34713741 polymorphism is Associated with susceptibility to Colorectal Cancer and gastric Cancer: a Meta-analysis. Genet Test Mol Biomarkers.

[27] Kim HS, Yoon G, Do SI, Kim SJ, Kim YW (2016). Down-regulation of osteoprotegerin expression as a novel biomarker for colorectal carcinoma. Oncotarget.

[28] Takeda K, Smyth MJ, Cretney E, Hayakawa Y, Kayagaki N, Yagita H, Okumura K (2002). Critical role for Tumor necrosis factor-related apoptosis-inducing ligand in immune surveillance against Tumor development. J Exp Med.

[29] Brown JM, Corey E, Lee ZD, True LD, Yun TJ, Tondravi M, Vessella RL (2001). Osteoprotegerin and rank ligand expression in Prostate cancer. Urology.

[30] De Toni EN, Thieme SE, Herbst A, Behrens A, Stieber P, Jung A, Blum H, Goke B, Kolligs FT (2008). OPG is regulated by beta-catenin and mediates resistance to TRAIL-induced apoptosis in colon Cancer. Clin Cancer Res.

[31] Holen I, Cross SS, Neville-Webbe HL, Cross NA, Balasubramanian SP, Croucher PI, Evans CA, Lippitt JM, Coleman RE, Eaton CL (2005). Osteoprotegerin (OPG) expression by Breast cancer cells in vitro and breast tumours in vivo–a role in tumour cell survival?. Breast Cancer Res Treat.

[32] Holen I, Croucher PI, Hamdy FC, Eaton CL (2002). Osteoprotegerin (OPG) is a survival factor for human Prostate cancer cells. Cancer Res.

[33] Naumann U, Wick W, Beschorner R, Meyermann R, Weller M (2004). Expression and functional activity of osteoprotegerin in human malignant gliomas. Acta Neuropathol.

[34] Pettersen I, Bakkelund W, Smedsrod B, Sveinbjornsson B (2005). Osteoprotegerin is expressed in colon carcinoma cells. Anticancer Res.

[35] Tsukamoto S, Ishikawa T, Iida S, Ishiguro M, Mogushi K, Mizushima H, Uetake H, Tanaka H, Sugihara K (2011). Clinical significance of osteoprotegerin expression in human Colorectal cancer. Clin Cancer Res.

[36] Knox C, Law V, Jewison T, Liu P, Ly S, Frolkis A, Pon A, Banco K, Mak C, Neveu V (2011). DrugBank 3.0: a comprehensive resource for ‘omics’ research on Drugs. Nucleic Acids Res.

[37] Law V, Knox C, Djoumbou Y, Jewison T, Guo AC, Liu Y, Maciejewski A, Arndt D, Wilson M, Neveu V (2014). DrugBank 4.0: shedding new light on drug metabolism. Nucleic Acids Res.

[38] Wishart DS, Feunang YD, Guo AC, Lo EJ, Marcu A, Grant JR, Sajed T, Johnson D, Li C, Sayeeda Z (2018). DrugBank 5.0: a major update to the DrugBank database for 2018. Nucleic Acids Res.

[39] Wishart DS, Knox C, Guo AC, Cheng D, Shrivastava S, Tzur D, Gautam B, Hassanali M (2008). DrugBank: a knowledgebase for Drugs, drug actions and drug targets. Nucleic Acids Res.

[40] Wishart DS, Knox C, Guo AC, Shrivastava S, Hassanali M, Stothard P, Chang Z, Woolsey J (2006). DrugBank: a comprehensive resource for in silico drug discovery and exploration. Nucleic Acids Res.

