# Supplementary material for: Proteome-wide analysis reveals potential therapeutic targets for Colorectal cancer: a two-sample mendelian randomization study
Source: BMC Cancer. 2023 Dec 4;23:1188. doi: 10.1186/s12885-023-11669-6 (PMC10696874; doi:10.1186/s12885-023-11669-6)

**Supplementary files**

Supplementary Table S1. Causal effect of plasma proteins on CRC.

Supplementary Table S2. Causal effect of risk factors on CRC.

Supplementary Table S3. Causal effect of plasma proteins on risk factors.

Supplementary Figure S1. Indirect effect of SLC5A8 on CRC through smoking.

Supplementary Figure S2. Indirect effect of MICB on CRC through cholesterol to total lipids ratio in IDL.

**Table S1.** Causal effect of plasma proteins on CRC

| Exposure | Outcome | SNPs | Method | OR | 95% CI | P-value |
| --- | --- | --- | --- | --- | --- | --- |
| AKR1A1 | Colorectal cancer | 3 | Inverse variance weighted | 1.00142 | 1.00068-1.00216 | 0.00015 |
| AKR1A1 | Colorectal cancer | 3 | Weighted median | 1.00136 | 1.00056-1.00216 | 0.00088 |
| AKR1A1 | Colorectal cancer | 3 | Weighted mode | 1.00135 | 1.00054-1.00217 | 0.0834 |
| AKR1A1 | Colorectal cancer | 3 | MR Egger | 1.00104 | 0.99966-1.00242 | 0.37799 |
| BGLAP | Colorectal cancer | 4 | Weighted mode | 1.00304 | 1.0005-1.00558 | 0.1005 |
| BGLAP | Colorectal cancer | 4 | Weighted median | 1.00254 | 1.00048-1.00461 | 0.01582 |
| BGLAP | Colorectal cancer | 4 | MR Egger | 1.00895 | 0.99826-1.01975 | 0.24288 |
| BGLAP | Colorectal cancer | 4 | Inverse variance weighted | 1.00211 | 1.00031-1.00392 | 0.02177 |
| C5orf38 | Colorectal cancer | 4 | Inverse variance weighted | 1.00083 | 1.00014-1.00152 | 0.01877 |
| C5orf38 | Colorectal cancer | 4 | Weighted median | 1.00078 | 1.00002-1.00153 | 0.04343 |
| C5orf38 | Colorectal cancer | 4 | Weighted mode | 1.00077 | 0.99997-1.00158 | 0.15663 |
| C5orf38 | Colorectal cancer | 4 | MR Egger | 1.00019 | 0.99747-1.00291 | 0.90522 |
| CACNA2D3 | Colorectal cancer | 3 | Weighted median | 1.00215 | 1.00002-1.00428 | 0.04765 |
| CACNA2D3 | Colorectal cancer | 3 | Weighted mode | 1.00261 | 0.99986-1.00536 | 0.2036 |
| CACNA2D3 | Colorectal cancer | 3 | MR Egger | 1.00291 | 0.99843-1.00741 | 0.42398 |
| CACNA2D3 | Colorectal cancer | 3 | Inverse variance weighted | 1.00193 | 1.00014-1.00372 | 0.03466 |
| CNTN5 | Colorectal cancer | 3 | Weighted median | 1.00133 | 1.00011-1.00255 | 0.0331 |
| CNTN5 | Colorectal cancer | 3 | Weighted mode | 1.0014 | 0.99992-1.00289 | 0.20522 |
| CNTN5 | Colorectal cancer | 3 | MR Egger | 1.00212 | 0.99581-1.00847 | 0.62972 |
| CNTN5 | Colorectal cancer | 3 | Inverse variance weighted | 1.00123 | 1.00004-1.00241 | 0.04271 |
| CRP | Colorectal cancer | 3 | Inverse variance weighted | 0.9978 | 0.99592-0.99969 | 0.02222 |
| CRP | Colorectal cancer | 3 | Weighted mode | 0.99865 | 0.9958-1.00151 | 0.45351 |
| CRP | Colorectal cancer | 3 | Weighted median | 0.9984 | 0.99603-1.00077 | 0.18607 |
| CRP | Colorectal cancer | 3 | MR Egger | 0.98646 | 0.95332-1.02076 | 0.57759 |
| CTF1 | Colorectal cancer | 3 | Inverse variance weighted | 1.0012 | 1.00037-1.00203 | 0.00446 |
| CTF1 | Colorectal cancer | 3 | Weighted median | 1.0012 | 1.00036-1.00205 | 0.00527 |
| CTF1 | Colorectal cancer | 3 | Weighted mode | 1.00127 | 1.00034-1.00219 | 0.11503 |
| CTF1 | Colorectal cancer | 3 | MR Egger | 1.00114 | 0.99189-1.01047 | 0.84942 |
| ETS2 | Colorectal cancer | 3 | Inverse variance weighted | 0.99688 | 0.99511-0.99866 | 0.00058 |
| ETS2 | Colorectal cancer | 3 | Weighted median | 0.9968 | 0.99468-0.99892 | 0.00314 |
| ETS2 | Colorectal cancer | 3 | Weighted mode | 0.99789 | 0.99557-1.00022 | 0.21807 |
| ETS2 | Colorectal cancer | 3 | MR Egger | 0.99866 | 0.99477-1.00257 | 0.62278 |
| HTATIP2 | Colorectal cancer | 3 | MR Egger | 1.00555 | 1.00108-1.01004 | 0.24808 |
| HTATIP2 | Colorectal cancer | 3 | Weighted median | 1.00238 | 1.00012-1.00463 | 0.03881 |
| HTATIP2 | Colorectal cancer | 3 | Weighted mode | 1.00266 | 1.00032-1.00501 | 0.15566 |
| HTATIP2 | Colorectal cancer | 3 | Inverse variance weighted | 1.00226 | 1.00023-1.0043 | 0.02938 |
| IGDCC4 | Colorectal cancer | 3 | Weighted mode | 1.00164 | 0.99964-1.00365 | 0.24982 |
| IGDCC4 | Colorectal cancer | 3 | Weighted median | 1.00164 | 0.99989-1.0034 | 0.06577 |
| IGDCC4 | Colorectal cancer | 3 | MR Egger | 1.00228 | 0.99796-1.00662 | 0.48968 |
| IGDCC4 | Colorectal cancer | 3 | Inverse variance weighted | 1.00192 | 1.00038-1.00346 | 0.01468 |
| IGF2R | Colorectal cancer | 7 | MR Egger | 1.0004 | 0.99886-1.00194 | 0.63409 |
| IGF2R | Colorectal cancer | 7 | Weighted mode | 1.00082 | 0.99997-1.00168 | 0.10828 |
| IGF2R | Colorectal cancer | 7 | Weighted median | 1.00087 | 1.00002-1.00172 | 0.04406 |
| IGF2R | Colorectal cancer | 7 | Inverse variance weighted | 1.00117 | 1.00025-1.0021 | 0.01262 |
| KDR | Colorectal cancer | 4 | Weighted mode | 1.00059 | 0.99954-1.00165 | 0.35131 |
| KDR | Colorectal cancer | 4 | Weighted median | 1.00069 | 0.99975-1.00163 | 0.15243 |
| KDR | Colorectal cancer | 4 | MR Egger | 1.0004 | 0.99891-1.00189 | 0.65484 |
| KDR | Colorectal cancer | 4 | Inverse variance weighted | 1.00083 | 1.00003-1.00163 | 0.04127 |
| KLK14 | Colorectal cancer | 3 | Weighted median | 0.9991 | 0.9981-1.00009 | 0.0744 |
| KLK14 | Colorectal cancer | 3 | MR Egger | 0.99909 | 0.99691-1.00128 | 0.56622 |
| KLK14 | Colorectal cancer | 3 | Weighted mode | 0.99917 | 0.99817-1.00018 | 0.24841 |
| KLK14 | Colorectal cancer | 3 | Inverse variance weighted | 0.99906 | 0.99814-0.99999 | 0.04647 |
| LRP1B | Colorectal cancer | 3 | MR Egger | 1.00411 | 0.99903-1.00922 | 0.35808 |
| LRP1B | Colorectal cancer | 3 | Weighted median | 1.00244 | 1.00019-1.0047 | 0.03366 |
| LRP1B | Colorectal cancer | 3 | Weighted mode | 1.00255 | 1.00002-1.00508 | 0.1873 |
| LRP1B | Colorectal cancer | 3 | Inverse variance weighted | 1.00199 | 1.00008-1.0039 | 0.04069 |
| MAN1A2 | Colorectal cancer | 3 | Inverse variance weighted | 0.99762 | 0.99583-0.99942 | 0.00943 |
| MAN1A2 | Colorectal cancer | 3 | Weighted median | 0.99788 | 0.99587-0.9999 | 0.03968 |
| MAN1A2 | Colorectal cancer | 3 | Weighted mode | 0.99797 | 0.99554-1.0004 | 0.24327 |
| MAN1A2 | Colorectal cancer | 3 | MR Egger | 0.99902 | 0.99303-1.00506 | 0.80413 |
| MICB | Colorectal cancer | 4 | Weighted mode | 0.99898 | 0.99793-1.00003 | 0.15283 |
| MICB | Colorectal cancer | 4 | MR Egger | 0.99998 | 0.99823-1.00172 | 0.98104 |
| MICB | Colorectal cancer | 4 | Weighted median | 0.99895 | 0.99793-0.99997 | 0.04269 |
| MICB | Colorectal cancer | 4 | Inverse variance weighted | 0.99882 | 0.99785-0.99979 | 0.01764 |
| NELL1 | Colorectal cancer | 3 | Inverse variance weighted | 0.99865 | 0.99765-0.99966 | 0.00884 |
| NELL1 | Colorectal cancer | 3 | Weighted median | 0.99861 | 0.99756-0.99966 | 0.00968 |
| NELL1 | Colorectal cancer | 3 | MR Egger | 0.99809 | 0.99661-0.99958 | 0.24107 |
| NELL1 | Colorectal cancer | 3 | Weighted mode | 0.99839 | 0.9973-0.99948 | 0.10122 |
| PDE4D | Colorectal cancer | 4 | Inverse variance weighted | 0.99817 | 0.99694-0.99939 | 0.00343 |
| PDE4D | Colorectal cancer | 4 | Weighted mode | 0.99828 | 0.99679-0.99978 | 0.10986 |
| PDE4D | Colorectal cancer | 4 | MR Egger | 0.9989 | 0.99595-1.00186 | 0.54119 |
| PDE4D | Colorectal cancer | 4 | Weighted median | 0.99828 | 0.99696-0.9996 | 0.01068 |
| SAT2 | Colorectal cancer | 3 | MR Egger | 1.00064 | 0.9937-1.00762 | 0.8869 |
| SAT2 | Colorectal cancer | 3 | Weighted mode | 1.00162 | 0.9985-1.00476 | 0.41558 |
| SAT2 | Colorectal cancer | 3 | Weighted median | 1.00195 | 0.99944-1.00447 | 0.12711 |
| SAT2 | Colorectal cancer | 3 | Inverse variance weighted | 1.00211 | 1.00011-1.00411 | 0.03868 |
| SLC5A8 | Colorectal cancer | 3 | Inverse variance weighted | 1.00252 | 1.00076-1.00428 | 0.00502 |
| SLC5A8 | Colorectal cancer | 3 | Weighted mode | 1.00222 | 0.99988-1.00456 | 0.2045 |
| SLC5A8 | Colorectal cancer | 3 | MR Egger | 1.00374 | 0.99363-1.01395 | 0.60149 |
| SLC5A8 | Colorectal cancer | 3 | Weighted median | 1.00225 | 1.00023-1.00427 | 0.02888 |
| STX7 | Colorectal cancer | 3 | Inverse variance weighted | 1.00119 | 1.0002-1.00219 | 0.019 |
| STX7 | Colorectal cancer | 3 | Weighted median | 1.00118 | 1.00016-1.0022 | 0.02287 |
| STX7 | Colorectal cancer | 3 | MR Egger | 1.00131 | 0.99922-1.0034 | 0.43522 |
| STX7 | Colorectal cancer | 3 | Weighted mode | 1.00134 | 1.00016-1.00252 | 0.15578 |
| TNFRSF11B | Colorectal cancer | 13 | Inverse variance weighted | 1.00055 | 1.00007-1.00103 | 0.02346 |
| TNFRSF11B | Colorectal cancer | 13 | MR Egger | 1.00064 | 1.00004-1.00124 | 0.0614 |
| TNFRSF11B | Colorectal cancer | 13 | Weighted median | 1.00053 | 0.99989-1.00117 | 0.10629 |
| TNFRSF11B | Colorectal cancer | 13 | Weighted mode | 1.00044 | 0.99964-1.00124 | 0.29929 |
| VIMP | Colorectal cancer | 3 | Weighted median | 1.00097 | 1-1.00193 | 0.0498 |
| VIMP | Colorectal cancer | 3 | Weighted mode | 1.00096 | 0.99998-1.00193 | 0.19498 |
| VIMP | Colorectal cancer | 3 | MR Egger | 1.00052 | 0.99788-1.00316 | 0.76637 |
| VIMP | Colorectal cancer | 3 | Inverse variance weighted | 1.00101 | 1.00008-1.00195 | 0.03327 |

**Table S2.** Causal effect of risk factors on CRC

| Exposure | Outcome | SNPs | Method | OR | 95% CI | P-value |
| --- | --- | --- | --- | --- | --- | --- |
| BMI | Colorectal cancer | 315 | Inverse variance weighted | 1.00275 | 1.00069-1.0048 | 0.00882 |
| BMI | Colorectal cancer | 315 | MR Egger | 1.00239 | 0.99754-1.00726 | 0.3352 |
| BMI | Colorectal cancer | 315 | Weighted median | 1.00178 | 0.99832-1.00525 | 0.31371 |
| BMI | Colorectal cancer | 315 | Weighted mode | 1.00093 | 0.99593-1.00596 | 0.71643 |
| Smoking | Colorectal cancer | 233 | Inverse variance weighted | 1.00472 | 1.00121-1.00824 | 0.00828 |
| Smoking | Colorectal cancer | 233 | MR Egger | 1.00074 | 0.98572-1.01599 | 0.92397 |
| Smoking | Colorectal cancer | 233 | Weighted median | 1.00838 | 1.00351-1.01328 | 0.00073 |
| Smoking | Colorectal cancer | 233 | Weighted mode | 1.01396 | 0.99806-1.03011 | 0.08685 |
| Type 2 diabetes | Colorectal cancer | 55 | Inverse variance weighted | 1.00085 | 0.9999-1.0018 | 0.07829 |
| Type 2 diabetes | Colorectal cancer | 55 | MR Egger | 1.00121 | 0.9991-1.00332 | 0.26502 |
| Type 2 diabetes | Colorectal cancer | 55 | Weighted median | 1.00074 | 0.99917-1.00232 | 0.35484 |
| Type 2 diabetes | Colorectal cancer | 55 | Weighted mode | 1.00058 | 0.99892-1.00225 | 0.49534 |
| Cholesterol to total lipids ratio in IDL | Colorectal cancer | 76 | Inverse variance weighted | 1.00153 | 1.00026-1.0028 | 0.01806 |
| Cholesterol to total lipids ratio in IDL | Colorectal cancer | 76 | MR Egger | 1.00105 | 0.99927-1.00282 | 0.25077 |
| Cholesterol to total lipids ratio in IDL | Colorectal cancer | 76 | Weighted median | 1.00184 | 1.00003-1.00365 | 0.0458 |
| Cholesterol to total lipids ratio in IDL | Colorectal cancer | 76 | Weighted mode | 1.00172 | 1.00019-1.00326 | 0.03058 |
| Polyunsaturated fatty acids | Colorectal cancer | 60 | Inverse variance weighted | 1.00179 | 0.9999-1.00367 | 0.06319 |
| Polyunsaturated fatty acids | Colorectal cancer | 60 | MR Egger | 1.00088 | 0.99735-1.00443 | 0.62742 |
| Polyunsaturated fatty acids | Colorectal cancer | 60 | Weighted median | 1.00255 | 1.00017-1.00493 | 0.03563 |
| Polyunsaturated fatty acids | Colorectal cancer | 60 | Weighted mode | 1.003 | 1.00037-1.00564 | 0.02911 |
| LDL cholesterol | Colorectal cancer | 45 | Inverse variance weighted | 1.00176 | 1.00013-1.00338 | 0.03385 |
| LDL cholesterol | Colorectal cancer | 45 | MR Egger | 1.00122 | 0.99877-1.00367 | 0.33603 |
| LDL cholesterol | Colorectal cancer | 45 | Weighted median | 1.00188 | 0.99972-1.00404 | 0.08820 |
| LDL cholesterol | Colorectal cancer | 45 | Weighted mode | 1.00211 | 1.00000-1.00422 | 0.05631 |

**Table S3.** Causal effect of plasma proteins on risk factors

| id.exposure | Exposure | Outcome | SNPs | Method | OR | 95% CI | P-value |
| --- | --- | --- | --- | --- | --- | --- | --- |
| prot-a-1097 | Fibroblast growth factor 7 | BMI | 3 | Inverse variance weighted | 1.01922 | 1.00479-1.03386 | 0.00889 |
| prot-a-1097 | Fibroblast growth factor 7 | BMI | 3 | MR Egger | 1.00656 | 0.96484-1.05009 | 0.81277 |
| prot-a-1097 | Fibroblast growth factor 7 | BMI | 3 | Weighted median | 1.0174 | 0.99985-1.03526 | 0.05197 |
| prot-a-1097 | Fibroblast growth factor 7 | BMI | 3 | Weighted mode | 1.01342 | 0.99301-1.03424 | 0.32773 |
| prot-a-1397 | Intercellular adhesion molecule 1 | BMI | 3 | Inverse variance weighted | 1.0042 | 1.00034-1.00808 | 0.033 |
| prot-a-1397 | Intercellular adhesion molecule 1 | BMI | 3 | MR Egger | 1.00526 | 0.99378-1.01687 | 0.53527 |
| prot-a-1397 | Intercellular adhesion molecule 1 | BMI | 3 | Weighted median | 1.00408 | 1.00145-1.00672 | 0.00234 |
| prot-a-1397 | Intercellular adhesion molecule 1 | BMI | 3 | Weighted mode | 1.00406 | 1.0014-1.00673 | 0.09591 |
| prot-a-1486 | Interleukin-17 receptor A | BMI | 3 | Inverse variance weighted | 1.00583 | 1.00183-1.00984 | 0.00424 |
| prot-a-1486 | Interleukin-17 receptor A | BMI | 3 | MR Egger | 1.00494 | 0.99818-1.01174 | 0.3884 |
| prot-a-1486 | Interleukin-17 receptor A | BMI | 3 | Weighted median | 1.00568 | 1.00166-1.00972 | 0.00558 |
| prot-a-1486 | Interleukin-17 receptor A | BMI | 3 | Weighted mode | 1.00551 | 1.00134-1.0097 | 0.12235 |
| prot-a-1920 | Neutrophil collagenase | BMI | 4 | Inverse variance weighted | 1.00968 | 1.00557-1.01382 | 0 |
| prot-a-1920 | Neutrophil collagenase | BMI | 4 | MR Egger | 1.00951 | 1.00325-1.01581 | 0.0964 |
| prot-a-1920 | Neutrophil collagenase | BMI | 4 | Weighted median | 1.0098 | 1.00552-1.0141 | 1.00E-05 |
| prot-a-1920 | Neutrophil collagenase | BMI | 4 | Weighted mode | 1.00991 | 1.00545-1.0144 | 0.02226 |
| prot-a-2172 | Peptidyl-glycine alpha-amidating monooxygenase | BMI | 6 | Inverse variance weighted | 1.02224 | 1.01361-1.03094 | 0 |
| prot-a-2172 | Peptidyl-glycine alpha-amidating monooxygenase | BMI | 6 | MR Egger | 1.03296 | 1.01373-1.05256 | 0.02771 |
| prot-a-2172 | Peptidyl-glycine alpha-amidating monooxygenase | BMI | 6 | Weighted median | 1.02383 | 1.01488-1.03287 | 0 |
| prot-a-2172 | Peptidyl-glycine alpha-amidating monooxygenase | BMI | 6 | Weighted mode | 1.02924 | 1.01951-1.03907 | 0.00192 |
| prot-a-2557 | Ribonuclease K6 | BMI | 3 | Inverse variance weighted | 1.00544 | 1.00112-1.00978 | 0.01362 |
| prot-a-2557 | Ribonuclease K6 | BMI | 3 | MR Egger | 1.00173 | 0.99416-1.00936 | 0.73303 |
| prot-a-2557 | Ribonuclease K6 | BMI | 3 | Weighted median | 1.00507 | 1.00076-1.0094 | 0.02113 |
| prot-a-2557 | Ribonuclease K6 | BMI | 3 | Weighted mode | 1.0047 | 0.99987-1.00954 | 0.19671 |
| prot-a-2731 | Sialic acid-binding Ig-like lectin 9 | BMI | 3 | Inverse variance weighted | 1.00518 | 1.00231-1.00805 | 0.00039 |
| prot-a-2731 | Sialic acid-binding Ig-like lectin 9 | BMI | 3 | MR Egger | 1.00477 | 0.99593-1.01369 | 0.48293 |
| prot-a-2731 | Sialic acid-binding Ig-like lectin 9 | BMI | 3 | Weighted median | 1.00515 | 1.00224-1.00808 | 0.00053 |
| prot-a-2731 | Sialic acid-binding Ig-like lectin 9 | BMI | 3 | Weighted mode | 1.00516 | 1.00214-1.0082 | 0.07875 |
| prot-a-2906 | Sushi, von Willebrand factor type A, EGF and pentraxin domain-containing protein 1 | BMI | 3 | Inverse variance weighted | 1.01522 | 1.00619-1.02434 | 0.00093 |
| prot-a-2906 | Sushi, von Willebrand factor type A, EGF and pentraxin domain-containing protein 1 | BMI | 3 | MR Egger | 1.01598 | 1.00231-1.02984 | 0.26172 |
| prot-a-2906 | Sushi, von Willebrand factor type A, EGF and pentraxin domain-containing protein 1 | BMI | 3 | Weighted median | 1.01683 | 1.00691-1.02685 | 0.00085 |
| prot-a-2906 | Sushi, von Willebrand factor type A, EGF and pentraxin domain-containing protein 1 | BMI | 3 | Weighted mode | 1.01764 | 1.00751-1.02787 | 0.0757 |
| prot-a-2978 | Tyrosine-protein kinase receptor Tie-1, soluble | BMI | 4 | Inverse variance weighted | 1.00758 | 1.00161-1.01358 | 0.01273 |
| prot-a-2978 | Tyrosine-protein kinase receptor Tie-1, soluble | BMI | 4 | MR Egger | 1.01349 | 1.00543-1.02162 | 0.08136 |
| prot-a-2978 | Tyrosine-protein kinase receptor Tie-1, soluble | BMI | 4 | Weighted median | 1.0105 | 1.00505-1.01598 | 0.00015 |
| prot-a-2978 | Tyrosine-protein kinase receptor Tie-1, soluble | BMI | 4 | Weighted mode | 1.01031 | 1.00435-1.01632 | 0.04264 |
| prot-a-670 | C-reactive protein | BMI | 3 | Inverse variance weighted | 1.01779 | 1.00263-1.03319 | 0.02126 |
| prot-a-670 | C-reactive protein | BMI | 3 | MR Egger | 1.12796 | 0.8156-1.55994 | 0.59946 |
| prot-a-670 | C-reactive protein | BMI | 3 | Weighted median | 1.01049 | 0.99402-1.02723 | 0.21309 |
| prot-a-670 | C-reactive protein | BMI | 3 | Weighted mode | 1.00998 | 0.99106-1.02927 | 0.4116 |
| prot-a-983 | ERO1-like protein beta | BMI | 3 | Inverse variance weighted | 1.00894 | 1.0002-1.01775 | 0.04494 |
| prot-a-983 | ERO1-like protein beta | BMI | 3 | MR Egger | 1.00357 | 0.9764-1.0315 | 0.84145 |
| prot-a-983 | ERO1-like protein beta | BMI | 3 | Weighted median | 1.00883 | 0.99993-1.01782 | 0.05183 |
| prot-a-983 | ERO1-like protein beta | BMI | 3 | Weighted mode | 1.00637 | 0.99654-1.01629 | 0.33249 |
| prot-b-66 | placental growth factor | BMI | 5 | Inverse variance weighted | 1.0078 | 1.00073-1.01492 | 0.03043 |
| prot-b-66 | placental growth factor | BMI | 5 | MR Egger | 1.01327 | 1.00517-1.02144 | 0.04865 |
| prot-b-66 | placental growth factor | BMI | 5 | Weighted median | 1.01027 | 1.00221-1.0184 | 0.01247 |
| prot-b-66 | placental growth factor | BMI | 5 | Weighted mode | 1.01033 | 1.0014-1.01935 | 0.08594 |
| prot-c-2954_56_2 | MMP-8 | BMI | 3 | Inverse variance weighted | 1.00864 | 1.00307-1.01424 | 0.00233 |
| prot-c-2954_56_2 | MMP-8 | BMI | 3 | MR Egger | 1.01601 | 0.99673-1.03566 | 0.35117 |
| prot-c-2954_56_2 | MMP-8 | BMI | 3 | Weighted median | 1.00921 | 1.00316-1.0153 | 0.00279 |
| prot-c-2954_56_2 | MMP-8 | BMI | 3 | Weighted mode | 1.01021 | 1.00315-1.01732 | 0.10488 |
| prot-a-1178 | Guanylate-binding protein 6 | BMI | 3 | Inverse variance weighted | 0.98465 | 0.97335-0.99608 | 0.00862 |
| prot-a-1178 | Guanylate-binding protein 6 | BMI | 3 | MR Egger | 0.99575 | 0.95616-1.03697 | 0.87066 |
| prot-a-1178 | Guanylate-binding protein 6 | BMI | 3 | Weighted median | 0.98616 | 0.97443-0.99804 | 0.02253 |
| prot-a-1178 | Guanylate-binding protein 6 | BMI | 3 | Weighted mode | 0.98859 | 0.97661-1.00072 | 0.20646 |
| prot-a-1250 | Glycerol-3-phosphate dehydrogenase 1-like protein | BMI | 3 | Inverse variance weighted | 0.9857 | 0.97639-0.9951 | 0.00293 |
| prot-a-1250 | Glycerol-3-phosphate dehydrogenase 1-like protein | BMI | 3 | MR Egger | 0.98334 | 0.94235-1.02612 | 0.58102 |
| prot-a-1250 | Glycerol-3-phosphate dehydrogenase 1-like protein | BMI | 3 | Weighted median | 0.98428 | 0.97416-0.9945 | 0.00265 |
| prot-a-1250 | Glycerol-3-phosphate dehydrogenase 1-like protein | BMI | 3 | Weighted mode | 0.99064 | 0.97818-1.00326 | 0.28252 |
| prot-a-1272 | Growth factor receptor-bound protein 7 | BMI | 3 | Inverse variance weighted | 0.98494 | 0.97203-0.99802 | 0.02421 |
| prot-a-1272 | Growth factor receptor-bound protein 7 | BMI | 3 | MR Egger | 0.97332 | 0.93259-1.01583 | 0.4321 |
| prot-a-1272 | Growth factor receptor-bound protein 7 | BMI | 3 | Weighted median | 0.98644 | 0.97246-1.00061 | 0.06064 |
| prot-a-1272 | Growth factor receptor-bound protein 7 | BMI | 3 | Weighted mode | 0.98747 | 0.97139-1.00382 | 0.27118 |
| prot-a-1443 | Insulin-like growth factor I | BMI | 3 | Inverse variance weighted | 0.96518 | 0.94629-0.98446 | 0.00044 |
| prot-a-1443 | Insulin-like growth factor I | BMI | 3 | MR Egger | 0.98711 | 0.9426-1.03373 | 0.67942 |
| prot-a-1443 | Insulin-like growth factor I | BMI | 3 | Weighted median | 0.96958 | 0.94974-0.98984 | 0.00341 |
| prot-a-1443 | Insulin-like growth factor I | BMI | 3 | Weighted mode | 0.97438 | 0.94922-1.0002 | 0.19122 |
| prot-a-1453 | Insulin growth factor-like family member 3 | BMI | 3 | Inverse variance weighted | 0.98348 | 0.97298-0.9941 | 0.00236 |
| prot-a-1453 | Insulin growth factor-like family member 3 | BMI | 3 | MR Egger | 0.98333 | 0.9486-1.01932 | 0.52766 |
| prot-a-1453 | Insulin growth factor-like family member 3 | BMI | 3 | Weighted median | 0.98356 | 0.97142-0.99586 | 0.00892 |
| prot-a-1453 | Insulin growth factor-like family member 3 | BMI | 3 | Weighted mode | 0.98687 | 0.97247-1.00148 | 0.21995 |
| prot-a-1526 | Interleukin-36 alpha | BMI | 3 | Inverse variance weighted | 0.98818 | 0.97815-0.99831 | 0.02236 |
| prot-a-1526 | Interleukin-36 alpha | BMI | 3 | MR Egger | 0.99941 | 0.97116-1.02848 | 0.97424 |
| prot-a-1526 | Interleukin-36 alpha | BMI | 3 | Weighted median | 0.98876 | 0.97932-0.99829 | 0.02085 |
| prot-a-1526 | Interleukin-36 alpha | BMI | 3 | Weighted mode | 0.99223 | 0.98254-1.00201 | 0.25943 |
| prot-a-1582 | Integrin alpha-5 | BMI | 4 | Inverse variance weighted | 0.98339 | 0.97041-0.99653 | 0.01341 |
| prot-a-1582 | Integrin alpha-5 | BMI | 4 | MR Egger | 0.98097 | 0.9251-1.04023 | 0.58664 |
| prot-a-1582 | Integrin alpha-5 | BMI | 4 | Weighted median | 0.97796 | 0.96291-0.99324 | 0.00485 |
| prot-a-1582 | Integrin alpha-5 | BMI | 4 | Weighted mode | 0.97577 | 0.95241-0.99971 | 0.14154 |
| prot-a-1707 | Epididymal-specific lipocalin-10 | BMI | 3 | Inverse variance weighted | 0.95158 | 0.91134-0.9936 | 0.02438 |
| prot-a-1707 | Epididymal-specific lipocalin-10 | BMI | 3 | MR Egger | 0.95735 | 0.83975-1.09143 | 0.6323 |
| prot-a-1707 | Epididymal-specific lipocalin-10 | BMI | 3 | Weighted median | 0.97298 | 0.95383-0.99251 | 0.00691 |
| prot-a-1707 | Epididymal-specific lipocalin-10 | BMI | 3 | Weighted mode | 0.97944 | 0.96234-0.99685 | 0.147 |
| prot-a-1791 | Leucine-rich repeat serine/threonine-protein kinase 2 | BMI | 4 | Inverse variance weighted | 0.99059 | 0.9816-0.99966 | 0.04196 |
| prot-a-1791 | Leucine-rich repeat serine/threonine-protein kinase 2 | BMI | 4 | MR Egger | 0.97183 | 0.93202-1.01334 | 0.31243 |
| prot-a-1791 | Leucine-rich repeat serine/threonine-protein kinase 2 | BMI | 4 | Weighted median | 0.9911 | 0.98098-1.00133 | 0.08786 |
| prot-a-1791 | Leucine-rich repeat serine/threonine-protein kinase 2 | BMI | 4 | Weighted mode | 0.99183 | 0.98028-1.00351 | 0.26323 |
| prot-a-1823 | Cation-dependent mannose-6-phosphate receptor | BMI | 3 | Inverse variance weighted | 0.97966 | 0.96754-0.99193 | 0.00121 |
| prot-a-1823 | Cation-dependent mannose-6-phosphate receptor | BMI | 3 | MR Egger | 0.9872 | 0.94836-1.02762 | 0.64241 |
| prot-a-1823 | Cation-dependent mannose-6-phosphate receptor | BMI | 3 | Weighted median | 0.98073 | 0.96694-0.99471 | 0.00705 |
| prot-a-1823 | Cation-dependent mannose-6-phosphate receptor | BMI | 3 | Weighted mode | 0.98292 | 0.96664-0.99947 | 0.18053 |
| prot-a-19 | Lysosomal acid phosphatase | BMI | 3 | Inverse variance weighted | 0.98933 | 0.98067-0.99807 | 0.01681 |
| prot-a-19 | Lysosomal acid phosphatase | BMI | 3 | MR Egger | 0.98972 | 0.97447-1.00522 | 0.41667 |
| prot-a-19 | Lysosomal acid phosphatase | BMI | 3 | Weighted median | 0.98824 | 0.97862-0.99795 | 0.01776 |
| prot-a-19 | Lysosomal acid phosphatase | BMI | 3 | Weighted mode | 0.9882 | 0.97811-0.99839 | 0.15137 |
| prot-a-2006 | Neural cell adhesion molecule 1, 120 kDa isoform | BMI | 3 | Inverse variance weighted | 0.98281 | 0.96898-0.99684 | 0.01652 |
| prot-a-2006 | Neural cell adhesion molecule 1, 120 kDa isoform | BMI | 3 | MR Egger | 0.98073 | 0.83672-1.14954 | 0.85 |
| prot-a-2006 | Neural cell adhesion molecule 1, 120 kDa isoform | BMI | 3 | Weighted median | 0.98896 | 0.97617-1.00191 | 0.09441 |
| prot-a-2006 | Neural cell adhesion molecule 1, 120 kDa isoform | BMI | 3 | Weighted mode | 0.98938 | 0.97492-1.00406 | 0.29123 |
| prot-a-205 | Aurora kinase B | BMI | 3 | Inverse variance weighted | 0.9793 | 0.96626-0.99253 | 0.00224 |
| prot-a-205 | Aurora kinase B | BMI | 3 | MR Egger | 0.98028 | 0.94154-1.0206 | 0.51016 |
| prot-a-205 | Aurora kinase B | BMI | 3 | Weighted median | 0.98025 | 0.96479-0.99597 | 0.01396 |
| prot-a-205 | Aurora kinase B | BMI | 3 | Weighted mode | 0.98263 | 0.96386-1.00177 | 0.21697 |
| prot-a-208 | Zinc-alpha-2-glycoprotein | BMI | 4 | Inverse variance weighted | 0.97495 | 0.96327-0.98677 | 4.00E-05 |
| prot-a-208 | Zinc-alpha-2-glycoprotein | BMI | 4 | MR Egger | 0.98868 | 0.96005-1.01816 | 0.52681 |
| prot-a-208 | Zinc-alpha-2-glycoprotein | BMI | 4 | Weighted median | 0.98021 | 0.96564-0.99499 | 0.00887 |
| prot-a-208 | Zinc-alpha-2-glycoprotein | BMI | 4 | Weighted mode | 0.98059 | 0.96249-0.99903 | 0.13126 |
| prot-a-2205 | Protocadherin beta-4 | BMI | 3 | Inverse variance weighted | 0.98667 | 0.97775-0.99566 | 0.00375 |
| prot-a-2205 | Protocadherin beta-4 | BMI | 3 | MR Egger | 0.99085 | 0.9471-1.03663 | 0.75845 |
| prot-a-2205 | Protocadherin beta-4 | BMI | 3 | Weighted median | 0.98604 | 0.97628-0.9959 | 0.00561 |
| prot-a-2205 | Protocadherin beta-4 | BMI | 3 | Weighted mode | 0.99153 | 0.98066-1.00253 | 0.26978 |
| prot-a-2231 | Platelet-derived growth factor receptor-like protein | BMI | 3 | Inverse variance weighted | 0.99416 | 0.98966-0.99867 | 0.0113 |
| prot-a-2231 | Platelet-derived growth factor receptor-like protein | BMI | 3 | MR Egger | 0.99946 | 0.99283-1.00614 | 0.8999 |
| prot-a-2231 | Platelet-derived growth factor receptor-like protein | BMI | 3 | Weighted median | 0.99451 | 0.9912-0.99782 | 0.00116 |
| prot-a-2231 | Platelet-derived growth factor receptor-like protein | BMI | 3 | Weighted mode | 0.99453 | 0.99116-0.99791 | 0.08686 |
| prot-a-2398 | PH and SEC7 domain-containing protein 1 | BMI | 3 | Inverse variance weighted | 0.97349 | 0.95163-0.99585 | 0.0204 |
| prot-a-2398 | PH and SEC7 domain-containing protein 1 | BMI | 3 | MR Egger | 0.92322 | 0.90362-0.94324 | 0.0867 |
| prot-a-2398 | PH and SEC7 domain-containing protein 1 | BMI | 3 | Weighted median | 0.97129 | 0.95954-0.98318 | 0 |
| prot-a-2398 | PH and SEC7 domain-containing protein 1 | BMI | 3 | Weighted mode | 0.9628 | 0.95438-0.9713 | 0.01369 |
| prot-a-2538 | Regulator of G-protein signaling 8 | BMI | 3 | Inverse variance weighted | 0.97819 | 0.96551-0.99102 | 0.00091 |
| prot-a-2538 | Regulator of G-protein signaling 8 | BMI | 3 | MR Egger | 0.98516 | 0.95767-1.01344 | 0.48894 |
| prot-a-2538 | Regulator of G-protein signaling 8 | BMI | 3 | Weighted median | 0.97726 | 0.96252-0.99223 | 0.00301 |
| prot-a-2538 | Regulator of G-protein signaling 8 | BMI | 3 | Weighted mode | 0.97513 | 0.9568-0.99381 | 0.12147 |
| prot-a-2573 | E3 ubiquitin-protein ligase RNF8 | BMI | 3 | Inverse variance weighted | 0.99055 | 0.98134-0.99984 | 0.04631 |
| prot-a-2573 | E3 ubiquitin-protein ligase RNF8 | BMI | 3 | MR Egger | 0.98058 | 0.94256-1.02014 | 0.50912 |
| prot-a-2573 | E3 ubiquitin-protein ligase RNF8 | BMI | 3 | Weighted median | 0.99219 | 0.98226-1.00223 | 0.12709 |
| prot-a-2573 | E3 ubiquitin-protein ligase RNF8 | BMI | 3 | Weighted mode | 0.99225 | 0.98226-1.00235 | 0.27111 |
| prot-a-2730 | Sialic acid-binding Ig-like lectin 8 | BMI | 3 | Inverse variance weighted | 0.98816 | 0.97669-0.99976 | 0.04539 |
| prot-a-2730 | Sialic acid-binding Ig-like lectin 8 | BMI | 3 | MR Egger | 0.99418 | 0.97052-1.01841 | 0.71759 |
| prot-a-2730 | Sialic acid-binding Ig-like lectin 8 | BMI | 3 | Weighted median | 0.98807 | 0.97516-1.00115 | 0.07361 |
| prot-a-2730 | Sialic acid-binding Ig-like lectin 8 | BMI | 3 | Weighted mode | 0.98805 | 0.97409-1.0022 | 0.23944 |
| prot-a-2744 | SLAM family member 7 | BMI | 3 | Inverse variance weighted | 0.98946 | 0.98166-0.99732 | 0.00867 |
| prot-a-2744 | SLAM family member 7 | BMI | 3 | MR Egger | 0.99934 | 0.96692-1.03285 | 0.97506 |
| prot-a-2744 | SLAM family member 7 | BMI | 3 | Weighted median | 0.9893 | 0.98179-0.99687 | 0.00565 |
| prot-a-2744 | SLAM family member 7 | BMI | 3 | Weighted mode | 0.99337 | 0.98505-1.00177 | 0.26148 |
| prot-a-2747 | Urea transporter 2 | BMI | 4 | Inverse variance weighted | 0.99248 | 0.98517-0.99984 | 0.04521 |
| prot-a-2747 | Urea transporter 2 | BMI | 4 | MR Egger | 0.98908 | 0.97713-1.00117 | 0.21853 |
| prot-a-2747 | Urea transporter 2 | BMI | 4 | Weighted median | 0.99298 | 0.98489-1.00115 | 0.09182 |
| prot-a-2747 | Urea transporter 2 | BMI | 4 | Weighted mode | 0.99332 | 0.98536-1.00134 | 0.20102 |
| prot-a-2852 | Alpha-N-acetylgalactosaminide alpha-2,6-sialyltransferase 1 | BMI | 4 | Inverse variance weighted | 0.98579 | 0.97634-0.99534 | 0.00361 |
| prot-a-2852 | Alpha-N-acetylgalactosaminide alpha-2,6-sialyltransferase 1 | BMI | 4 | MR Egger | 0.99349 | 0.9712-1.01629 | 0.62951 |
| prot-a-2852 | Alpha-N-acetylgalactosaminide alpha-2,6-sialyltransferase 1 | BMI | 4 | Weighted median | 0.9887 | 0.97777-0.99975 | 0.0451 |
| prot-a-2852 | Alpha-N-acetylgalactosaminide alpha-2,6-sialyltransferase 1 | BMI | 4 | Weighted mode | 0.98959 | 0.97689-1.00245 | 0.21042 |
| prot-a-2904 | Histone-lysine N-methyltransferase SUV420H2 | BMI | 3 | Inverse variance weighted | 0.97784 | 0.96323-0.99266 | 0.00351 |
| prot-a-2904 | Histone-lysine N-methyltransferase SUV420H2 | BMI | 3 | MR Egger | 0.9814 | 0.94177-1.0227 | 0.53606 |
| prot-a-2904 | Histone-lysine N-methyltransferase SUV420H2 | BMI | 3 | Weighted median | 0.98009 | 0.96298-0.99751 | 0.02524 |
| prot-a-2904 | Histone-lysine N-methyltransferase SUV420H2 | BMI | 3 | Weighted mode | 0.98123 | 0.95975-1.0032 | 0.23555 |
| prot-a-2991 | Toll-like receptor 4:Lymphocyte antigen 96 complex | BMI | 3 | Inverse variance weighted | 0.98996 | 0.98346-0.9965 | 0.00268 |
| prot-a-2991 | Toll-like receptor 4:Lymphocyte antigen 96 complex | BMI | 3 | MR Egger | 0.98775 | 0.9696-1.00623 | 0.41666 |
| prot-a-2991 | Toll-like receptor 4:Lymphocyte antigen 96 complex | BMI | 3 | Weighted median | 0.99082 | 0.98348-0.99821 | 0.01505 |
| prot-a-2991 | Toll-like receptor 4:Lymphocyte antigen 96 complex | BMI | 3 | Weighted mode | 0.99113 | 0.98327-0.99904 | 0.15927 |
| prot-a-3076 | Torsin-1A-interacting protein 1 | BMI | 4 | Inverse variance weighted | 0.99294 | 0.98606-0.99988 | 0.0461 |
| prot-a-3076 | Torsin-1A-interacting protein 1 | BMI | 4 | MR Egger | 0.99738 | 0.97649-1.01871 | 0.83037 |
| prot-a-3076 | Torsin-1A-interacting protein 1 | BMI | 4 | Weighted median | 0.99365 | 0.98629-1.00106 | 0.09303 |
| prot-a-3076 | Torsin-1A-interacting protein 1 | BMI | 4 | Weighted mode | 0.99381 | 0.98493-1.00278 | 0.26869 |
| prot-a-310 | Augurin | BMI | 3 | Inverse variance weighted | 0.97705 | 0.9638-0.99049 | 0.00087 |
| prot-a-310 | Augurin | BMI | 3 | MR Egger | 0.97766 | 0.93695-1.02013 | 0.4871 |
| prot-a-310 | Augurin | BMI | 3 | Weighted median | 0.97694 | 0.96137-0.99276 | 0.00441 |
| prot-a-310 | Augurin | BMI | 3 | Weighted mode | 0.97691 | 0.95895-0.99522 | 0.13252 |
| prot-a-316 | Protein CEI | BMI | 3 | Inverse variance weighted | 0.96852 | 0.95935-0.97777 | 0 |
| prot-a-316 | Protein CEI | BMI | 3 | MR Egger | 0.94159 | 0.88231-1.00486 | 0.32077 |
| prot-a-316 | Protein CEI | BMI | 3 | Weighted median | 0.96878 | 0.95834-0.97933 | 0 |
| prot-a-316 | Protein CEI | BMI | 3 | Weighted mode | 0.96687 | 0.95507-0.97882 | 0.03291 |
| prot-a-3254 | Tyrosine-protein kinase ZAP-70 | BMI | 3 | Inverse variance weighted | 0.98082 | 0.96791-0.99391 | 0.0042 |
| prot-a-3254 | Tyrosine-protein kinase ZAP-70 | BMI | 3 | MR Egger | 0.98636 | 0.9609-1.0125 | 0.49088 |
| prot-a-3254 | Tyrosine-protein kinase ZAP-70 | BMI | 3 | Weighted median | 0.98408 | 0.96851-0.99989 | 0.04848 |
| prot-a-3254 | Tyrosine-protein kinase ZAP-70 | BMI | 3 | Weighted mode | 0.9848 | 0.96767-1.00224 | 0.22933 |
| prot-a-347 | Calcium/calmodulin-dependent protein kinase type 1D | BMI | 3 | Inverse variance weighted | 0.98626 | 0.97617-0.99645 | 0.00832 |
| prot-a-347 | Calcium/calmodulin-dependent protein kinase type 1D | BMI | 3 | MR Egger | 0.99778 | 0.95413-1.04343 | 0.93814 |
| prot-a-347 | Calcium/calmodulin-dependent protein kinase type 1D | BMI | 3 | Weighted median | 0.98664 | 0.97649-0.99689 | 0.01072 |
| prot-a-347 | Calcium/calmodulin-dependent protein kinase type 1D | BMI | 3 | Weighted mode | 0.99099 | 0.98021-1.0019 | 0.24667 |
| prot-a-398 | C-C motif chemokine 22 | BMI | 3 | Inverse variance weighted | 0.97788 | 0.96015-0.99595 | 0.01662 |
| prot-a-398 | C-C motif chemokine 22 | BMI | 3 | MR Egger | 0.94918 | 0.92084-0.97839 | 0.18352 |
| prot-a-398 | C-C motif chemokine 22 | BMI | 3 | Weighted median | 0.98041 | 0.96599-0.99505 | 0.0089 |
| prot-a-398 | C-C motif chemokine 22 | BMI | 3 | Weighted mode | 0.9676 | 0.94431-0.99147 | 0.11784 |
| prot-a-444 | ADP-ribosyl cyclase/cyclic ADP-ribose hydrolase 1 | BMI | 3 | Inverse variance weighted | 0.97675 | 0.95772-0.99616 | 0.01913 |
| prot-a-444 | ADP-ribosyl cyclase/cyclic ADP-ribose hydrolase 1 | BMI | 3 | MR Egger | 0.94863 | 0.9018-0.9979 | 0.28997 |
| prot-a-444 | ADP-ribosyl cyclase/cyclic ADP-ribose hydrolase 1 | BMI | 3 | Weighted median | 0.98354 | 0.96389-1.00358 | 0.10687 |
| prot-a-444 | ADP-ribosyl cyclase/cyclic ADP-ribose hydrolase 1 | BMI | 3 | Weighted mode | 0.98625 | 0.96449-1.0085 | 0.34799 |
| prot-a-467 | T-cell surface glycoprotein CD8 beta chain | BMI | 3 | Inverse variance weighted | 0.97914 | 0.96415-0.99436 | 0.00738 |
| prot-a-467 | T-cell surface glycoprotein CD8 beta chain | BMI | 3 | MR Egger | 0.99256 | 0.95683-1.02962 | 0.75822 |
| prot-a-467 | T-cell surface glycoprotein CD8 beta chain | BMI | 3 | Weighted median | 0.97936 | 0.96063-0.99846 | 0.0343 |
| prot-a-467 | T-cell surface glycoprotein CD8 beta chain | BMI | 3 | Weighted mode | 0.97869 | 0.95576-1.00218 | 0.21706 |
| prot-a-564 | Cytoskeleton-associated protein 2 | BMI | 5 | Inverse variance weighted | 0.98291 | 0.97196-0.99398 | 0.00256 |
| prot-a-564 | Cytoskeleton-associated protein 2 | BMI | 5 | MR Egger | 0.98332 | 0.93888-1.02987 | 0.52753 |
| prot-a-564 | Cytoskeleton-associated protein 2 | BMI | 5 | Weighted median | 0.98046 | 0.96637-0.99476 | 0.00757 |
| prot-a-564 | Cytoskeleton-associated protein 2 | BMI | 5 | Weighted mode | 0.97883 | 0.9604-0.99762 | 0.09206 |
| prot-a-607 | Contactin-2 | BMI | 4 | Inverse variance weighted | 0.98176 | 0.96859-0.99511 | 0.00757 |
| prot-a-607 | Contactin-2 | BMI | 4 | MR Egger | 0.98165 | 0.94577-1.0189 | 0.43254 |
| prot-a-607 | Contactin-2 | BMI | 4 | Weighted median | 0.97914 | 0.97177-0.98656 | 0 |
| prot-a-607 | Contactin-2 | BMI | 4 | Weighted mode | 0.97838 | 0.9715-0.98532 | 0.009 |
| prot-a-612 | Cytochrome c oxidase assembly factor 3 homolog, mitochondrial | BMI | 3 | Inverse variance weighted | 0.97976 | 0.96649-0.99322 | 0.00331 |
| prot-a-612 | Cytochrome c oxidase assembly factor 3 homolog, mitochondrial | BMI | 3 | MR Egger | 0.98234 | 0.9513-1.0144 | 0.47342 |
| prot-a-612 | Cytochrome c oxidase assembly factor 3 homolog, mitochondrial | BMI | 3 | Weighted median | 0.9812 | 0.96459-0.99809 | 0.02927 |
| prot-a-612 | Cytochrome c oxidase assembly factor 3 homolog, mitochondrial | BMI | 3 | Weighted mode | 0.98237 | 0.96374-1.00135 | 0.21011 |
| prot-a-710 | Cardiotrophin-1 | BMI | 3 | Inverse variance weighted | 0.97568 | 0.95632-0.99543 | 0.01606 |
| prot-a-710 | Cardiotrophin-1 | BMI | 3 | MR Egger | 0.9662 | 0.69953-1.33453 | 0.86903 |
| prot-a-710 | Cardiotrophin-1 | BMI | 3 | Weighted median | 0.97767 | 0.97115-0.98423 | 0 |
| prot-a-710 | Cardiotrophin-1 | BMI | 3 | Weighted mode | 0.96796 | 0.96134-0.97463 | 0.01137 |
| prot-a-970 | Epiphycan | BMI | 3 | Inverse variance weighted | 0.971 | 0.94547-0.99722 | 0.0304 |
| prot-a-970 | Epiphycan | BMI | 3 | MR Egger | 0.94975 | 0.88626-1.01778 | 0.38218 |
| prot-a-970 | Epiphycan | BMI | 3 | Weighted median | 0.9696 | 0.95239-0.98712 | 0.00073 |
| prot-a-970 | Epiphycan | BMI | 3 | Weighted mode | 0.9575 | 0.92911-0.98677 | 0.10564 |
| prot-a-982 | ERO1-like protein alpha | BMI | 3 | Inverse variance weighted | 0.98421 | 0.97359-0.99494 | 0.00402 |
| prot-a-982 | ERO1-like protein alpha | BMI | 3 | MR Egger | 0.99133 | 0.95298-1.03124 | 0.74026 |
| prot-a-982 | ERO1-like protein alpha | BMI | 3 | Weighted median | 0.98537 | 0.97382-0.99705 | 0.01424 |
| prot-a-982 | ERO1-like protein alpha | BMI | 3 | Weighted mode | 0.98792 | 0.97483-1.0012 | 0.2163 |
| prot-b-25 | matrix metallopeptidase 3 | BMI | 4 | Inverse variance weighted | 0.99698 | 0.99494-0.99901 | 0.00366 |
| prot-b-25 | matrix metallopeptidase 3 | BMI | 4 | MR Egger | 0.99742 | 0.99513-0.99972 | 0.15903 |
| prot-b-25 | matrix metallopeptidase 3 | BMI | 4 | Weighted median | 0.99713 | 0.99474-0.99953 | 0.01911 |
| prot-b-25 | matrix metallopeptidase 3 | BMI | 4 | Weighted mode | 0.99721 | 0.99485-0.99958 | 0.10409 |
| prot-a-1046 | Glycosaminoglycan xylosylkinase | Type 2 diabetes | 3 | Inverse variance weighted | 1.17148 | 1.05381-1.3023 | 0.00338 |
| prot-a-1046 | Glycosaminoglycan xylosylkinase | Type 2 diabetes | 3 | MR Egger | 1.51209 | 1.12251-2.03686 | 0.22426 |
| prot-a-1046 | Glycosaminoglycan xylosylkinase | Type 2 diabetes | 3 | Weighted median | 1.16265 | 1.05639-1.2796 | 0.00206 |
| prot-a-1046 | Glycosaminoglycan xylosylkinase | Type 2 diabetes | 3 | Weighted mode | 1.21029 | 1.09898-1.33287 | 0.06054 |
| prot-a-1051 | Protein FAM3D | Type 2 diabetes | 3 | Inverse variance weighted | 1.04288 | 1.00955-1.0773 | 0.01128 |
| prot-a-1051 | Protein FAM3D | Type 2 diabetes | 3 | MR Egger | 1.17016 | 1.02106-1.34102 | 0.26523 |
| prot-a-1051 | Protein FAM3D | Type 2 diabetes | 3 | Weighted median | 1.04692 | 1.01841-1.07623 | 0.00113 |
| prot-a-1051 | Protein FAM3D | Type 2 diabetes | 3 | Weighted mode | 1.05095 | 1.02163-1.08112 | 0.07504 |
| prot-a-1347 | HLA class II histocompatibility antigen, DQ alpha 2 chain | Type 2 diabetes | 4 | Inverse variance weighted | 1.05438 | 1.00702-1.10397 | 0.02393 |
| prot-a-1347 | HLA class II histocompatibility antigen, DQ alpha 2 chain | Type 2 diabetes | 4 | MR Egger | 1.10271 | 0.99898-1.21722 | 0.19197 |
| prot-a-1347 | HLA class II histocompatibility antigen, DQ alpha 2 chain | Type 2 diabetes | 4 | Weighted median | 1.06061 | 1.00909-1.11477 | 0.02056 |
| prot-a-1347 | HLA class II histocompatibility antigen, DQ alpha 2 chain | Type 2 diabetes | 4 | Weighted mode | 1.0701 | 1.01193-1.13161 | 0.09797 |
| prot-a-2557 | Ribonuclease K6 | Type 2 diabetes | 3 | Inverse variance weighted | 1.02653 | 1.00245-1.05119 | 0.03059 |
| prot-a-2557 | Ribonuclease K6 | Type 2 diabetes | 3 | MR Egger | 1.0044 | 0.96345-1.04709 | 0.87025 |
| prot-a-2557 | Ribonuclease K6 | Type 2 diabetes | 3 | Weighted median | 1.02285 | 0.99852-1.04777 | 0.0659 |
| prot-a-2557 | Ribonuclease K6 | Type 2 diabetes | 3 | Weighted mode | 1.02182 | 0.99529-1.04906 | 0.24898 |
| prot-a-2906 | Sushi, von Willebrand factor type A, EGF and pentraxin domain-containing protein 1 | Type 2 diabetes | 4 | Inverse variance weighted | 1.08159 | 1.02484-1.14147 | 0.00434 |
| prot-a-2906 | Sushi, von Willebrand factor type A, EGF and pentraxin domain-containing protein 1 | Type 2 diabetes | 4 | MR Egger | 1.10282 | 1.00267-1.21299 | 0.1815 |
| prot-a-2906 | Sushi, von Willebrand factor type A, EGF and pentraxin domain-containing protein 1 | Type 2 diabetes | 4 | Weighted median | 1.08241 | 1.02955-1.13799 | 0.00194 |
| prot-a-2906 | Sushi, von Willebrand factor type A, EGF and pentraxin domain-containing protein 1 | Type 2 diabetes | 4 | Weighted mode | 1.08523 | 1.03137-1.1419 | 0.0513 |
| prot-a-2907 | Sushi, von Willebrand factor type A, EGF and pentraxin domain-containing protein 1 | Type 2 diabetes | 4 | Inverse variance weighted | 1.06603 | 1.02518-1.10852 | 0.00134 |
| prot-a-2907 | Sushi, von Willebrand factor type A, EGF and pentraxin domain-containing protein 1 | Type 2 diabetes | 4 | MR Egger | 1.07171 | 1.00562-1.14215 | 0.16658 |
| prot-a-2907 | Sushi, von Willebrand factor type A, EGF and pentraxin domain-containing protein 1 | Type 2 diabetes | 4 | Weighted median | 1.07248 | 1.02778-1.11912 | 0.00128 |
| prot-a-2907 | Sushi, von Willebrand factor type A, EGF and pentraxin domain-containing protein 1 | Type 2 diabetes | 4 | Weighted mode | 1.07839 | 1.03236-1.12647 | 0.04274 |
| prot-a-3093 | Protein-tyrosine sulfotransferase 2 | Type 2 diabetes | 4 | Inverse variance weighted | 1.11948 | 1.02502-1.22263 | 0.01209 |
| prot-a-3093 | Protein-tyrosine sulfotransferase 2 | Type 2 diabetes | 4 | MR Egger | 1.27879 | 0.74506-2.19489 | 0.46641 |
| prot-a-3093 | Protein-tyrosine sulfotransferase 2 | Type 2 diabetes | 4 | Weighted median | 1.13663 | 1.06715-1.21063 | 7.00E-05 |
| prot-a-3093 | Protein-tyrosine sulfotransferase 2 | Type 2 diabetes | 4 | Weighted mode | 1.05404 | 0.92698-1.19852 | 0.48068 |
| prot-a-426 | CD209 antigen | Type 2 diabetes | 3 | Inverse variance weighted | 1.0562 | 1.03169-1.0813 | 1.00E-05 |
| prot-a-426 | CD209 antigen | Type 2 diabetes | 3 | MR Egger | 1.07172 | 1.01851-1.12771 | 0.22845 |
| prot-a-426 | CD209 antigen | Type 2 diabetes | 3 | Weighted median | 1.05791 | 1.03316-1.08324 | 0 |
| prot-a-426 | CD209 antigen | Type 2 diabetes | 3 | Weighted mode | 1.05772 | 1.03241-1.08365 | 0.04523 |
| prot-a-543 | Chitotriosidase-1 | Type 2 diabetes | 3 | Inverse variance weighted | 1.04057 | 1.01526-1.0665 | 0.00155 |
| prot-a-543 | Chitotriosidase-1 | Type 2 diabetes | 3 | MR Egger | 1.04361 | 1.00691-1.08165 | 0.25738 |
| prot-a-543 | Chitotriosidase-1 | Type 2 diabetes | 3 | Weighted median | 1.0412 | 1.01636-1.06665 | 0.00105 |
| prot-a-543 | Chitotriosidase-1 | Type 2 diabetes | 3 | Weighted mode | 1.04123 | 1.01512-1.06801 | 0.0893 |
| prot-a-1622 | Vascular endothelial growth factor receptor 2 | Type 2 diabetes | 4 | Inverse variance weighted | 0.94594 | 0.9016-0.99246 | 0.02326 |
| prot-a-1622 | Vascular endothelial growth factor receptor 2 | Type 2 diabetes | 4 | MR Egger | 0.96073 | 0.86369-1.06867 | 0.53764 |
| prot-a-1622 | Vascular endothelial growth factor receptor 2 | Type 2 diabetes | 4 | Weighted median | 0.94888 | 0.90698-0.99271 | 0.02276 |
| prot-a-1622 | Vascular endothelial growth factor receptor 2 | Type 2 diabetes | 4 | Weighted mode | 0.98565 | 0.89633-1.08389 | 0.78508 |
| prot-a-1879 | Protein MENT | Type 2 diabetes | 3 | Inverse variance weighted | 0.88178 | 0.78567-0.98965 | 0.03262 |
| prot-a-1879 | Protein MENT | Type 2 diabetes | 3 | MR Egger | 0.71595 | 0.58859-0.87085 | 0.185 |
| prot-a-1879 | Protein MENT | Type 2 diabetes | 3 | Weighted median | 0.89 | 0.82785-0.95681 | 0.0016 |
| prot-a-1879 | Protein MENT | Type 2 diabetes | 3 | Weighted mode | 0.8338 | 0.77419-0.898 | 0.04073 |
| prot-a-2991 | Toll-like receptor 4:Lymphocyte antigen 96 complex | Type 2 diabetes | 4 | Inverse variance weighted | 0.92593 | 0.87031-0.98511 | 0.0149 |
| prot-a-2991 | Toll-like receptor 4:Lymphocyte antigen 96 complex | Type 2 diabetes | 4 | MR Egger | 0.98948 | 0.91063-1.07516 | 0.82616 |
| prot-a-2991 | Toll-like receptor 4:Lymphocyte antigen 96 complex | Type 2 diabetes | 4 | Weighted median | 0.9321 | 0.89548-0.97022 | 0.00058 |
| prot-a-2991 | Toll-like receptor 4:Lymphocyte antigen 96 complex | Type 2 diabetes | 4 | Weighted mode | 0.95572 | 0.9183-0.99467 | 0.11283 |
| prot-a-316 | Protein CEI | Type 2 diabetes | 3 | Inverse variance weighted | 0.87768 | 0.78834-0.97715 | 0.01722 |
| prot-a-316 | Protein CEI | Type 2 diabetes | 3 | MR Egger | 0.82488 | 0.27495-2.47474 | 0.78939 |
| prot-a-316 | Protein CEI | Type 2 diabetes | 3 | Weighted median | 0.88837 | 0.83645-0.94351 | 0.00012 |
| prot-a-316 | Protein CEI | Type 2 diabetes | 3 | Weighted mode | 0.89062 | 0.83887-0.94556 | 0.06301 |
| prot-c-2977_7_2 | EDAR | Type 2 diabetes | 3 | Inverse variance weighted | 0.9442 | 0.90592-0.98411 | 0.00656 |
| prot-c-2977_7_2 | EDAR | Type 2 diabetes | 3 | MR Egger | 0.91673 | 0.74825-1.12316 | 0.55557 |
| prot-c-2977_7_2 | EDAR | Type 2 diabetes | 3 | Weighted median | 0.93589 | 0.89103-0.983 | 0.00819 |
| prot-c-2977_7_2 | EDAR | Type 2 diabetes | 3 | Weighted mode | 0.9329 | 0.88046-0.98846 | 0.14287 |
| prot-a-1046 | Glycosaminoglycan xylosylkinase | Cholesterol to total lipids ratio in IDL | 3 | Inverse variance weighted | 1.0926 | 1.01361-1.17775 | 0.02072 |
| prot-a-1046 | Glycosaminoglycan xylosylkinase | Cholesterol to total lipids ratio in IDL | 3 | MR Egger | 1.34266 | 1.20104-1.50098 | 0.12138 |
| prot-a-1046 | Glycosaminoglycan xylosylkinase | Cholesterol to total lipids ratio in IDL | 3 | Weighted median | 1.05257 | 1.00506-1.10232 | 0.02968 |
| prot-a-1046 | Glycosaminoglycan xylosylkinase | Cholesterol to total lipids ratio in IDL | 3 | Weighted mode | 1.03438 | 0.98201-1.08954 | 0.33039 |
| prot-a-1051 | Protein FAM3D | Cholesterol to total lipids ratio in IDL | 3 | Inverse variance weighted | 1.02851 | 1.00569-1.05186 | 0.01408 |
| prot-a-1051 | Protein FAM3D | Cholesterol to total lipids ratio in IDL | 3 | MR Egger | 1.1256 | 1.068-1.1863 | 0.1418 |
| prot-a-1051 | Protein FAM3D | Cholesterol to total lipids ratio in IDL | 3 | Weighted median | 1.01894 | 1.00616-1.03189 | 0.00358 |
| prot-a-1051 | Protein FAM3D | Cholesterol to total lipids ratio in IDL | 3 | Weighted mode | 1.04427 | 1.02894-1.05984 | 0.02905 |
| prot-a-1277 | Granulins | Cholesterol to total lipids ratio in IDL | 4 | Inverse variance weighted | 1.02631 | 1.00003-1.05329 | 0.04974 |
| prot-a-1277 | Granulins | Cholesterol to total lipids ratio in IDL | 4 | MR Egger | 1.06071 | 1.03035-1.09196 | 0.05776 |
| prot-a-1277 | Granulins | Cholesterol to total lipids ratio in IDL | 4 | Weighted median | 1.02994 | 1.01797-1.04206 | 0 |
| prot-a-1277 | Granulins | Cholesterol to total lipids ratio in IDL | 4 | Weighted mode | 1.03399 | 1.02164-1.04649 | 0.01213 |
| prot-a-1305 | GDH/6PGL endoplasmic bifunctional protein | Cholesterol to total lipids ratio in IDL | 5 | Inverse variance weighted | 1.01977 | 1.00766-1.03202 | 0.00132 |
| prot-a-1305 | GDH/6PGL endoplasmic bifunctional protein | Cholesterol to total lipids ratio in IDL | 5 | MR Egger | 1.01657 | 0.99036-1.04347 | 0.30537 |
| prot-a-1305 | GDH/6PGL endoplasmic bifunctional protein | Cholesterol to total lipids ratio in IDL | 5 | Weighted median | 1.0234 | 1.01025-1.03672 | 0.00046 |
| prot-a-1305 | GDH/6PGL endoplasmic bifunctional protein | Cholesterol to total lipids ratio in IDL | 5 | Weighted mode | 1.02736 | 1.01254-1.04239 | 0.02193 |
| prot-a-134 | Apolipoprotein L1 | Cholesterol to total lipids ratio in IDL | 4 | Inverse variance weighted | 1.02951 | 1.00852-1.05093 | 0.00565 |
| prot-a-134 | Apolipoprotein L1 | Cholesterol to total lipids ratio in IDL | 4 | MR Egger | 1.04451 | 0.97071-1.12392 | 0.36423 |
| prot-a-134 | Apolipoprotein L1 | Cholesterol to total lipids ratio in IDL | 4 | Weighted median | 1.03524 | 1.01888-1.05186 | 2.00E-05 |
| prot-a-134 | Apolipoprotein L1 | Cholesterol to total lipids ratio in IDL | 4 | Weighted mode | 1.0368 | 1.01943-1.05446 | 0.02474 |
| prot-a-1458 | Immunoglobulin lambda-like polypeptide 1 | Cholesterol to total lipids ratio in IDL | 7 | Inverse variance weighted | 1.02283 | 1.00069-1.04545 | 0.04321 |
| prot-a-1458 | Immunoglobulin lambda-like polypeptide 1 | Cholesterol to total lipids ratio in IDL | 7 | MR Egger | 1.00739 | 0.96386-1.05288 | 0.75726 |
| prot-a-1458 | Immunoglobulin lambda-like polypeptide 1 | Cholesterol to total lipids ratio in IDL | 7 | Weighted median | 1.02236 | 0.9987-1.04659 | 0.06416 |
| prot-a-1458 | Immunoglobulin lambda-like polypeptide 1 | Cholesterol to total lipids ratio in IDL | 7 | Weighted mode | 1.02015 | 0.99059-1.0506 | 0.2319 |
| prot-a-1753 | Lipase member N | Cholesterol to total lipids ratio in IDL | 4 | Inverse variance weighted | 1.01055 | 1.00232-1.01884 | 0.0119 |
| prot-a-1753 | Lipase member N | Cholesterol to total lipids ratio in IDL | 4 | MR Egger | 1.01917 | 0.99502-1.0439 | 0.26092 |
| prot-a-1753 | Lipase member N | Cholesterol to total lipids ratio in IDL | 4 | Weighted median | 1.01119 | 1.00248-1.01998 | 0.01175 |
| prot-a-1753 | Lipase member N | Cholesterol to total lipids ratio in IDL | 4 | Weighted mode | 1.01158 | 1.0036-1.01963 | 0.06524 |
| prot-a-2129 | ADP-ribose pyrophosphatase, mitochondrial | Cholesterol to total lipids ratio in IDL | 3 | Inverse variance weighted | 1.027 | 1.00935-1.04496 | 0.0026 |
| prot-a-2129 | ADP-ribose pyrophosphatase, mitochondrial | Cholesterol to total lipids ratio in IDL | 3 | MR Egger | 1.03052 | 0.99201-1.07052 | 0.36529 |
| prot-a-2129 | ADP-ribose pyrophosphatase, mitochondrial | Cholesterol to total lipids ratio in IDL | 3 | Weighted median | 1.02628 | 1.00722-1.0457 | 0.00669 |
| prot-a-2129 | ADP-ribose pyrophosphatase, mitochondrial | Cholesterol to total lipids ratio in IDL | 3 | Weighted mode | 1.02615 | 1.00467-1.04808 | 0.13917 |
| prot-a-216 | Beta-1,4-galactosyltransferase 1 | Cholesterol to total lipids ratio in IDL | 5 | Inverse variance weighted | 1.04578 | 1.00103-1.09253 | 0.04482 |
| prot-a-216 | Beta-1,4-galactosyltransferase 1 | Cholesterol to total lipids ratio in IDL | 5 | MR Egger | 1.00845 | 0.86642-1.17378 | 0.9203 |
| prot-a-216 | Beta-1,4-galactosyltransferase 1 | Cholesterol to total lipids ratio in IDL | 5 | Weighted median | 1.0261 | 1.00355-1.04915 | 0.02305 |
| prot-a-216 | Beta-1,4-galactosyltransferase 1 | Cholesterol to total lipids ratio in IDL | 5 | Weighted mode | 1.02648 | 1.00518-1.04823 | 0.071 |
| prot-a-2398 | PH and SEC7 domain-containing protein 1 | Cholesterol to total lipids ratio in IDL | 4 | Inverse variance weighted | 1.11174 | 1.00025-1.23566 | 0.04947 |
| prot-a-2398 | PH and SEC7 domain-containing protein 1 | Cholesterol to total lipids ratio in IDL | 4 | MR Egger | 1.04532 | 0.8073-1.35352 | 0.76871 |
| prot-a-2398 | PH and SEC7 domain-containing protein 1 | Cholesterol to total lipids ratio in IDL | 4 | Weighted median | 1.06233 | 1.02827-1.09752 | 0.00028 |
| prot-a-2398 | PH and SEC7 domain-containing protein 1 | Cholesterol to total lipids ratio in IDL | 4 | Weighted mode | 1.11059 | 1.09001-1.13156 | 0.00161 |
| prot-a-2470 | Sulfhydryl oxidase 2 | Cholesterol to total lipids ratio in IDL | 3 | Inverse variance weighted | 1.03017 | 1.01285-1.04778 | 0.00059 |
| prot-a-2470 | Sulfhydryl oxidase 2 | Cholesterol to total lipids ratio in IDL | 3 | MR Egger | 1.04658 | 1.02496-1.06866 | 0.14627 |
| prot-a-2470 | Sulfhydryl oxidase 2 | Cholesterol to total lipids ratio in IDL | 3 | Weighted median | 1.02971 | 1.01997-1.03955 | 0 |
| prot-a-2470 | Sulfhydryl oxidase 2 | Cholesterol to total lipids ratio in IDL | 3 | Weighted mode | 1.03513 | 1.02449-1.04588 | 0.02253 |
| prot-a-2959 | Transferrin receptor protein 1 | Cholesterol to total lipids ratio in IDL | 4 | Inverse variance weighted | 1.0426 | 1.0054-1.08118 | 0.02443 |
| prot-a-2959 | Transferrin receptor protein 1 | Cholesterol to total lipids ratio in IDL | 4 | MR Egger | 1.1146 | 0.9268-1.34046 | 0.36826 |
| prot-a-2959 | Transferrin receptor protein 1 | Cholesterol to total lipids ratio in IDL | 4 | Weighted median | 1.03991 | 1.0057-1.07527 | 0.02184 |
| prot-a-2959 | Transferrin receptor protein 1 | Cholesterol to total lipids ratio in IDL | 4 | Weighted mode | 1.04174 | 0.99422-1.09153 | 0.18452 |
| prot-a-3028 | Tenascin | Cholesterol to total lipids ratio in IDL | 4 | Inverse variance weighted | 1.01394 | 1.00282-1.02517 | 0.01384 |
| prot-a-3028 | Tenascin | Cholesterol to total lipids ratio in IDL | 4 | MR Egger | 1.00963 | 0.98784-1.0319 | 0.48019 |
| prot-a-3028 | Tenascin | Cholesterol to total lipids ratio in IDL | 4 | Weighted median | 1.0151 | 1.00305-1.02729 | 0.01388 |
| prot-a-3028 | Tenascin | Cholesterol to total lipids ratio in IDL | 4 | Weighted mode | 1.01489 | 1.00218-1.02776 | 0.10509 |
| prot-a-3071 | Tenascin-X | Cholesterol to total lipids ratio in IDL | 3 | Inverse variance weighted | 1.01312 | 1.00271-1.02363 | 0.01337 |
| prot-a-3071 | Tenascin-X | Cholesterol to total lipids ratio in IDL | 3 | MR Egger | 1.00483 | 0.97934-1.03099 | 0.77562 |
| prot-a-3071 | Tenascin-X | Cholesterol to total lipids ratio in IDL | 3 | Weighted median | 1.01255 | 1.00143-1.02378 | 0.02678 |
| prot-a-3071 | Tenascin-X | Cholesterol to total lipids ratio in IDL | 3 | Weighted mode | 1.01209 | 1.00058-1.02373 | 0.17561 |
| prot-a-316 | Protein CEI | Cholesterol to total lipids ratio in IDL | 4 | Inverse variance weighted | 1.38883 | 1.1883-1.62319 | 4.00E-05 |
| prot-a-316 | Protein CEI | Cholesterol to total lipids ratio in IDL | 4 | MR Egger | 2.16508 | 1.44842-3.23632 | 0.06382 |
| prot-a-316 | Protein CEI | Cholesterol to total lipids ratio in IDL | 4 | Weighted median | 1.15258 | 1.11297-1.19359 | 0 |
| prot-a-316 | Protein CEI | Cholesterol to total lipids ratio in IDL | 4 | Weighted mode | 1.0811 | 1.05454-1.10833 | 0.00867 |
| prot-a-3200 | Selenoprotein S | Cholesterol to total lipids ratio in IDL | 3 | Inverse variance weighted | 1.58845 | 1.34407-1.87728 | 0 |
| prot-a-3200 | Selenoprotein S | Cholesterol to total lipids ratio in IDL | 3 | MR Egger | 2.08886 | 1.47901-2.95019 | 0.14943 |
| prot-a-3200 | Selenoprotein S | Cholesterol to total lipids ratio in IDL | 3 | Weighted median | 1.40741 | 1.29485-1.52975 | 0 |
| prot-a-3200 | Selenoprotein S | Cholesterol to total lipids ratio in IDL | 3 | Weighted mode | 1.62794 | 1.55443-1.70492 | 0.00233 |
| prot-a-426 | CD209 antigen | Cholesterol to total lipids ratio in IDL | 3 | Inverse variance weighted | 1.01735 | 1.00581-1.02903 | 0.00313 |
| prot-a-426 | CD209 antigen | Cholesterol to total lipids ratio in IDL | 3 | MR Egger | 1.02908 | 1.00497-1.05378 | 0.25423 |
| prot-a-426 | CD209 antigen | Cholesterol to total lipids ratio in IDL | 3 | Weighted median | 1.01815 | 1.00816-1.02823 | 0.00035 |
| prot-a-426 | CD209 antigen | Cholesterol to total lipids ratio in IDL | 3 | Weighted mode | 1.02111 | 1.01032-1.03201 | 0.06115 |
| prot-a-518 | Complement factor B | Cholesterol to total lipids ratio in IDL | 4 | Inverse variance weighted | 1.02081 | 1.00481-1.03705 | 0.01059 |
| prot-a-518 | Complement factor B | Cholesterol to total lipids ratio in IDL | 4 | MR Egger | 1.0135 | 0.98655-1.0412 | 0.43226 |
| prot-a-518 | Complement factor B | Cholesterol to total lipids ratio in IDL | 4 | Weighted median | 1.01619 | 0.99865-1.03404 | 0.07057 |
| prot-a-518 | Complement factor B | Cholesterol to total lipids ratio in IDL | 4 | Weighted mode | 1.01617 | 0.99618-1.03656 | 0.21165 |
| prot-a-1204 | GDNF family receptor alpha-like | Cholesterol to total lipids ratio in IDL | 3 | Inverse variance weighted | 0.95311 | 0.91031-0.99792 | 0.04049 |
| prot-a-1204 | GDNF family receptor alpha-like | Cholesterol to total lipids ratio in IDL | 3 | MR Egger | 0.85998 | 0.77042-0.95996 | 0.22672 |
| prot-a-1204 | GDNF family receptor alpha-like | Cholesterol to total lipids ratio in IDL | 3 | Weighted median | 0.9754 | 0.938-1.0143 | 0.21189 |
| prot-a-1204 | GDNF family receptor alpha-like | Cholesterol to total lipids ratio in IDL | 3 | Weighted mode | 0.97671 | 0.93466-1.02065 | 0.40406 |
| prot-a-1275 | Glutamate receptor 4 | Cholesterol to total lipids ratio in IDL | 3 | Inverse variance weighted | 0.9527 | 0.92945-0.97653 | 0.00012 |
| prot-a-1275 | Glutamate receptor 4 | Cholesterol to total lipids ratio in IDL | 3 | MR Egger | 0.98729 | 0.8489-1.14824 | 0.89527 |
| prot-a-1275 | Glutamate receptor 4 | Cholesterol to total lipids ratio in IDL | 3 | Weighted median | 0.96093 | 0.94158-0.98067 | 0.00012 |
| prot-a-1275 | Glutamate receptor 4 | Cholesterol to total lipids ratio in IDL | 3 | Weighted mode | 0.96541 | 0.94279-0.98857 | 0.10054 |
| prot-a-1347 | HLA class II histocompatibility antigen, DQ alpha 2 chain | Cholesterol to total lipids ratio in IDL | 4 | Inverse variance weighted | 0.94977 | 0.92649-0.97363 | 5.00E-05 |
| prot-a-1347 | HLA class II histocompatibility antigen, DQ alpha 2 chain | Cholesterol to total lipids ratio in IDL | 4 | MR Egger | 0.9396 | 0.88165-1.00136 | 0.19509 |
| prot-a-1347 | HLA class II histocompatibility antigen, DQ alpha 2 chain | Cholesterol to total lipids ratio in IDL | 4 | Weighted median | 0.94696 | 0.92992-0.96431 | 0 |
| prot-a-1347 | HLA class II histocompatibility antigen, DQ alpha 2 chain | Cholesterol to total lipids ratio in IDL | 4 | Weighted mode | 0.94356 | 0.92513-0.96236 | 0.01034 |
| prot-a-1506 | Interleukin-21 | Cholesterol to total lipids ratio in IDL | 4 | Inverse variance weighted | 0.96154 | 0.94179-0.98171 | 0.00021 |
| prot-a-1506 | Interleukin-21 | Cholesterol to total lipids ratio in IDL | 4 | MR Egger | 0.91674 | 0.86848-0.96769 | 0.08771 |
| prot-a-1506 | Interleukin-21 | Cholesterol to total lipids ratio in IDL | 4 | Weighted median | 0.96235 | 0.94058-0.98463 | 0.00101 |
| prot-a-1506 | Interleukin-21 | Cholesterol to total lipids ratio in IDL | 4 | Weighted mode | 0.96037 | 0.93754-0.98376 | 0.04595 |
| prot-a-1576 | Immunoglobulin superfamily containing leucine-rich repeat protein 2 | Cholesterol to total lipids ratio in IDL | 4 | Inverse variance weighted | 0.92649 | 0.91044-0.94283 | 0 |
| prot-a-1576 | Immunoglobulin superfamily containing leucine-rich repeat protein 2 | Cholesterol to total lipids ratio in IDL | 4 | MR Egger | 0.90117 | 0.85801-0.94649 | 0.05329 |
| prot-a-1576 | Immunoglobulin superfamily containing leucine-rich repeat protein 2 | Cholesterol to total lipids ratio in IDL | 4 | Weighted median | 0.92176 | 0.90217-0.94176 | 0 |
| prot-a-1576 | Immunoglobulin superfamily containing leucine-rich repeat protein 2 | Cholesterol to total lipids ratio in IDL | 4 | Weighted mode | 0.92096 | 0.8972-0.94535 | 0.00855 |
| prot-a-1744 | Leukocyte immunoglobulin-like receptor subfamily B member 2 | Cholesterol to total lipids ratio in IDL | 3 | Inverse variance weighted | 0.9817 | 0.96925-0.9943 | 0.00455 |
| prot-a-1744 | Leukocyte immunoglobulin-like receptor subfamily B member 2 | Cholesterol to total lipids ratio in IDL | 3 | MR Egger | 0.94379 | 0.85513-1.04164 | 0.45582 |
| prot-a-1744 | Leukocyte immunoglobulin-like receptor subfamily B member 2 | Cholesterol to total lipids ratio in IDL | 3 | Weighted median | 0.98105 | 0.96822-0.99406 | 0.00441 |
| prot-a-1744 | Leukocyte immunoglobulin-like receptor subfamily B member 2 | Cholesterol to total lipids ratio in IDL | 3 | Weighted mode | 0.98045 | 0.96747-0.9936 | 0.10092 |
| prot-a-1781 | alpha-2-macroglobulin receptor-associated protein | Cholesterol to total lipids ratio in IDL | 5 | Inverse variance weighted | 0.98493 | 0.97039-0.99969 | 0.04535 |
| prot-a-1781 | alpha-2-macroglobulin receptor-associated protein | Cholesterol to total lipids ratio in IDL | 5 | MR Egger | 0.97817 | 0.95793-0.99884 | 0.13039 |
| prot-a-1781 | alpha-2-macroglobulin receptor-associated protein | Cholesterol to total lipids ratio in IDL | 5 | Weighted median | 0.98351 | 0.97339-0.99373 | 0.00161 |
| prot-a-1781 | alpha-2-macroglobulin receptor-associated protein | Cholesterol to total lipids ratio in IDL | 5 | Weighted mode | 0.98367 | 0.97248-0.99498 | 0.04771 |
| prot-a-1898 | MHC class I polypeptide-related sequence B | Cholesterol to total lipids ratio in IDL | 4 | Inverse variance weighted | 0.96397 | 0.94131-0.98718 | 0.0025 |
| prot-a-1898 | MHC class I polypeptide-related sequence B | Cholesterol to total lipids ratio in IDL | 4 | MR Egger | 0.97949 | 0.9385-1.02228 | 0.44241 |
| prot-a-1898 | MHC class I polypeptide-related sequence B | Cholesterol to total lipids ratio in IDL | 4 | Weighted median | 0.96384 | 0.95037-0.9775 | 0 |
| prot-a-1898 | MHC class I polypeptide-related sequence B | Cholesterol to total lipids ratio in IDL | 4 | Weighted mode | 0.96246 | 0.94825-0.97689 | 0.01506 |
| prot-a-2071 | Epididymal secretory protein E1 | Cholesterol to total lipids ratio in IDL | 3 | Inverse variance weighted | 0.98754 | 0.97826-0.99691 | 0.00925 |
| prot-a-2071 | Epididymal secretory protein E1 | Cholesterol to total lipids ratio in IDL | 3 | MR Egger | 0.98332 | 0.97041-0.99641 | 0.24277 |
| prot-a-2071 | Epididymal secretory protein E1 | Cholesterol to total lipids ratio in IDL | 3 | Weighted median | 0.98697 | 0.97765-0.99637 | 0.00668 |
| prot-a-2071 | Epididymal secretory protein E1 | Cholesterol to total lipids ratio in IDL | 3 | Weighted mode | 0.98659 | 0.97698-0.9963 | 0.11395 |
| prot-a-2243 | Platelet endothelial aggregation receptor 1 | Cholesterol to total lipids ratio in IDL | 3 | Inverse variance weighted | 0.95125 | 0.9193-0.98432 | 0.00415 |
| prot-a-2243 | Platelet endothelial aggregation receptor 1 | Cholesterol to total lipids ratio in IDL | 3 | MR Egger | 0.90224 | 0.7596-1.07166 | 0.44975 |
| prot-a-2243 | Platelet endothelial aggregation receptor 1 | Cholesterol to total lipids ratio in IDL | 3 | Weighted median | 0.95799 | 0.92753-0.98946 | 0.00925 |
| prot-a-2243 | Platelet endothelial aggregation receptor 1 | Cholesterol to total lipids ratio in IDL | 3 | Weighted mode | 0.96207 | 0.92437-1.00131 | 0.19848 |
| prot-a-2392 | Trypsin-2 | Cholesterol to total lipids ratio in IDL | 3 | Inverse variance weighted | 0.96459 | 0.94048-0.98932 | 0.00524 |
| prot-a-2392 | Trypsin-2 | Cholesterol to total lipids ratio in IDL | 3 | MR Egger | 0.9349 | 0.82079-1.06488 | 0.49574 |
| prot-a-2392 | Trypsin-2 | Cholesterol to total lipids ratio in IDL | 3 | Weighted median | 0.9709 | 0.94218-1.0005 | 0.05394 |
| prot-a-2392 | Trypsin-2 | Cholesterol to total lipids ratio in IDL | 3 | Weighted mode | 0.97452 | 0.94202-1.00813 | 0.27425 |
| prot-a-2667 | P-selectin | Cholesterol to total lipids ratio in IDL | 6 | Inverse variance weighted | 0.96479 | 0.93934-0.99094 | 0.00861 |
| prot-a-2667 | P-selectin | Cholesterol to total lipids ratio in IDL | 6 | MR Egger | 0.96482 | 0.91098-1.02185 | 0.28871 |
| prot-a-2667 | P-selectin | Cholesterol to total lipids ratio in IDL | 6 | Weighted median | 0.97491 | 0.96019-0.98986 | 0.00107 |
| prot-a-2667 | P-selectin | Cholesterol to total lipids ratio in IDL | 6 | Weighted mode | 0.97619 | 0.95922-0.99345 | 0.04307 |
| prot-a-3203 | Vacuolar protein sorting-associated protein 29 | Cholesterol to total lipids ratio in IDL | 3 | Inverse variance weighted | 0.74357 | 0.70445-0.78485 | 0 |
| prot-a-3203 | Vacuolar protein sorting-associated protein 29 | Cholesterol to total lipids ratio in IDL | 3 | MR Egger | 0.72094 | 0.70925-0.73282 | 0.01622 |
| prot-a-3203 | Vacuolar protein sorting-associated protein 29 | Cholesterol to total lipids ratio in IDL | 3 | Weighted median | 0.74036 | 0.72783-0.7531 | 0 |
| prot-a-3203 | Vacuolar protein sorting-associated protein 29 | Cholesterol to total lipids ratio in IDL | 3 | Weighted mode | 0.73929 | 0.72741-0.75137 | 0.00075 |
| prot-a-373 | Carbonyl reductase [NADPH] 1 | Cholesterol to total lipids ratio in IDL | 3 | Inverse variance weighted | 0.98127 | 0.96311-0.99978 | 0.04734 |
| prot-a-373 | Carbonyl reductase [NADPH] 1 | Cholesterol to total lipids ratio in IDL | 3 | MR Egger | 0.9866 | 0.95706-1.01705 | 0.54419 |
| prot-a-373 | Carbonyl reductase [NADPH] 1 | Cholesterol to total lipids ratio in IDL | 3 | Weighted median | 0.98073 | 0.96136-1.00049 | 0.05594 |
| prot-a-373 | Carbonyl reductase [NADPH] 1 | Cholesterol to total lipids ratio in IDL | 3 | Weighted mode | 0.98457 | 0.96149-1.00822 | 0.32777 |
| prot-a-524 | Cofilin-1 | Cholesterol to total lipids ratio in IDL | 3 | Inverse variance weighted | 0.98232 | 0.96618-0.99873 | 0.03485 |
| prot-a-524 | Cofilin-1 | Cholesterol to total lipids ratio in IDL | 3 | MR Egger | 0.99759 | 0.93668-1.06247 | 0.95239 |
| prot-a-524 | Cofilin-1 | Cholesterol to total lipids ratio in IDL | 3 | Weighted median | 0.98274 | 0.96573-1.00006 | 0.05079 |
| prot-a-524 | Cofilin-1 | Cholesterol to total lipids ratio in IDL | 3 | Weighted mode | 0.98296 | 0.96345-1.00285 | 0.23474 |
| prot-a-543 | Chitotriosidase-1 | Cholesterol to total lipids ratio in IDL | 3 | Inverse variance weighted | 0.98999 | 0.98141-0.99864 | 0.02344 |
| prot-a-543 | Chitotriosidase-1 | Cholesterol to total lipids ratio in IDL | 3 | MR Egger | 0.99517 | 0.98276-1.00775 | 0.58817 |
| prot-a-543 | Chitotriosidase-1 | Cholesterol to total lipids ratio in IDL | 3 | Weighted median | 0.99023 | 0.98158-0.99896 | 0.02839 |
| prot-a-543 | Chitotriosidase-1 | Cholesterol to total lipids ratio in IDL | 3 | Weighted mode | 0.99107 | 0.98299-0.99921 | 0.16463 |
| prot-a-627 | Collectin-10 | Cholesterol to total lipids ratio in IDL | 3 | Inverse variance weighted | 0.66871 | 0.44726-0.99981 | 0.04989 |
| prot-a-627 | Collectin-10 | Cholesterol to total lipids ratio in IDL | 3 | MR Egger | 0.48948 | 0.24646-0.97212 | 0.29006 |
| prot-a-627 | Collectin-10 | Cholesterol to total lipids ratio in IDL | 3 | Weighted median | 0.9477 | 0.92437-0.97163 | 2.00E-05 |
| prot-a-627 | Collectin-10 | Cholesterol to total lipids ratio in IDL | 3 | Weighted mode | 0.95335 | 0.93064-0.97661 | 0.06035 |
| prot-a-836 | DnaJ homolog subfamily B member 11 | Cholesterol to total lipids ratio in IDL | 3 | Inverse variance weighted | 0.96103 | 0.9275-0.99577 | 0.02823 |
| prot-a-836 | DnaJ homolog subfamily B member 11 | Cholesterol to total lipids ratio in IDL | 3 | MR Egger | 0.94651 | 0.85513-1.04765 | 0.48106 |
| prot-a-836 | DnaJ homolog subfamily B member 11 | Cholesterol to total lipids ratio in IDL | 3 | Weighted median | 0.95067 | 0.92034-0.982 | 0.00223 |
| prot-a-836 | DnaJ homolog subfamily B member 11 | Cholesterol to total lipids ratio in IDL | 3 | Weighted mode | 0.94705 | 0.91086-0.98467 | 0.11157 |
| prot-b-50 | C-C motif chemokine ligand 4 | Cholesterol to total lipids ratio in IDL | 3 | Inverse variance weighted | 0.97363 | 0.95433-0.99331 | 0.00887 |
| prot-b-50 | C-C motif chemokine ligand 4 | Cholesterol to total lipids ratio in IDL | 3 | MR Egger | 0.86709 | 0.75503-0.9958 | 0.29269 |
| prot-b-50 | C-C motif chemokine ligand 4 | Cholesterol to total lipids ratio in IDL | 3 | Weighted median | 0.97862 | 0.96218-0.99533 | 0.01238 |
| prot-b-50 | C-C motif chemokine ligand 4 | Cholesterol to total lipids ratio in IDL | 3 | Weighted mode | 0.97933 | 0.95986-0.99919 | 0.17829 |
| prot-a-10 | Histo-blood group ABO system transferase | Polyunsaturated fatty acids | 3 | Inverse variance weighted | 1.01952 | 1.01245-1.02663 | 0 |
| prot-a-10 | Histo-blood group ABO system transferase | Polyunsaturated fatty acids | 3 | MR Egger | 1.01297 | 1.00091-1.02518 | 0.28184 |
| prot-a-10 | Histo-blood group ABO system transferase | Polyunsaturated fatty acids | 3 | Weighted median | 1.01929 | 1.01277-1.02585 | 0 |
| prot-a-10 | Histo-blood group ABO system transferase | Polyunsaturated fatty acids | 3 | Weighted mode | 1.01842 | 1.01221-1.02466 | 0.02798 |
| prot-a-1275 | Glutamate receptor 4 | Polyunsaturated fatty acids | 3 | Inverse variance weighted | 1.06343 | 1.04101-1.08634 | 0 |
| prot-a-1275 | Glutamate receptor 4 | Polyunsaturated fatty acids | 3 | MR Egger | 1.11663 | 1.00451-1.24127 | 0.28975 |
| prot-a-1275 | Glutamate receptor 4 | Polyunsaturated fatty acids | 3 | Weighted median | 1.06655 | 1.04684-1.08662 | 0 |
| prot-a-1275 | Glutamate receptor 4 | Polyunsaturated fatty acids | 3 | Weighted mode | 1.06697 | 1.04683-1.0875 | 0.02176 |
| prot-a-1277 | Granulins | Polyunsaturated fatty acids | 4 | Inverse variance weighted | 1.05032 | 1.00092-1.10216 | 0.04576 |
| prot-a-1277 | Granulins | Polyunsaturated fatty acids | 4 | MR Egger | 1.12145 | 1.07941-1.16513 | 0.02773 |
| prot-a-1277 | Granulins | Polyunsaturated fatty acids | 4 | Weighted median | 1.05096 | 1.03816-1.06391 | 0 |
| prot-a-1277 | Granulins | Polyunsaturated fatty acids | 4 | Weighted mode | 1.0732 | 1.05932-1.08725 | 0.00177 |
| prot-a-1347 | HLA class II histocompatibility antigen, DQ alpha 2 chain | Polyunsaturated fatty acids | 4 | Inverse variance weighted | 1.05979 | 1.01671-1.10471 | 0.0061 |
| prot-a-1347 | HLA class II histocompatibility antigen, DQ alpha 2 chain | Polyunsaturated fatty acids | 4 | MR Egger | 1.12454 | 1.05951-1.19355 | 0.06096 |
| prot-a-1347 | HLA class II histocompatibility antigen, DQ alpha 2 chain | Polyunsaturated fatty acids | 4 | Weighted median | 1.06966 | 1.04715-1.09265 | 0 |
| prot-a-1347 | HLA class II histocompatibility antigen, DQ alpha 2 chain | Polyunsaturated fatty acids | 4 | Weighted mode | 1.07676 | 1.05649-1.09742 | 0.00468 |
| prot-a-1506 | Interleukin-21 | Polyunsaturated fatty acids | 4 | Inverse variance weighted | 1.05436 | 1.00191-1.10955 | 0.04203 |
| prot-a-1506 | Interleukin-21 | Polyunsaturated fatty acids | 4 | MR Egger | 1.10971 | 0.92959-1.32473 | 0.36844 |
| prot-a-1506 | Interleukin-21 | Polyunsaturated fatty acids | 4 | Weighted median | 1.05892 | 1.03009-1.08855 | 5.00E-05 |
| prot-a-1506 | Interleukin-21 | Polyunsaturated fatty acids | 4 | Weighted mode | 1.08522 | 1.03005-1.14335 | 0.05448 |
| prot-a-1898 | MHC class I polypeptide-related sequence B | Polyunsaturated fatty acids | 4 | Inverse variance weighted | 1.05707 | 1.02979-1.08507 | 3.00E-05 |
| prot-a-1898 | MHC class I polypeptide-related sequence B | Polyunsaturated fatty acids | 4 | MR Egger | 1.0606 | 1.00352-1.12093 | 0.17249 |
| prot-a-1898 | MHC class I polypeptide-related sequence B | Polyunsaturated fatty acids | 4 | Weighted median | 1.06029 | 1.04547-1.07532 | 0 |
| prot-a-1898 | MHC class I polypeptide-related sequence B | Polyunsaturated fatty acids | 4 | Weighted mode | 1.06611 | 1.04963-1.08285 | 0.004 |
| prot-a-1920 | Neutrophil collagenase | Polyunsaturated fatty acids | 5 | Inverse variance weighted | 1.02476 | 1.009-1.04076 | 0.00198 |
| prot-a-1920 | Neutrophil collagenase | Polyunsaturated fatty acids | 5 | MR Egger | 1.01121 | 0.99085-1.03199 | 0.36156 |
| prot-a-1920 | Neutrophil collagenase | Polyunsaturated fatty acids | 5 | Weighted median | 1.02483 | 1.01444-1.03533 | 0 |
| prot-a-1920 | Neutrophil collagenase | Polyunsaturated fatty acids | 5 | Weighted mode | 1.02368 | 1.01291-1.03456 | 0.01227 |
| prot-a-1991 | N-acetylated-alpha-linked acidic dipeptidase 2 | Polyunsaturated fatty acids | 3 | Inverse variance weighted | 1.02841 | 1.00767-1.04958 | 0.00705 |
| prot-a-1991 | N-acetylated-alpha-linked acidic dipeptidase 2 | Polyunsaturated fatty acids | 3 | MR Egger | 1.05966 | 0.97912-1.14681 | 0.38708 |
| prot-a-1991 | N-acetylated-alpha-linked acidic dipeptidase 2 | Polyunsaturated fatty acids | 3 | Weighted median | 1.02878 | 1.00445-1.05371 | 0.02016 |
| prot-a-1991 | N-acetylated-alpha-linked acidic dipeptidase 2 | Polyunsaturated fatty acids | 3 | Weighted mode | 1.03436 | 1.00657-1.06292 | 0.13563 |
| prot-a-216 | Beta-1,4-galactosyltransferase 1 | Polyunsaturated fatty acids | 5 | Inverse variance weighted | 1.04734 | 1.00213-1.09459 | 0.03992 |
| prot-a-216 | Beta-1,4-galactosyltransferase 1 | Polyunsaturated fatty acids | 5 | MR Egger | 1.00296 | 0.86273-1.16598 | 0.97177 |
| prot-a-216 | Beta-1,4-galactosyltransferase 1 | Polyunsaturated fatty acids | 5 | Weighted median | 1.02333 | 1.00113-1.04603 | 0.03931 |
| prot-a-216 | Beta-1,4-galactosyltransferase 1 | Polyunsaturated fatty acids | 5 | Weighted mode | 1.02224 | 0.99824-1.04682 | 0.14379 |
| prot-a-2172 | Peptidyl-glycine alpha-amidating monooxygenase | Polyunsaturated fatty acids | 6 | Inverse variance weighted | 1.01337 | 1.00024-1.02667 | 0.04595 |
| prot-a-2172 | Peptidyl-glycine alpha-amidating monooxygenase | Polyunsaturated fatty acids | 6 | MR Egger | 1.02107 | 0.99057-1.05252 | 0.24902 |
| prot-a-2172 | Peptidyl-glycine alpha-amidating monooxygenase | Polyunsaturated fatty acids | 6 | Weighted median | 1.0113 | 0.99563-1.02721 | 0.15858 |
| prot-a-2172 | Peptidyl-glycine alpha-amidating monooxygenase | Polyunsaturated fatty acids | 6 | Weighted mode | 1.01134 | 0.99465-1.0283 | 0.24142 |
| prot-a-2221 | cAMP-specific 3',5'-cyclic phosphodiesterase 4D | Polyunsaturated fatty acids | 4 | Inverse variance weighted | 1.05718 | 1.02646-1.08883 | 0.00022 |
| prot-a-2221 | cAMP-specific 3',5'-cyclic phosphodiesterase 4D | Polyunsaturated fatty acids | 4 | MR Egger | 1.05456 | 0.97471-1.14095 | 0.31701 |
| prot-a-2221 | cAMP-specific 3',5'-cyclic phosphodiesterase 4D | Polyunsaturated fatty acids | 4 | Weighted median | 1.06317 | 1.04229-1.08447 | 0 |
| prot-a-2221 | cAMP-specific 3',5'-cyclic phosphodiesterase 4D | Polyunsaturated fatty acids | 4 | Weighted mode | 1.06355 | 1.04248-1.08505 | 0.00912 |
| prot-a-2398 | PH and SEC7 domain-containing protein 1 | Polyunsaturated fatty acids | 4 | Inverse variance weighted | 1.10727 | 1.03267-1.18726 | 0.00419 |
| prot-a-2398 | PH and SEC7 domain-containing protein 1 | Polyunsaturated fatty acids | 4 | MR Egger | 1.11241 | 0.92719-1.33464 | 0.37026 |
| prot-a-2398 | PH and SEC7 domain-containing protein 1 | Polyunsaturated fatty acids | 4 | Weighted median | 1.08635 | 1.05728-1.11622 | 0 |
| prot-a-2398 | PH and SEC7 domain-containing protein 1 | Polyunsaturated fatty acids | 4 | Weighted mode | 1.10819 | 1.07779-1.13944 | 0.00544 |
| prot-a-2470 | Sulfhydryl oxidase 2 | Polyunsaturated fatty acids | 3 | Inverse variance weighted | 1.03151 | 1.02191-1.0412 | 0 |
| prot-a-2470 | Sulfhydryl oxidase 2 | Polyunsaturated fatty acids | 3 | MR Egger | 1.03641 | 1.01925-1.05387 | 0.14891 |
| prot-a-2470 | Sulfhydryl oxidase 2 | Polyunsaturated fatty acids | 3 | Weighted median | 1.03178 | 1.02177-1.04188 | 0 |
| prot-a-2470 | Sulfhydryl oxidase 2 | Polyunsaturated fatty acids | 3 | Weighted mode | 1.03242 | 1.02206-1.0429 | 0.02506 |
| prot-a-2948 | Testis-expressed sequence 29 protein | Polyunsaturated fatty acids | 5 | Inverse variance weighted | 1.01566 | 1.00052-1.03103 | 0.04259 |
| prot-a-2948 | Testis-expressed sequence 29 protein | Polyunsaturated fatty acids | 5 | MR Egger | 1.04224 | 1.01334-1.07196 | 0.06332 |
| prot-a-2948 | Testis-expressed sequence 29 protein | Polyunsaturated fatty acids | 5 | Weighted median | 1.02266 | 1.00742-1.03813 | 0.00344 |
| prot-a-2948 | Testis-expressed sequence 29 protein | Polyunsaturated fatty acids | 5 | Weighted mode | 1.02439 | 1.00864-1.04038 | 0.03808 |
| prot-a-303 | Complement C1q tumor necrosis factor-related protein 1 | Polyunsaturated fatty acids | 6 | Inverse variance weighted | 1.05983 | 1.01313-1.10869 | 0.0115 |
| prot-a-303 | Complement C1q tumor necrosis factor-related protein 1 | Polyunsaturated fatty acids | 6 | MR Egger | 1.13387 | 1.06562-1.20649 | 0.01658 |
| prot-a-303 | Complement C1q tumor necrosis factor-related protein 1 | Polyunsaturated fatty acids | 6 | Weighted median | 1.06492 | 1.04086-1.08953 | 0 |
| prot-a-303 | Complement C1q tumor necrosis factor-related protein 1 | Polyunsaturated fatty acids | 6 | Weighted mode | 1.08961 | 1.0714-1.10812 | 0.00017 |
| prot-a-3048 | Tumor necrosis factor receptor superfamily member 1B | Polyunsaturated fatty acids | 3 | Inverse variance weighted | 1.03564 | 1.00472-1.0675 | 0.02353 |
| prot-a-3048 | Tumor necrosis factor receptor superfamily member 1B | Polyunsaturated fatty acids | 3 | MR Egger | 1.02495 | 0.93828-1.11963 | 0.68145 |
| prot-a-3048 | Tumor necrosis factor receptor superfamily member 1B | Polyunsaturated fatty acids | 3 | Weighted median | 1.02883 | 0.99233-1.06668 | 0.12304 |
| prot-a-3048 | Tumor necrosis factor receptor superfamily member 1B | Polyunsaturated fatty acids | 3 | Weighted mode | 1.02556 | 0.98279-1.0702 | 0.36534 |
| prot-a-3123 | Thioredoxin domain-containing protein 12 | Polyunsaturated fatty acids | 7 | Inverse variance weighted | 1.01723 | 1.00814-1.0264 | 0.00019 |
| prot-a-3123 | Thioredoxin domain-containing protein 12 | Polyunsaturated fatty acids | 7 | MR Egger | 1.02352 | 1.00827-1.03899 | 0.0289 |
| prot-a-3123 | Thioredoxin domain-containing protein 12 | Polyunsaturated fatty acids | 7 | Weighted median | 1.01613 | 1.00512-1.02727 | 0.00401 |
| prot-a-3123 | Thioredoxin domain-containing protein 12 | Polyunsaturated fatty acids | 7 | Weighted mode | 1.0172 | 1.00598-1.02855 | 0.02361 |
| prot-a-316 | Protein CEI | Polyunsaturated fatty acids | 4 | Inverse variance weighted | 1.13035 | 1.09033-1.17184 | 0 |
| prot-a-316 | Protein CEI | Polyunsaturated fatty acids | 4 | MR Egger | 1.25986 | 1.16514-1.36228 | 0.02853 |
| prot-a-316 | Protein CEI | Polyunsaturated fatty acids | 4 | Weighted median | 1.14326 | 1.12724-1.15951 | 0 |
| prot-a-316 | Protein CEI | Polyunsaturated fatty acids | 4 | Weighted mode | 1.14359 | 1.12827-1.15912 | 0.00029 |
| prot-a-3180 | Ubiquitin carboxyl-terminal hydrolase 25 | Polyunsaturated fatty acids | 3 | Inverse variance weighted | 1.02177 | 1.00772-1.03602 | 0.0023 |
| prot-a-3180 | Ubiquitin carboxyl-terminal hydrolase 25 | Polyunsaturated fatty acids | 3 | MR Egger | 1.01971 | 0.99428-1.04578 | 0.37147 |
| prot-a-3180 | Ubiquitin carboxyl-terminal hydrolase 25 | Polyunsaturated fatty acids | 3 | Weighted median | 1.02143 | 1.00744-1.03562 | 0.00258 |
| prot-a-3180 | Ubiquitin carboxyl-terminal hydrolase 25 | Polyunsaturated fatty acids | 3 | Weighted mode | 1.02131 | 1.00603-1.03683 | 0.11128 |
| prot-a-32 | A disintegrin and metalloproteinase with thrombospondin motifs 13 | Polyunsaturated fatty acids | 3 | Inverse variance weighted | 1.0155 | 1.0015-1.02969 | 0.02991 |
| prot-a-32 | A disintegrin and metalloproteinase with thrombospondin motifs 13 | Polyunsaturated fatty acids | 3 | MR Egger | 1.00042 | 0.97499-1.0265 | 0.97988 |
| prot-a-32 | A disintegrin and metalloproteinase with thrombospondin motifs 13 | Polyunsaturated fatty acids | 3 | Weighted median | 1.01222 | 0.99848-1.02615 | 0.08151 |
| prot-a-32 | A disintegrin and metalloproteinase with thrombospondin motifs 13 | Polyunsaturated fatty acids | 3 | Weighted mode | 1.01065 | 0.99481-1.02675 | 0.31926 |
| prot-a-3200 | Selenoprotein S | Polyunsaturated fatty acids | 3 | Inverse variance weighted | 1.18137 | 1.13235-1.2325 | 0 |
| prot-a-3200 | Selenoprotein S | Polyunsaturated fatty acids | 3 | MR Egger | 1.21968 | 1.04338-1.42577 | 0.24284 |
| prot-a-3200 | Selenoprotein S | Polyunsaturated fatty acids | 3 | Weighted median | 1.17702 | 1.15382-1.20069 | 0 |
| prot-a-3200 | Selenoprotein S | Polyunsaturated fatty acids | 3 | Weighted mode | 1.18319 | 1.1583-1.20861 | 0.00413 |
| prot-a-426 | CD209 antigen | Polyunsaturated fatty acids | 3 | Inverse variance weighted | 1.02517 | 1.01532-1.03511 | 0 |
| prot-a-426 | CD209 antigen | Polyunsaturated fatty acids | 3 | MR Egger | 1.03495 | 1.01394-1.05638 | 0.18818 |
| prot-a-426 | CD209 antigen | Polyunsaturated fatty acids | 3 | Weighted median | 1.02601 | 1.01634-1.03576 | 0 |
| prot-a-426 | CD209 antigen | Polyunsaturated fatty acids | 3 | Weighted mode | 1.0283 | 1.01748-1.03923 | 0.0354 |
| prot-a-592 | Colipase | Polyunsaturated fatty acids | 3 | Inverse variance weighted | 1.02595 | 1.00627-1.04601 | 0.00951 |
| prot-a-592 | Colipase | Polyunsaturated fatty acids | 3 | MR Egger | 1.06469 | 1.00833-1.12421 | 0.2653 |
| prot-a-592 | Colipase | Polyunsaturated fatty acids | 3 | Weighted median | 1.0291 | 1.0109-1.04762 | 0.00162 |
| prot-a-592 | Colipase | Polyunsaturated fatty acids | 3 | Weighted mode | 1.03101 | 1.01191-1.05047 | 0.08531 |
| prot-a-794 | Beta-defensin 119 | Polyunsaturated fatty acids | 7 | Inverse variance weighted | 1.06356 | 1.01471-1.11476 | 0.01021 |
| prot-a-794 | Beta-defensin 119 | Polyunsaturated fatty acids | 7 | MR Egger | 1.01034 | 0.90585-1.12689 | 0.8607 |
| prot-a-794 | Beta-defensin 119 | Polyunsaturated fatty acids | 7 | Weighted median | 1.03749 | 1.01708-1.0583 | 0.00028 |
| prot-a-794 | Beta-defensin 119 | Polyunsaturated fatty acids | 7 | Weighted mode | 1.03334 | 1.01156-1.0556 | 0.02349 |
| prot-a-892 | Endothelin-2 | Polyunsaturated fatty acids | 3 | Inverse variance weighted | 1.03679 | 1.0069-1.06756 | 0.01549 |
| prot-a-892 | Endothelin-2 | Polyunsaturated fatty acids | 3 | MR Egger | 1.10654 | 1.03882-1.17867 | 0.19616 |
| prot-a-892 | Endothelin-2 | Polyunsaturated fatty acids | 3 | Weighted median | 1.03511 | 1.0124-1.05832 | 0.0023 |
| prot-a-892 | Endothelin-2 | Polyunsaturated fatty acids | 3 | Weighted mode | 1.0519 | 1.02706-1.07734 | 0.05346 |
| prot-a-1148 | Ferritin | Polyunsaturated fatty acids | 3 | Inverse variance weighted | 0.9502 | 0.92382-0.97733 | 0.00038 |
| prot-a-1148 | Ferritin | Polyunsaturated fatty acids | 3 | MR Egger | 0.98411 | 0.95205-1.01725 | 0.51706 |
| prot-a-1148 | Ferritin | Polyunsaturated fatty acids | 3 | Weighted median | 0.95108 | 0.93388-0.9686 | 0 |
| prot-a-1148 | Ferritin | Polyunsaturated fatty acids | 3 | Weighted mode | 0.96125 | 0.94269-0.98017 | 0.05787 |
| prot-a-1369 | Haptoglobin | Polyunsaturated fatty acids | 5 | Inverse variance weighted | 0.97195 | 0.96248-0.98151 | 0 |
| prot-a-1369 | Haptoglobin | Polyunsaturated fatty acids | 5 | MR Egger | 0.99242 | 0.96898-1.01642 | 0.57674 |
| prot-a-1369 | Haptoglobin | Polyunsaturated fatty acids | 5 | Weighted median | 0.97516 | 0.96452-0.98591 | 1.00E-05 |
| prot-a-1369 | Haptoglobin | Polyunsaturated fatty acids | 5 | Weighted mode | 0.97444 | 0.96398-0.98502 | 0.0093 |
| prot-a-1530 | Interleukin-3 receptor subunit alpha | Polyunsaturated fatty acids | 3 | Inverse variance weighted | 0.95436 | 0.9141-0.9964 | 0.03366 |
| prot-a-1530 | Interleukin-3 receptor subunit alpha | Polyunsaturated fatty acids | 3 | MR Egger | 0.98939 | 0.95076-1.0296 | 0.69234 |
| prot-a-1530 | Interleukin-3 receptor subunit alpha | Polyunsaturated fatty acids | 3 | Weighted median | 0.95961 | 0.94827-0.97108 | 0 |
| prot-a-1530 | Interleukin-3 receptor subunit alpha | Polyunsaturated fatty acids | 3 | Weighted mode | 0.95971 | 0.94755-0.97204 | 0.02415 |
| prot-a-1542 | Interleukin-6 receptor subunit beta | Polyunsaturated fatty acids | 4 | Inverse variance weighted | 0.93002 | 0.87577-0.98763 | 0.01799 |
| prot-a-1542 | Interleukin-6 receptor subunit beta | Polyunsaturated fatty acids | 4 | MR Egger | 0.98306 | 0.79898-1.20954 | 0.8865 |
| prot-a-1542 | Interleukin-6 receptor subunit beta | Polyunsaturated fatty acids | 4 | Weighted median | 0.95527 | 0.92551-0.98597 | 0.00458 |
| prot-a-1542 | Interleukin-6 receptor subunit beta | Polyunsaturated fatty acids | 4 | Weighted mode | 0.96472 | 0.93566-0.99468 | 0.10481 |
| prot-a-1576 | Immunoglobulin superfamily containing leucine-rich repeat protein 2 | Polyunsaturated fatty acids | 4 | Inverse variance weighted | 0.93681 | 0.90497-0.96978 | 0.00022 |
| prot-a-1576 | Immunoglobulin superfamily containing leucine-rich repeat protein 2 | Polyunsaturated fatty acids | 4 | MR Egger | 0.90339 | 0.81252-1.00442 | 0.2011 |
| prot-a-1576 | Immunoglobulin superfamily containing leucine-rich repeat protein 2 | Polyunsaturated fatty acids | 4 | Weighted median | 0.9223 | 0.9022-0.94284 | 0 |
| prot-a-1576 | Immunoglobulin superfamily containing leucine-rich repeat protein 2 | Polyunsaturated fatty acids | 4 | Weighted mode | 0.92353 | 0.9033-0.94422 | 0.00589 |
| prot-a-1622 | Vascular endothelial growth factor receptor 2 | Polyunsaturated fatty acids | 4 | Inverse variance weighted | 0.96529 | 0.93718-0.99425 | 0.01914 |
| prot-a-1622 | Vascular endothelial growth factor receptor 2 | Polyunsaturated fatty acids | 4 | MR Egger | 0.9755 | 0.91401-1.04113 | 0.53305 |
| prot-a-1622 | Vascular endothelial growth factor receptor 2 | Polyunsaturated fatty acids | 4 | Weighted median | 0.97299 | 0.95296-0.99343 | 0.00984 |
| prot-a-1622 | Vascular endothelial growth factor receptor 2 | Polyunsaturated fatty acids | 4 | Weighted mode | 0.98963 | 0.96013-1.02003 | 0.54793 |
| prot-a-1645 | Killer cell immunoglobulin-like receptor 2DS2 | Polyunsaturated fatty acids | 8 | Inverse variance weighted | 0.94522 | 0.91816-0.97307 | 0.00014 |
| prot-a-1645 | Killer cell immunoglobulin-like receptor 2DS2 | Polyunsaturated fatty acids | 8 | MR Egger | 0.9164 | 0.8599-0.97662 | 0.03612 |
| prot-a-1645 | Killer cell immunoglobulin-like receptor 2DS2 | Polyunsaturated fatty acids | 8 | Weighted median | 0.95597 | 0.92961-0.98309 | 0.0016 |
| prot-a-1645 | Killer cell immunoglobulin-like receptor 2DS2 | Polyunsaturated fatty acids | 8 | Weighted mode | 0.98564 | 0.93732-1.03645 | 0.59031 |
| prot-a-1739 | Leukocyte immunoglobulin-like receptor subfamily A member 5 | Polyunsaturated fatty acids | 3 | Inverse variance weighted | 0.98076 | 0.96741-0.99429 | 0.00545 |
| prot-a-1739 | Leukocyte immunoglobulin-like receptor subfamily A member 5 | Polyunsaturated fatty acids | 3 | MR Egger | 0.98119 | 0.95082-1.01254 | 0.44665 |
| prot-a-1739 | Leukocyte immunoglobulin-like receptor subfamily A member 5 | Polyunsaturated fatty acids | 3 | Weighted median | 0.98115 | 0.96731-0.99518 | 0.00862 |
| prot-a-1739 | Leukocyte immunoglobulin-like receptor subfamily A member 5 | Polyunsaturated fatty acids | 3 | Weighted mode | 0.98168 | 0.96607-0.99754 | 0.15218 |
| prot-a-1791 | Leucine-rich repeat serine/threonine-protein kinase 2 | Polyunsaturated fatty acids | 4 | Inverse variance weighted | 0.96823 | 0.93803-0.99939 | 0.04576 |
| prot-a-1791 | Leucine-rich repeat serine/threonine-protein kinase 2 | Polyunsaturated fatty acids | 4 | MR Egger | 0.94326 | 0.79876-1.11391 | 0.56229 |
| prot-a-1791 | Leucine-rich repeat serine/threonine-protein kinase 2 | Polyunsaturated fatty acids | 4 | Weighted median | 0.97115 | 0.94676-0.99616 | 0.02404 |
| prot-a-1791 | Leucine-rich repeat serine/threonine-protein kinase 2 | Polyunsaturated fatty acids | 4 | Weighted mode | 0.9459 | 0.92113-0.97135 | 0.02614 |
| prot-a-1902 | MICOS complex subunit MIC10 | Polyunsaturated fatty acids | 3 | Inverse variance weighted | 0.98663 | 0.97743-0.99592 | 0.00488 |
| prot-a-1902 | MICOS complex subunit MIC10 | Polyunsaturated fatty acids | 3 | MR Egger | 0.97962 | 0.96783-0.99155 | 0.18554 |
| prot-a-1902 | MICOS complex subunit MIC10 | Polyunsaturated fatty acids | 3 | Weighted median | 0.98662 | 0.97992-0.99337 | 0.00011 |
| prot-a-1902 | MICOS complex subunit MIC10 | Polyunsaturated fatty acids | 3 | Weighted mode | 0.98546 | 0.97851-0.99247 | 0.05584 |
| prot-a-220 | Beta-1,4-galactosyltransferase 6 | Polyunsaturated fatty acids | 3 | Inverse variance weighted | 0.98034 | 0.96449-0.99645 | 0.01698 |
| prot-a-220 | Beta-1,4-galactosyltransferase 6 | Polyunsaturated fatty acids | 3 | MR Egger | 0.97063 | 0.94944-0.99228 | 0.22986 |
| prot-a-220 | Beta-1,4-galactosyltransferase 6 | Polyunsaturated fatty acids | 3 | Weighted median | 0.97737 | 0.96112-0.99389 | 0.00743 |
| prot-a-220 | Beta-1,4-galactosyltransferase 6 | Polyunsaturated fatty acids | 3 | Weighted mode | 0.97718 | 0.95937-0.99531 | 0.13299 |
| prot-a-2609 | Reticulon-4 receptor | Polyunsaturated fatty acids | 3 | Inverse variance weighted | 0.97305 | 0.95369-0.99281 | 0.00772 |
| prot-a-2609 | Reticulon-4 receptor | Polyunsaturated fatty acids | 3 | MR Egger | 0.97429 | 0.90517-1.04869 | 0.61392 |
| prot-a-2609 | Reticulon-4 receptor | Polyunsaturated fatty acids | 3 | Weighted median | 0.97279 | 0.95193-0.9941 | 0.01259 |
| prot-a-2609 | Reticulon-4 receptor | Polyunsaturated fatty acids | 3 | Weighted mode | 0.96964 | 0.94496-0.99497 | 0.14382 |
| prot-a-2820 | Serine protease inhibitor Kazal-type 6 | Polyunsaturated fatty acids | 3 | Inverse variance weighted | 0.98659 | 0.97377-0.99958 | 0.04315 |
| prot-a-2820 | Serine protease inhibitor Kazal-type 6 | Polyunsaturated fatty acids | 3 | MR Egger | 0.9938 | 0.97547-1.01247 | 0.63073 |
| prot-a-2820 | Serine protease inhibitor Kazal-type 6 | Polyunsaturated fatty acids | 3 | Weighted median | 0.98795 | 0.97471-1.00137 | 0.07823 |
| prot-a-2820 | Serine protease inhibitor Kazal-type 6 | Polyunsaturated fatty acids | 3 | Weighted mode | 0.98843 | 0.97571-1.00132 | 0.22041 |
| prot-a-3109 | Thiosulfate sulfurtransferase | Polyunsaturated fatty acids | 3 | Inverse variance weighted | 0.9894 | 0.9798-0.9991 | 0.03221 |
| prot-a-3109 | Thiosulfate sulfurtransferase | Polyunsaturated fatty acids | 3 | MR Egger | 0.97871 | 0.95848-0.99937 | 0.29279 |
| prot-a-3109 | Thiosulfate sulfurtransferase | Polyunsaturated fatty acids | 3 | Weighted median | 0.98885 | 0.97892-0.99889 | 0.02951 |
| prot-a-3109 | Thiosulfate sulfurtransferase | Polyunsaturated fatty acids | 3 | Weighted mode | 0.9873 | 0.97726-0.99745 | 0.13401 |
| prot-a-3193 | Vascular cell adhesion protein 1 | Polyunsaturated fatty acids | 3 | Inverse variance weighted | 0.91766 | 0.85556-0.98427 | 0.01624 |
| prot-a-3193 | Vascular cell adhesion protein 1 | Polyunsaturated fatty acids | 3 | MR Egger | 0.97166 | 0.70947-1.33075 | 0.88714 |
| prot-a-3193 | Vascular cell adhesion protein 1 | Polyunsaturated fatty acids | 3 | Weighted median | 0.95116 | 0.90919-0.99506 | 0.02961 |
| prot-a-3193 | Vascular cell adhesion protein 1 | Polyunsaturated fatty acids | 3 | Weighted mode | 0.95908 | 0.91334-1.00713 | 0.23587 |
| prot-a-3203 | Vacuolar protein sorting-associated protein 29 | Polyunsaturated fatty acids | 3 | Inverse variance weighted | 0.90816 | 0.88252-0.93454 | 0 |
| prot-a-3203 | Vacuolar protein sorting-associated protein 29 | Polyunsaturated fatty acids | 3 | MR Egger | 0.89332 | 0.88378-0.90296 | 0.03088 |
| prot-a-3203 | Vacuolar protein sorting-associated protein 29 | Polyunsaturated fatty acids | 3 | Weighted median | 0.90586 | 0.89733-0.91446 | 0 |
| prot-a-3203 | Vacuolar protein sorting-associated protein 29 | Polyunsaturated fatty acids | 3 | Weighted mode | 0.90529 | 0.89684-0.91381 | 0.0023 |
| prot-a-3214 | Vitronectin | Polyunsaturated fatty acids | 3 | Inverse variance weighted | 0.98489 | 0.97489-0.99501 | 0.00349 |
| prot-a-3214 | Vitronectin | Polyunsaturated fatty acids | 3 | MR Egger | 0.97649 | 0.96129-0.99193 | 0.20664 |
| prot-a-3214 | Vitronectin | Polyunsaturated fatty acids | 3 | Weighted median | 0.98475 | 0.97716-0.9924 | 1.00E-04 |
| prot-a-3214 | Vitronectin | Polyunsaturated fatty acids | 3 | Weighted mode | 0.98339 | 0.97588-0.99095 | 0.05039 |
| prot-a-518 | Complement factor B | Polyunsaturated fatty acids | 4 | Inverse variance weighted | 0.97782 | 0.96185-0.99406 | 0.00761 |
| prot-a-518 | Complement factor B | Polyunsaturated fatty acids | 4 | MR Egger | 0.9908 | 0.96449-1.01783 | 0.57031 |
| prot-a-518 | Complement factor B | Polyunsaturated fatty acids | 4 | Weighted median | 0.98149 | 0.96436-0.99893 | 0.03762 |
| prot-a-518 | Complement factor B | Polyunsaturated fatty acids | 4 | Weighted mode | 0.98283 | 0.96387-1.00217 | 0.17985 |
| prot-a-527 | Human Chorionic Gonadotropin | Polyunsaturated fatty acids | 3 | Inverse variance weighted | 0.97924 | 0.96688-0.99176 | 0.00121 |
| prot-a-527 | Human Chorionic Gonadotropin | Polyunsaturated fatty acids | 3 | MR Egger | 0.9862 | 0.96872-1.00399 | 0.36976 |
| prot-a-527 | Human Chorionic Gonadotropin | Polyunsaturated fatty acids | 3 | Weighted median | 0.98048 | 0.96779-0.99333 | 0.003 |
| prot-a-527 | Human Chorionic Gonadotropin | Polyunsaturated fatty acids | 3 | Weighted mode | 0.98083 | 0.96847-0.99336 | 0.09607 |
| prot-a-760 | Cytohesin-4 | Polyunsaturated fatty acids | 3 | Inverse variance weighted | 0.96781 | 0.937-0.99965 | 0.04755 |
| prot-a-760 | Cytohesin-4 | Polyunsaturated fatty acids | 3 | MR Egger | 0.94792 | 0.83528-1.07575 | 0.55948 |
| prot-a-760 | Cytohesin-4 | Polyunsaturated fatty acids | 3 | Weighted median | 0.96421 | 0.92649-1.00347 | 0.07348 |
| prot-a-760 | Cytohesin-4 | Polyunsaturated fatty acids | 3 | Weighted mode | 0.96342 | 0.92168-1.00705 | 0.24092 |
| prot-b-18 | TNF receptor superfamily member 10b | Polyunsaturated fatty acids | 3 | Inverse variance weighted | 0.98745 | 0.97762-0.99738 | 0.01337 |
| prot-b-18 | TNF receptor superfamily member 10b | Polyunsaturated fatty acids | 3 | MR Egger | 0.98801 | 0.96752-1.00895 | 0.46194 |
| prot-b-18 | TNF receptor superfamily member 10b | Polyunsaturated fatty acids | 3 | Weighted median | 0.98756 | 0.97637-0.99889 | 0.03144 |
| prot-b-18 | TNF receptor superfamily member 10b | Polyunsaturated fatty acids | 3 | Weighted mode | 0.98886 | 0.97776-1.00009 | 0.19125 |
| prot-a-1027 | Protein FAM163A | Smoking | 3 | Inverse variance weighted | 1.00904 | 1.00151-1.01662 | 0.01846 |
| prot-a-1027 | Protein FAM163A | Smoking | 3 | MR Egger | 1.01807 | 0.99568-1.04096 | 0.35953 |
| prot-a-1027 | Protein FAM163A | Smoking | 3 | Weighted median | 1.01007 | 1.00202-1.01819 | 0.01408 |
| prot-a-1027 | Protein FAM163A | Smoking | 3 | Weighted mode | 1.01009 | 1.00108-1.01918 | 0.15922 |
| prot-a-1129 | Vascular endothelial growth factor receptor 3 | Smoking | 3 | Inverse variance weighted | 1.0059 | 1.00069-1.01114 | 0.02652 |
| prot-a-1129 | Vascular endothelial growth factor receptor 3 | Smoking | 3 | MR Egger | 1.00331 | 0.9923-1.01444 | 0.662 |
| prot-a-1129 | Vascular endothelial growth factor receptor 3 | Smoking | 3 | Weighted median | 1.00559 | 1.00031-1.0109 | 0.03797 |
| prot-a-1129 | Vascular endothelial growth factor receptor 3 | Smoking | 3 | Weighted mode | 1.0055 | 0.99943-1.0116 | 0.21789 |
| prot-a-1582 | Integrin alpha-5 | Smoking | 4 | Inverse variance weighted | 1.01217 | 1.00041-1.02407 | 0.04249 |
| prot-a-1582 | Integrin alpha-5 | Smoking | 4 | MR Egger | 1.01385 | 0.97167-1.05786 | 0.59073 |
| prot-a-1582 | Integrin alpha-5 | Smoking | 4 | Weighted median | 1.00827 | 0.99391-1.02284 | 0.2604 |
| prot-a-1582 | Integrin alpha-5 | Smoking | 4 | Weighted mode | 1.00632 | 0.98898-1.02396 | 0.52886 |
| prot-a-2302 | Plasmin | Smoking | 3 | Inverse variance weighted | 1.0141 | 1.00108-1.0273 | 0.03373 |
| prot-a-2302 | Plasmin | Smoking | 3 | MR Egger | 1.02035 | 0.97638-1.06629 | 0.5348 |
| prot-a-2302 | Plasmin | Smoking | 3 | Weighted median | 1.01551 | 0.99891-1.0324 | 0.06721 |
| prot-a-2302 | Plasmin | Smoking | 3 | Weighted mode | 1.01881 | 0.99737-1.04071 | 0.22799 |
| prot-a-2309 | Plexin-C1 | Smoking | 5 | Inverse variance weighted | 1.00554 | 1.00116-1.00994 | 0.01306 |
| prot-a-2309 | Plexin-C1 | Smoking | 5 | MR Egger | 1.00838 | 1.00004-1.0168 | 0.14356 |
| prot-a-2309 | Plexin-C1 | Smoking | 5 | Weighted median | 1.00609 | 1.00118-1.01103 | 0.01503 |
| prot-a-2309 | Plexin-C1 | Smoking | 5 | Weighted mode | 1.0064 | 1.00142-1.0114 | 0.06525 |
| prot-a-2357 | PR domain zinc finger protein 1 | Smoking | 3 | Inverse variance weighted | 1.01147 | 1.00458-1.01841 | 0.00107 |
| prot-a-2357 | PR domain zinc finger protein 1 | Smoking | 3 | MR Egger | 1.03332 | 0.91908-1.16177 | 0.6807 |
| prot-a-2357 | PR domain zinc finger protein 1 | Smoking | 3 | Weighted median | 1.01088 | 1.00535-1.01644 | 0.00011 |
| prot-a-2357 | PR domain zinc finger protein 1 | Smoking | 3 | Weighted mode | 1.011 | 1.00462-1.01741 | 0.0771 |
| prot-a-2760 | Sodium-coupled monocarboxylate transporter 1 | Smoking | 3 | Inverse variance weighted | 1.0142 | 1.00337-1.02515 | 0.01003 |
| prot-a-2760 | Sodium-coupled monocarboxylate transporter 1 | Smoking | 3 | MR Egger | 1.01052 | 0.95151-1.07318 | 0.79091 |
| prot-a-2760 | Sodium-coupled monocarboxylate transporter 1 | Smoking | 3 | Weighted median | 1.01406 | 1.00203-1.02622 | 0.02179 |
| prot-a-2760 | Sodium-coupled monocarboxylate transporter 1 | Smoking | 3 | Weighted mode | 1.0138 | 0.99995-1.02784 | 0.19004 |
| prot-a-2892 | Estrogen sulfotransferase | Smoking | 3 | Inverse variance weighted | 1.00983 | 1.00301-1.01668 | 0.00464 |
| prot-a-2892 | Estrogen sulfotransferase | Smoking | 3 | MR Egger | 1.01291 | 0.99409-1.03209 | 0.40803 |
| prot-a-2892 | Estrogen sulfotransferase | Smoking | 3 | Weighted median | 1.00977 | 1.00187-1.01772 | 0.01525 |
| prot-a-2892 | Estrogen sulfotransferase | Smoking | 3 | Weighted mode | 1.00897 | 1.00081-1.0172 | 0.16403 |
| prot-a-387 | Eotaxin | Smoking | 3 | Inverse variance weighted | 1.0115 | 1.00301-1.02006 | 0.00784 |
| prot-a-387 | Eotaxin | Smoking | 3 | MR Egger | 1.01737 | 0.9963-1.03889 | 0.35332 |
| prot-a-387 | Eotaxin | Smoking | 3 | Weighted median | 1.01235 | 1.00336-1.02143 | 0.007 |
| prot-a-387 | Eotaxin | Smoking | 3 | Weighted mode | 1.01291 | 1.0028-1.02312 | 0.12913 |
| prot-a-390 | C-C motif chemokine 14 | Smoking | 3 | Inverse variance weighted | 1.00627 | 1.00018-1.0124 | 0.04345 |
| prot-a-390 | C-C motif chemokine 14 | Smoking | 3 | MR Egger | 1.00256 | 0.99189-1.01336 | 0.72094 |
| prot-a-390 | C-C motif chemokine 14 | Smoking | 3 | Weighted median | 1.00582 | 0.99931-1.01237 | 0.07991 |
| prot-a-390 | C-C motif chemokine 14 | Smoking | 3 | Weighted mode | 1.00414 | 0.99757-1.01076 | 0.34256 |
| prot-a-794 | Beta-defensin 119 | Smoking | 7 | Inverse variance weighted | 1.01135 | 1.00532-1.01741 | 0.00022 |
| prot-a-794 | Beta-defensin 119 | Smoking | 7 | MR Egger | 1.00719 | 0.99274-1.02185 | 0.37595 |
| prot-a-794 | Beta-defensin 119 | Smoking | 7 | Weighted median | 1.01419 | 1.0068-1.02164 | 0.00016 |
| prot-a-794 | Beta-defensin 119 | Smoking | 7 | Weighted mode | 1.01509 | 1.00639-1.02387 | 0.01433 |
| prot-a-1389 | Oxidoreductase HTATIP2 | Smoking | 3 | Inverse variance weighted | 0.9862 | 0.97554-0.99699 | 0.01229 |
| prot-a-1389 | Oxidoreductase HTATIP2 | Smoking | 3 | MR Egger | 0.99239 | 0.966-1.0195 | 0.67715 |
| prot-a-1389 | Oxidoreductase HTATIP2 | Smoking | 3 | Weighted median | 0.98478 | 0.97265-0.99707 | 0.01533 |
| prot-a-1389 | Oxidoreductase HTATIP2 | Smoking | 3 | Weighted mode | 0.98421 | 0.97055-0.99806 | 0.15528 |
| prot-a-1414 | Gamma-interferon-inducible protein 16 | Smoking | 3 | Inverse variance weighted | 0.99441 | 0.98947-0.99937 | 0.02733 |
| prot-a-1414 | Gamma-interferon-inducible protein 16 | Smoking | 3 | MR Egger | 0.98673 | 0.97225-1.00142 | 0.3272 |
| prot-a-1414 | Gamma-interferon-inducible protein 16 | Smoking | 3 | Weighted median | 0.99382 | 0.9888-0.99886 | 0.01638 |
| prot-a-1414 | Gamma-interferon-inducible protein 16 | Smoking | 3 | Weighted mode | 0.99359 | 0.98812-0.9991 | 0.15015 |
| prot-a-1586 | Inter-alpha-trypsin inhibitor heavy chain H1 | Smoking | 3 | Inverse variance weighted | 0.99254 | 0.98596-0.99916 | 0.02726 |
| prot-a-1586 | Inter-alpha-trypsin inhibitor heavy chain H1 | Smoking | 3 | MR Egger | 0.98487 | 0.96202-1.00827 | 0.42405 |
| prot-a-1586 | Inter-alpha-trypsin inhibitor heavy chain H1 | Smoking | 3 | Weighted median | 0.99295 | 0.98776-0.99817 | 0.00818 |
| prot-a-1586 | Inter-alpha-trypsin inhibitor heavy chain H1 | Smoking | 3 | Weighted mode | 0.98916 | 0.98321-0.99515 | 0.07134 |
| prot-a-1879 | Protein MENT | Smoking | 3 | Inverse variance weighted | 0.98775 | 0.9786-0.99698 | 0.0094 |
| prot-a-1879 | Protein MENT | Smoking | 3 | MR Egger | 0.98555 | 0.95546-1.01658 | 0.5264 |
| prot-a-1879 | Protein MENT | Smoking | 3 | Weighted median | 0.98735 | 0.97721-0.99758 | 0.01553 |
| prot-a-1879 | Protein MENT | Smoking | 3 | Weighted mode | 0.98644 | 0.97504-0.99797 | 0.14796 |
| prot-a-1995 | N-acetyl-D-glucosamine kinase | Smoking | 3 | Inverse variance weighted | 0.99402 | 0.98848-0.9996 | 0.0356 |
| prot-a-1995 | N-acetyl-D-glucosamine kinase | Smoking | 3 | MR Egger | 0.99584 | 0.97699-1.01506 | 0.74297 |
| prot-a-1995 | N-acetyl-D-glucosamine kinase | Smoking | 3 | Weighted median | 0.9938 | 0.98729-1.00036 | 0.06395 |
| prot-a-1995 | N-acetyl-D-glucosamine kinase | Smoking | 3 | Weighted mode | 0.9919 | 0.98441-0.99945 | 0.17017 |
| prot-a-2043 | Tumor necrosis factor receptor superfamily member 16 | Smoking | 4 | Inverse variance weighted | 0.99041 | 0.98135-0.99956 | 0.03993 |
| prot-a-2043 | Tumor necrosis factor receptor superfamily member 16 | Smoking | 4 | MR Egger | 0.95325 | 0.79985-1.13607 | 0.64626 |
| prot-a-2043 | Tumor necrosis factor receptor superfamily member 16 | Smoking | 4 | Weighted median | 0.9908 | 0.98093-1.00077 | 0.07042 |
| prot-a-2043 | Tumor necrosis factor receptor superfamily member 16 | Smoking | 4 | Weighted mode | 0.99671 | 0.97956-1.01417 | 0.73486 |
| prot-a-2082 | Neuropeptide W | Smoking | 3 | Inverse variance weighted | 0.98755 | 0.97997-0.99518 | 0.00143 |
| prot-a-2082 | Neuropeptide W | Smoking | 3 | MR Egger | 0.98264 | 0.95547-1.01058 | 0.43607 |
| prot-a-2082 | Neuropeptide W | Smoking | 3 | Weighted median | 0.98664 | 0.97812-0.99524 | 0.00238 |
| prot-a-2082 | Neuropeptide W | Smoking | 3 | Weighted mode | 0.98497 | 0.97604-0.99399 | 0.08272 |
| prot-a-2172 | Peptidyl-glycine alpha-amidating monooxygenase | Smoking | 6 | Inverse variance weighted | 0.99358 | 0.98791-0.99928 | 0.02726 |
| prot-a-2172 | Peptidyl-glycine alpha-amidating monooxygenase | Smoking | 6 | MR Egger | 0.99581 | 0.98257-1.00924 | 0.57243 |
| prot-a-2172 | Peptidyl-glycine alpha-amidating monooxygenase | Smoking | 6 | Weighted median | 0.99414 | 0.98727-1.00105 | 0.09653 |
| prot-a-2172 | Peptidyl-glycine alpha-amidating monooxygenase | Smoking | 6 | Weighted mode | 0.99429 | 0.9863-1.00235 | 0.22314 |
| prot-a-2908 | Switch-associated protein 70 | Smoking | 3 | Inverse variance weighted | 0.99354 | 0.98775-0.99936 | 0.02975 |
| prot-a-2908 | Switch-associated protein 70 | Smoking | 3 | MR Egger | 0.99427 | 0.98217-1.00651 | 0.52615 |
| prot-a-2908 | Switch-associated protein 70 | Smoking | 3 | Weighted median | 0.99371 | 0.98747-0.99998 | 0.04941 |
| prot-a-2908 | Switch-associated protein 70 | Smoking | 3 | Weighted mode | 0.99365 | 0.98684-1.0005 | 0.21071 |
| prot-a-305 | Complement C1q tumor necrosis factor-related protein 5 | Smoking | 4 | Inverse variance weighted | 0.99049 | 0.98274-0.9983 | 0.01706 |
| prot-a-305 | Complement C1q tumor necrosis factor-related protein 5 | Smoking | 4 | MR Egger | 0.97433 | 0.938-1.01206 | 0.31177 |
| prot-a-305 | Complement C1q tumor necrosis factor-related protein 5 | Smoking | 4 | Weighted median | 0.98907 | 0.97978-0.99844 | 0.02237 |
| prot-a-305 | Complement C1q tumor necrosis factor-related protein 5 | Smoking | 4 | Weighted mode | 0.98766 | 0.97674-0.9987 | 0.11636 |
| prot-a-419 | Scavenger receptor cysteine-rich type 1 protein M130 | Smoking | 3 | Inverse variance weighted | 0.98724 | 0.97873-0.99582 | 0.00362 |
| prot-a-419 | Scavenger receptor cysteine-rich type 1 protein M130 | Smoking | 3 | MR Egger | 0.99132 | 0.97461-1.00831 | 0.49815 |
| prot-a-419 | Scavenger receptor cysteine-rich type 1 protein M130 | Smoking | 3 | Weighted median | 0.98861 | 0.97942-0.99789 | 0.0163 |
| prot-a-419 | Scavenger receptor cysteine-rich type 1 protein M130 | Smoking | 3 | Weighted mode | 0.98863 | 0.97816-0.99921 | 0.16987 |
| prot-a-654 | Cyclic AMP-responsive element-binding protein 3-like protein 4 | Smoking | 3 | Inverse variance weighted | 0.99099 | 0.98511-0.99691 | 0.00288 |
| prot-a-654 | Cyclic AMP-responsive element-binding protein 3-like protein 4 | Smoking | 3 | MR Egger | 0.99329 | 0.9837-1.00297 | 0.40328 |
| prot-a-654 | Cyclic AMP-responsive element-binding protein 3-like protein 4 | Smoking | 3 | Weighted median | 0.99125 | 0.98486-0.99768 | 0.00774 |
| prot-a-654 | Cyclic AMP-responsive element-binding protein 3-like protein 4 | Smoking | 3 | Weighted mode | 0.99149 | 0.98475-0.99829 | 0.13373 |
| prot-a-657 | Cysteine-rich with EGF-like domain protein 1 | Smoking | 4 | Inverse variance weighted | 0.99549 | 0.99212-0.99888 | 0.00908 |
| prot-a-657 | Cysteine-rich with EGF-like domain protein 1 | Smoking | 4 | MR Egger | 0.99838 | 0.98716-1.00974 | 0.80565 |
| prot-a-657 | Cysteine-rich with EGF-like domain protein 1 | Smoking | 4 | Weighted median | 0.99564 | 0.99219-0.9991 | 0.01348 |
| prot-a-657 | Cysteine-rich with EGF-like domain protein 1 | Smoking | 4 | Weighted mode | 0.99582 | 0.99254-0.99912 | 0.08911 |
| prot-a-710 | Cardiotrophin-1 | Smoking | 3 | Inverse variance weighted | 0.99472 | 0.99036-0.99911 | 0.0184 |
| prot-a-710 | Cardiotrophin-1 | Smoking | 3 | MR Egger | 0.96559 | 0.91609-1.01777 | 0.4165 |
| prot-a-710 | Cardiotrophin-1 | Smoking | 3 | Weighted median | 0.99393 | 0.98934-0.99854 | 0.00992 |
| prot-a-710 | Cardiotrophin-1 | Smoking | 3 | Weighted mode | 0.99386 | 0.98897-0.99879 | 0.13468 |
| prot-a-722 | Cathepsin F | Smoking | 3 | Inverse variance weighted | 0.98738 | 0.9751-0.99981 | 0.04665 |
| prot-a-722 | Cathepsin F | Smoking | 3 | MR Egger | 0.99389 | 0.91648-1.07785 | 0.90642 |
| prot-a-722 | Cathepsin F | Smoking | 3 | Weighted median | 0.99264 | 0.98116-1.00424 | 0.21276 |
| prot-a-722 | Cathepsin F | Smoking | 3 | Weighted mode | 0.99382 | 0.98011-1.00773 | 0.47422 |
| prot-a-819 | Probable dimethyladenosine transferase | Smoking | 3 | Inverse variance weighted | 0.98863 | 0.98169-0.99562 | 0.00147 |
| prot-a-819 | Probable dimethyladenosine transferase | Smoking | 3 | MR Egger | 0.99119 | 0.97759-1.00499 | 0.42843 |
| prot-a-819 | Probable dimethyladenosine transferase | Smoking | 3 | Weighted median | 0.9889 | 0.98147-0.99638 | 0.00371 |
| prot-a-819 | Probable dimethyladenosine transferase | Smoking | 3 | Weighted mode | 0.98919 | 0.9817-0.99674 | 0.10727 |
| prot-a-10 | Histo-blood group ABO system transferase | LDL cholesterol | 3 | Inverse variance weighted | 1.03208 | 1.01145-1.05312 | 0.00217 |
| prot-a-10 | Histo-blood group ABO system transferase | LDL cholesterol | 3 | MR Egger | 1.01052 | 0.98765-1.03392 | 0.53485 |
| prot-a-10 | Histo-blood group ABO system transferase | LDL cholesterol | 3 | Weighted median | 1.03067 | 1.02211-1.0393 | 0.00000 |
| prot-a-10 | Histo-blood group ABO system transferase | LDL cholesterol | 3 | Weighted mode | 1.02908 | 1.02042-1.0378 | 0.02186 |
| prot-a-1046 | Glycosaminoglycan xylosylkinase | LDL cholesterol | 3 | Inverse variance weighted | 1.18897 | 1.03035-1.37201 | 0.01783 |
| prot-a-1046 | Glycosaminoglycan xylosylkinase | LDL cholesterol | 3 | MR Egger | 1.76097 | 1.48797-2.08406 | 0.09596 |
| prot-a-1046 | Glycosaminoglycan xylosylkinase | LDL cholesterol | 3 | Weighted median | 1.06906 | 1.00045-1.14239 | 0.04847 |
| prot-a-1046 | Glycosaminoglycan xylosylkinase | LDL cholesterol | 3 | Weighted mode | 1.06451 | 1.00532-1.12719 | 0.16552 |
| prot-a-1115 | Inactive peptidyl-prolyl cis-trans isomerase FKBP6 | LDL cholesterol | 3 | Inverse variance weighted | 1.01765 | 1.00821-1.02718 | 0.00023 |
| prot-a-1115 | Inactive peptidyl-prolyl cis-trans isomerase FKBP6 | LDL cholesterol | 3 | MR Egger | 1.01245 | 0.99505-1.03016 | 0.39502 |
| prot-a-1115 | Inactive peptidyl-prolyl cis-trans isomerase FKBP6 | LDL cholesterol | 3 | Weighted median | 1.01709 | 1.00775-1.02652 | 0.00032 |
| prot-a-1115 | Inactive peptidyl-prolyl cis-trans isomerase FKBP6 | LDL cholesterol | 3 | Weighted mode | 1.01648 | 1.00646-1.0266 | 0.08377 |
| prot-a-1148 | Ferritin | LDL cholesterol | 3 | Inverse variance weighted | 0.91500 | 0.83798-0.9991 | 0.04771 |
| prot-a-1148 | Ferritin | LDL cholesterol | 3 | MR Egger | 0.84586 | 0.71037-1.0072 | 0.31129 |
| prot-a-1148 | Ferritin | LDL cholesterol | 3 | Weighted median | 0.89455 | 0.8722-0.91748 | 0.00000 |
| prot-a-1148 | Ferritin | LDL cholesterol | 3 | Weighted mode | 0.89433 | 0.87245-0.91677 | 0.01257 |
| prot-a-1277 | Granulins | LDL cholesterol | 4 | Inverse variance weighted | 1.12856 | 1.03549-1.23001 | 0.00589 |
| prot-a-1277 | Granulins | LDL cholesterol | 4 | MR Egger | 1.27570 | 1.22886-1.32432 | 0.00609 |
| prot-a-1277 | Granulins | LDL cholesterol | 4 | Weighted median | 1.10608 | 1.08614-1.12639 | 0.00000 |
| prot-a-1277 | Granulins | LDL cholesterol | 4 | Weighted mode | 1.17300 | 1.15293-1.19343 | 0.00037 |
| prot-a-1369 | Haptoglobin | LDL cholesterol | 5 | Inverse variance weighted | 0.93851 | 0.92712-0.95004 | 0.00000 |
| prot-a-1369 | Haptoglobin | LDL cholesterol | 5 | MR Egger | 0.92924 | 0.90184-0.95747 | 0.01714 |
| prot-a-1369 | Haptoglobin | LDL cholesterol | 5 | Weighted median | 0.93694 | 0.9242-0.94986 | 0.00000 |
| prot-a-1369 | Haptoglobin | LDL cholesterol | 5 | Weighted mode | 0.93527 | 0.9227-0.94801 | 0.00063 |
| prot-a-1622 | Vascular endothelial growth factor receptor 2 | LDL cholesterol | 4 | Inverse variance weighted | 0.93669 | 0.87945-0.99766 | 0.04207 |
| prot-a-1622 | Vascular endothelial growth factor receptor 2 | LDL cholesterol | 4 | MR Egger | 0.91597 | 0.79449-1.05603 | 0.35020 |
| prot-a-1622 | Vascular endothelial growth factor receptor 2 | LDL cholesterol | 4 | Weighted median | 0.95445 | 0.92861-0.98102 | 0.00087 |
| prot-a-1622 | Vascular endothelial growth factor receptor 2 | LDL cholesterol | 4 | Weighted mode | 0.97844 | 0.95339-1.00413 | 0.19790 |
| prot-a-1645 | Killer cell immunoglobulin-like receptor 2DS2 | LDL cholesterol | 4 | Inverse variance weighted | 0.96076 | 0.92802-0.99465 | 0.02362 |
| prot-a-1645 | Killer cell immunoglobulin-like receptor 2DS2 | LDL cholesterol | 4 | MR Egger | 0.90399 | 0.74538-1.09634 | 0.41296 |
| prot-a-1645 | Killer cell immunoglobulin-like receptor 2DS2 | LDL cholesterol | 4 | Weighted median | 0.96269 | 0.92441-1.00255 | 0.06623 |
| prot-a-1645 | Killer cell immunoglobulin-like receptor 2DS2 | LDL cholesterol | 4 | Weighted mode | 0.97922 | 0.92315-1.03869 | 0.53533 |
| prot-a-1902 | MICOS complex subunit MIC10 | LDL cholesterol | 3 | Inverse variance weighted | 1.01531 | 1.00673-1.02397 | 0.00045 |
| prot-a-1902 | MICOS complex subunit MIC10 | LDL cholesterol | 3 | MR Egger | 1.01475 | 0.99789-1.03189 | 0.33648 |
| prot-a-1902 | MICOS complex subunit MIC10 | LDL cholesterol | 3 | Weighted median | 1.01483 | 1.00582-1.02392 | 0.00121 |
| prot-a-1902 | MICOS complex subunit MIC10 | LDL cholesterol | 3 | Weighted mode | 1.01475 | 1.00537-1.02421 | 0.09074 |
| prot-a-2039 | Neurofascin | LDL cholesterol | 3 | Inverse variance weighted | 1.01689 | 1.00018-1.03389 | 0.04756 |
| prot-a-2039 | Neurofascin | LDL cholesterol | 3 | MR Egger | 1.00043 | 0.94585-1.05815 | 0.99052 |
| prot-a-2039 | Neurofascin | LDL cholesterol | 3 | Weighted median | 1.01481 | 0.99681-1.03313 | 0.10742 |
| prot-a-2039 | Neurofascin | LDL cholesterol | 3 | Weighted mode | 1.01467 | 0.99583-1.03386 | 0.26727 |
| prot-a-2129 | ADP-ribose pyrophosphatase, mitochondrial | LDL cholesterol | 3 | Inverse variance weighted | 1.05489 | 1.0229-1.08789 | 0.00067 |
| prot-a-2129 | ADP-ribose pyrophosphatase, mitochondrial | LDL cholesterol | 3 | MR Egger | 1.09456 | 1.03304-1.15975 | 0.20100 |
| prot-a-2129 | ADP-ribose pyrophosphatase, mitochondrial | LDL cholesterol | 3 | Weighted median | 1.05458 | 1.02526-1.08475 | 0.00022 |
| prot-a-2129 | ADP-ribose pyrophosphatase, mitochondrial | LDL cholesterol | 3 | Weighted mode | 1.06324 | 1.02501-1.10289 | 0.08161 |
| prot-a-220 | Beta-1,4-galactosyltransferase 6 | LDL cholesterol | 3 | Inverse variance weighted | 1.02632 | 1.00283-1.05037 | 0.02788 |
| prot-a-220 | Beta-1,4-galactosyltransferase 6 | LDL cholesterol | 3 | MR Egger | 1.02406 | 0.99275-1.05636 | 0.37416 |
| prot-a-220 | Beta-1,4-galactosyltransferase 6 | LDL cholesterol | 3 | Weighted median | 1.02635 | 1.00203-1.05127 | 0.03356 |
| prot-a-220 | Beta-1,4-galactosyltransferase 6 | LDL cholesterol | 3 | Weighted mode | 1.02656 | 0.99969-1.05416 | 0.19230 |
| prot-a-2362 | Proteoglycan 3 | LDL cholesterol | 3 | Inverse variance weighted | 1.04740 | 1.00935-1.08688 | 0.01417 |
| prot-a-2362 | Proteoglycan 3 | LDL cholesterol | 3 | MR Egger | 1.04915 | 0.97878-1.12458 | 0.40488 |
| prot-a-2362 | Proteoglycan 3 | LDL cholesterol | 3 | Weighted median | 1.04482 | 0.99753-1.09436 | 0.06356 |
| prot-a-2362 | Proteoglycan 3 | LDL cholesterol | 3 | Weighted mode | 1.04286 | 0.98999-1.09855 | 0.25468 |
| prot-a-2573 | E3 ubiquitin-protein ligase RNF8 | LDL cholesterol | 3 | Inverse variance weighted | 1.04068 | 1.01036-1.07192 | 0.00822 |
| prot-a-2573 | E3 ubiquitin-protein ligase RNF8 | LDL cholesterol | 3 | MR Egger | 1.04022 | 0.87692-1.23392 | 0.72945 |
| prot-a-2573 | E3 ubiquitin-protein ligase RNF8 | LDL cholesterol | 3 | Weighted median | 1.04371 | 1.01543-1.07278 | 0.00227 |
| prot-a-2573 | E3 ubiquitin-protein ligase RNF8 | LDL cholesterol | 3 | Weighted mode | 1.04991 | 1.01706-1.08381 | 0.09531 |
| prot-a-2575 | rRNA methyltransferase 3, mitochondrial | LDL cholesterol | 3 | Inverse variance weighted | 1.02497 | 1.01024-1.03991 | 0.00084 |
| prot-a-2575 | rRNA methyltransferase 3, mitochondrial | LDL cholesterol | 3 | MR Egger | 1.02383 | 0.99354-1.05505 | 0.36725 |
| prot-a-2575 | rRNA methyltransferase 3, mitochondrial | LDL cholesterol | 3 | Weighted median | 1.02628 | 1.01088-1.04192 | 0.00077 |
| prot-a-2575 | rRNA methyltransferase 3, mitochondrial | LDL cholesterol | 3 | Weighted mode | 1.02650 | 1.01021-1.04306 | 0.08515 |
| prot-a-2744 | SLAM family member 7 | LDL cholesterol | 3 | Inverse variance weighted | 1.03419 | 1.00879-1.06023 | 0.00806 |
| prot-a-2744 | SLAM family member 7 | LDL cholesterol | 3 | MR Egger | 1.03047 | 0.91427-1.16144 | 0.70906 |
| prot-a-2744 | SLAM family member 7 | LDL cholesterol | 3 | Weighted median | 1.03760 | 1.01413-1.06161 | 0.00157 |
| prot-a-2744 | SLAM family member 7 | LDL cholesterol | 3 | Weighted mode | 1.04183 | 1.01549-1.06886 | 0.08839 |
| prot-a-303 | Complement C1q tumor necrosis factor-related protein 1 | LDL cholesterol | 5 | Inverse variance weighted | 1.14323 | 1.02349-1.27698 | 0.01772 |
| prot-a-303 | Complement C1q tumor necrosis factor-related protein 1 | LDL cholesterol | 5 | MR Egger | 1.34662 | 1.15778-1.56625 | 0.03072 |
| prot-a-303 | Complement C1q tumor necrosis factor-related protein 1 | LDL cholesterol | 5 | Weighted median | 1.06046 | 1.02725-1.09474 | 0.00030 |
| prot-a-303 | Complement C1q tumor necrosis factor-related protein 1 | LDL cholesterol | 5 | Weighted mode | 1.22823 | 1.19763-1.25961 | 0.00009 |
| prot-a-3124 | Thioredoxin domain-containing protein 5 | LDL cholesterol | 3 | Inverse variance weighted | 1.04104 | 1.01293-1.06993 | 0.00398 |
| prot-a-3124 | Thioredoxin domain-containing protein 5 | LDL cholesterol | 3 | MR Egger | 1.09653 | 1.00553-1.19576 | 0.28473 |
| prot-a-3124 | Thioredoxin domain-containing protein 5 | LDL cholesterol | 3 | Weighted median | 1.04184 | 1.01117-1.07344 | 0.00717 |
| prot-a-3124 | Thioredoxin domain-containing protein 5 | LDL cholesterol | 3 | Weighted mode | 1.02534 | 0.99067-1.06123 | 0.28995 |
| prot-a-346 | Calcium/calmodulin-dependent protein kinase type 1 | LDL cholesterol | 3 | Inverse variance weighted | 1.01426 | 1.00405-1.02458 | 0.00607 |
| prot-a-346 | Calcium/calmodulin-dependent protein kinase type 1 | LDL cholesterol | 3 | MR Egger | 1.02725 | 0.96714-1.0911 | 0.54275 |
| prot-a-346 | Calcium/calmodulin-dependent protein kinase type 1 | LDL cholesterol | 3 | Weighted median | 1.01424 | 1.00387-1.02472 | 0.00701 |
| prot-a-346 | Calcium/calmodulin-dependent protein kinase type 1 | LDL cholesterol | 3 | Weighted mode | 1.01498 | 1.00398-1.02611 | 0.11605 |
| prot-a-347 | Calcium/calmodulin-dependent protein kinase type 1D | LDL cholesterol | 3 | Inverse variance weighted | 1.04036 | 1.00543-1.07651 | 0.02317 |
| prot-a-347 | Calcium/calmodulin-dependent protein kinase type 1D | LDL cholesterol | 3 | MR Egger | 1.01365 | 0.87439-1.1751 | 0.88674 |
| prot-a-347 | Calcium/calmodulin-dependent protein kinase type 1D | LDL cholesterol | 3 | Weighted median | 1.04533 | 1.0148-1.07679 | 0.00338 |
| prot-a-347 | Calcium/calmodulin-dependent protein kinase type 1D | LDL cholesterol | 3 | Weighted mode | 1.05262 | 1.01845-1.08793 | 0.09301 |
| prot-a-402 | C-C motif chemokine 25 | LDL cholesterol | 3 | Inverse variance weighted | 0.86013 | 0.75542-0.97935 | 0.02290 |
| prot-a-402 | C-C motif chemokine 25 | LDL cholesterol | 3 | MR Egger | 0.00305 | 0-117.86766 | 0.47703 |
| prot-a-402 | C-C motif chemokine 25 | LDL cholesterol | 3 | Weighted median | 0.89772 | 0.85647-0.94096 | 0.00001 |
| prot-a-402 | C-C motif chemokine 25 | LDL cholesterol | 3 | Weighted mode | 0.91745 | 0.87253-0.96468 | 0.07814 |
| prot-a-426 | CD209 antigen | LDL cholesterol | 3 | Inverse variance weighted | 1.03931 | 1.01553-1.06366 | 0.00110 |
| prot-a-426 | CD209 antigen | LDL cholesterol | 3 | MR Egger | 1.06056 | 1.00327-1.12113 | 0.28589 |
| prot-a-426 | CD209 antigen | LDL cholesterol | 3 | Weighted median | 1.04442 | 1.03106-1.05796 | 0.00000 |
| prot-a-426 | CD209 antigen | LDL cholesterol | 3 | Weighted mode | 1.04711 | 1.03304-1.06137 | 0.02174 |
| prot-a-44 | Adhesion G protein-coupled receptor F5 | LDL cholesterol | 5 | Inverse variance weighted | 0.95273 | 0.91398-0.99311 | 0.02223 |
| prot-a-44 | Adhesion G protein-coupled receptor F5 | LDL cholesterol | 5 | MR Egger | 0.95987 | 0.8937-1.03093 | 0.34284 |
| prot-a-44 | Adhesion G protein-coupled receptor F5 | LDL cholesterol | 5 | Weighted median | 0.96499 | 0.93133-0.99986 | 0.04913 |
| prot-a-44 | Adhesion G protein-coupled receptor F5 | LDL cholesterol | 5 | Weighted mode | 0.99575 | 0.96662-1.02575 | 0.79243 |
| prot-a-586 | Dual specificity protein kinase CLK2 | LDL cholesterol | 3 | Inverse variance weighted | 1.03607 | 1.01406-1.05855 | 0.00122 |
| prot-a-586 | Dual specificity protein kinase CLK2 | LDL cholesterol | 3 | MR Egger | 1.04153 | 0.96405-1.12525 | 0.49007 |
| prot-a-586 | Dual specificity protein kinase CLK2 | LDL cholesterol | 3 | Weighted median | 1.03937 | 1.01582-1.06347 | 0.00096 |
| prot-a-586 | Dual specificity protein kinase CLK2 | LDL cholesterol | 3 | Weighted mode | 1.04103 | 1.01782-1.06476 | 0.07298 |
| prot-a-705 | Cystatin-F | LDL cholesterol | 3 | Inverse variance weighted | 0.98277 | 0.96956-0.99617 | 0.01187 |
| prot-a-705 | Cystatin-F | LDL cholesterol | 3 | MR Egger | 0.98959 | 0.96475-1.01506 | 0.56772 |
| prot-a-705 | Cystatin-F | LDL cholesterol | 3 | Weighted median | 0.98390 | 0.96981-0.99819 | 0.02737 |
| prot-a-705 | Cystatin-F | LDL cholesterol | 3 | Weighted mode | 0.98480 | 0.96949-1.00034 | 0.19522 |
| prot-a-710 | Cardiotrophin-1 | LDL cholesterol | 3 | Inverse variance weighted | 1.36919 | 1.22401-1.5316 | 0.00000 |
| prot-a-710 | Cardiotrophin-1 | LDL cholesterol | 3 | MR Egger | 1.69347 | 0.27466-10.44149 | 0.67134 |
| prot-a-710 | Cardiotrophin-1 | LDL cholesterol | 3 | Weighted median | 1.33237 | 1.299-1.3666 | 0.00000 |
| prot-a-710 | Cardiotrophin-1 | LDL cholesterol | 3 | Weighted mode | 1.31308 | 1.2784-1.3487 | 0.00250 |
| prot-a-842 | DnaJ homolog subfamily B member 9 | LDL cholesterol | 4 | Inverse variance weighted | 1.05649 | 1.01268-1.10221 | 0.01100 |
| prot-a-842 | DnaJ homolog subfamily B member 9 | LDL cholesterol | 4 | MR Egger | 1.10941 | 0.94614-1.30085 | 0.32944 |
| prot-a-842 | DnaJ homolog subfamily B member 9 | LDL cholesterol | 4 | Weighted median | 1.05632 | 1.02841-1.08499 | 0.00006 |
| prot-a-842 | DnaJ homolog subfamily B member 9 | LDL cholesterol | 4 | Weighted mode | 1.05801 | 1.02887-1.08797 | 0.02880 |
| prot-b-25 | matrix metallopeptidase 3 | LDL cholesterol | 3 | Inverse variance weighted | 0.98625 | 0.9776-0.99497 | 0.00205 |
| prot-b-25 | matrix metallopeptidase 3 | LDL cholesterol | 3 | MR Egger | 0.98601 | 0.97605-0.99608 | 0.22438 |
| prot-b-25 | matrix metallopeptidase 3 | LDL cholesterol | 3 | Weighted median | 0.98659 | 0.97624-0.99705 | 0.01209 |
| prot-b-25 | matrix metallopeptidase 3 | LDL cholesterol | 3 | Weighted mode | 0.98664 | 0.97583-0.99757 | 0.13917 |

**Figure S1.** Indirect effect of plasma proteins on CRC via risk factors. Indirect effect of SLC5A8 on CRC through smoking. β_EM_, effects of exposure on mediator; β_MO_, effects of mediator on outcome; β_EO_, effects of exposure on outcome.


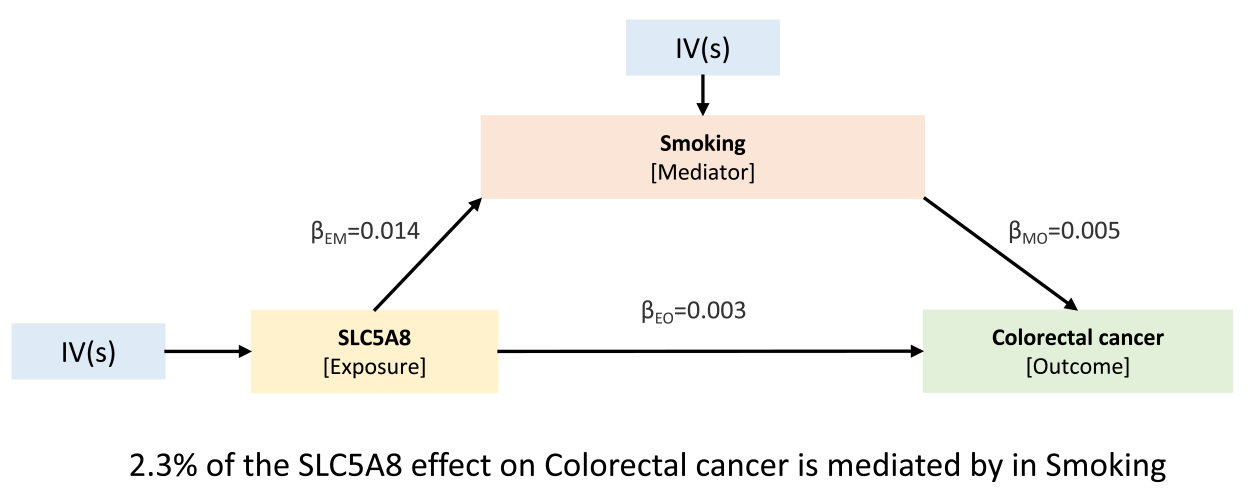


**Figure S2.** Indirect effect of plasma proteins on CRC via risk factors. Indirect effect of MICB on CRC through cholesterol to total lipids ratio in IDL.


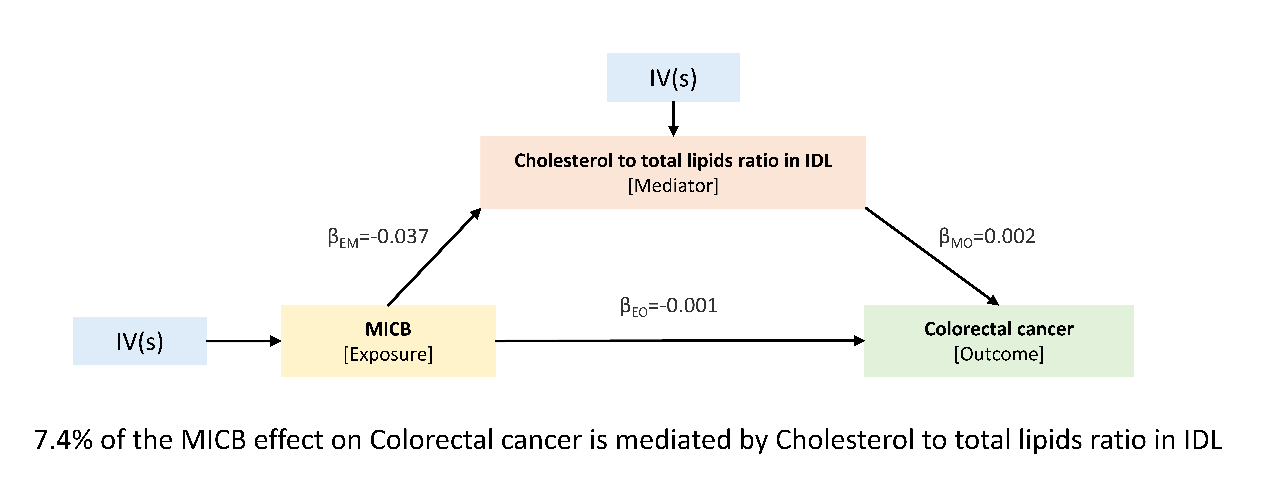

Supplement: Supplementary file 1 — Supplementary Material 1 [file 12885_2023_11669_MOESM1_ESM.docx]
